# Preparation of a benziodazole-type iodine(III) compound and its application as a nitrating reagent for synthesis of furazans via a copper-catalyzed cascade process

**DOI:** 10.1038/s42004-024-01238-8

**Published:** 2024-07-09

**Authors:** Zhifang Yang, Jun Xu, Yuli Sun, Xuemin Li, Bohan Jia, Yunfei Du

**Affiliations:** Tianjin Key Laboratory for Modern Drug Delivery & High-Efficiency, School of Pharmaceutical Science and Technology, Tianjin University, Tianjin, 300072 China

**Keywords:** Synthetic chemistry methodology, Structure elucidation, Synthetic chemistry methodology

## Abstract

The existing hypervalent I(III) reagents bearing ONO_2_ group are limited in types and their applications primarily focused on the nitrooxylation reactions featuring a fully-*exo* fashion. Herein, a benziodazole-type O_2_NO-I(III) compound was prepared and its reaction with β-monosubstituted enamines in the presence of CuI could trigger a radical nitration/cyclization/dehydration cascade to provide a series of less explored but biologically interesting furazan heterocycles. Mechanistically, the benziodazole-type O_2_NO-I(III) compound acts as a nitrating reagent and incorporates its NO moiety into the final furazan product in a fully-*endo* model, a process of which was proposed to involve nitration, cyclization and dehydration.

## Abstract (web summary)

Hypervalent iodine(III) reagents show promising properties in organic synthesis, however, the application of nitrooxyl (O2NO)-containing I(III) reagents remains underexplored. Here, the authors report the synthesis of a benziodazole-type O2NO-I(III) reagent, which can be applied to the synthesis of furazan heterocycles as a nitrating agent, by reaction with β-monosubstituted enamines *via* a copper-catalyzed radical nitration/cyclization/dehydration cascade.

## Introduction

**Fig. 1:**
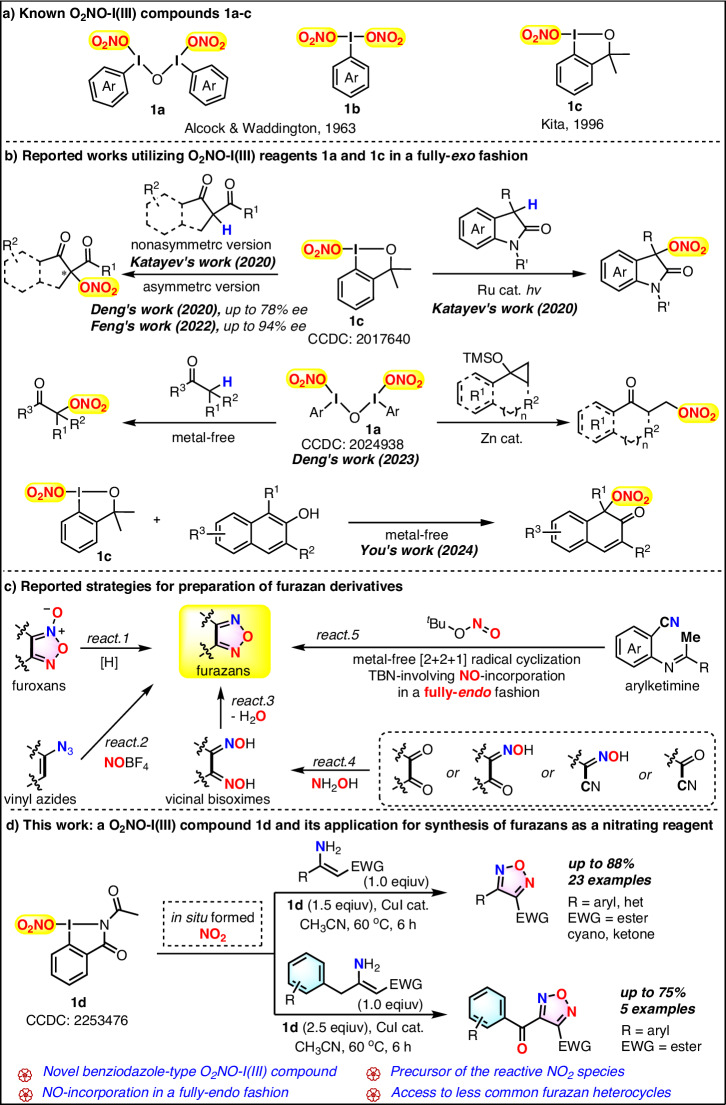
Existing applications of O_2_NO-I(III) compounds and known accesses to furazans. **a** Known O_2_NO-I(III) compounds **1a–c**. **b** Reported works utilizing O_2_NO-I(III) reagents **1a** and **1c** in a fully-*exo* fashion. **c** Reported strategies for the preparation of furazan derivatives. **d** This work: a O_2_NO-I(III) compound **1d** and its application for synthesis of furazans as a nitrating reagent.

In the past several decades, the development and application of hypervalent iodine(III) reagents have received considerable attention from organic chemists for their excellent properties^1–6^, including thermodynamic stability, environmental friendliness, and versatile reactivity^7–24^. In contrast to the most well-developed functional-group-transferring transformations enabled by trifluoromethyl-, fluoro-, azido-, alkynyl-, alkenyl-containing hypervalent iodine(III) reagents^7–16^, the application of the nitrooxyl (O_2_NO)-containing I(III) reagents has remained a challenge for organic chemists, as the existing O_2_NO-I(III) reagents **1a–c** had not found wide application in organic synthesis since their discovery several decades ago (Fig. 1a)^25,26^. It was not until 2020 that Katayev’s group realized the first application of O_2_NO-I(III) reagent **1c** in the preparation of nitrooxylated β-keto esters, 1,3-diketones, malonates, and oxindoles in the absence of oxidants or bases (Fig. 1b)^27^. It is worth mentioning that the asymmetric version of this transformation between the reaction of O_2_NO-I(III) reagent **1c** with β-keto esters and β-keto amides were further investigated by Deng^28^ and Feng’s groups (Fig. 1b)^29^, respectively. In 2023, Deng’s group further accomplished the nitrooxylation of diverse substrates including cyclopropyl silyl ethers, β-keto esters, β-keto amides, 1,3-diketones, and β-naphthol, by using noncyclic O_2_NO-I(III) compound **1a** as nitrooxylating reagent (Fig. 1b)^30^. In addition, a catalyst-free intermolecular dearomatization reaction of β-naphthols with reagent **1c** under mild conditions to access various nitrooxylated β-naphthalenones was uncovered by You’s group recently (Fig. 1b)^31^. Obviously, the existing hypervalent iodine(III) reagents bearing the ONO_2_ group are limited in types, and their applications primarily focused on the nitrooxylation reactions featuring a fully-*exo* fashion. In this regard, the development of hypervalent O_2_NO-containing iodine(III) reagents and searching for their other unique applications should be highly desirable.

Furazans (1,2,5-oxadiazoles)^32–36^ constitute an important class of heterocycles that have been applied as energetic materials^37–43^ and biologically active agents^44–48^. Accordingly, a great deal of effort has been devoted to the assemblage of this unique class of skeletons. However, the known strategies for accessing Furazans are relatively limited. Literature survey showed that the synthesis of furazans could be realized via deoxygenation of furoxans by tri-substituted phosphite (Fig. 1c, react. 1)^49–51^, cyclization of vinyl azides with NOBF_4_ (Fig. 1c, react. 2)^52^, and dehydrative cyclization of vicinal bisoximes (Fig. 1c, react. 3)^34–36,53–63^ mediated by alkalinous^34,53^ or acidic additives^54–61^, I_2_P_4_^62^ or PPh_3_/DIAD^63^. It is worth noting that this last strategy is the most widely-used methodology as vicinal bisoximes, the precursor of furazans, could be readily obtained from hydroxylamination of ammonia with glyoxals, glyoxal monooximes, cyano oximes, or acyl cyanides (Fig. 1c, react. 4)^34,64^. Additionally, Kwong’s group recently developed an expeditious metal-free [2 + 2 + 1] radical tandem cyclization reaction of arylketimine, realizing the synthesis of a series of furazan-fused quinolines by employing *tert*-butyl nitrite (^*t*^BuONO) to incorporate NO moiety into furazan framework in a fully-*endo* pattern (Fig. 1c, react. 5)^65^. Although all the above approaches have their respective merits in obtaining the corresponding furazan derivatives, the development of novel synthetic routes to access this unique heterocycle should still be of important synthetic value.

Here, we reported that benziodazole-type O_2_NO-I(III) **1d**, being a hypervalent O_2_NO-containing iodine(III) compound, could react with β-monosubstituted enamines to trigger a copper-catalyzed radical nitration/cyclization/dehydration cascade, providing an alternative protocol to access the exclusive furazan heterocycles (Fig. 1d). Differing from the previous nitrooxylation reactions enabled by the existing O_2_NO-I(III) reagents **1a** and **1c**, O_2_NO-I(III) compound **1d** in this work was used as nitrating reagent to incorporate its NO moiety to furazan skeleton in a fully-*endo* pattern.

## Results and discussion

**Fig. 2:**
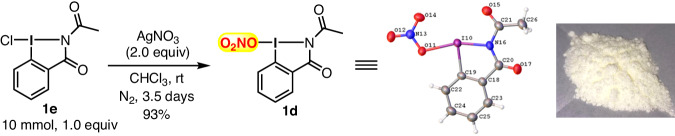
Preparation of O_2_NO-containing benziodazole-type I(III) 1d. Conversion to reagent **1d** from **1e** via ligand exchange reaction and single-crystal X-ray structure of **1d**.

In order to further enrich the type of hypervalent iodine(III) reagents^66,67^, we were interested in investigating the preparation of a benziodazole-bearing O_2_NO group. Following the general procedure^66–69^, a ligand exchange reaction of benziodazole-type Cl-I(III) compound **1e** with silver nitrate (AgNO_3_) was conducted in dried chloroform under an N_2_ atmosphere. The reaction afforded the expected benziodazole-type O_2_NO-I(III) 1d feasibly in 93% yield as a light yellow solid, which is stable for several months when stored at 0 °C in the absence of light (Fig. 2). Thermogravimetry-differential thermal analysis (TG-DTS) showed that compound **1d** decomposed at 180.7 °C (for details see Supplementary Data 3). Furthermore, a single crystal of **1d** was grown in a mixed solvent of chloroform/*n*-hexane at room temperature, and it crystallized in the monoclinic space group *P*2_1_/*c* with *Z* = 4. An X-ray crystal analysis of compound **1d** (Fig. 2) revealed a distorted T-shape geometry like the common hypervalent λ^3^-iodanes with an O11–I10–N16 bond angle of 162.32(8)° and I–ONO_2_ bond length of 2.336(2) Å. The length of the observed I–ONO_2_ bond in compound **1d** is longer than its analogous 1a and 1c, i.e., 2.311(3) Å^30^, and 2.283(2) Å^27^, respectively, suggesting reduced covalent character. Compound **1d** also possesses a planar geometry, as indicated by the torsion angles O14–N13–O11–I10 (8.7(3)°), I10–C19–C18–C20 (2.0(3)°), and O17–C20–N16–C21 (8.0(5)°) (for details see Supplementary Data 4).

Initially, our efforts were focused on studying the feasibility of the nitrooxylation reaction of O_2_NO-I(III) **1d** with β-monosubstituted enamine **2a** in the presence of 10 mol% CuI in acetonitrile at 50 °C under nitrogen atmosphere. Unexpectedly, it was not the nitrooxylating product but the heterocyclic furazan **3a** that was produced and isolated in 72% yield (Table 1, entry 1). The results of a solvent screening revealed that the reaction in other solvents, including DCE and 1.4-dioxane led to inferior yields of **3a** (Table 1, entries 2–3), while no desired product was observed when THF, DMF, or HFIP was used (Table 1, entries 4–6). The following catalysts screening showed that the reaction proceeded with significant efficiency when CuBr or CuSCN was applied (Table 1, entries 7–8). However, when other copper reagents including CuCl, Cu_2_O, CuBr_2_, Cu(OAc)_2_, or Cu(OTf)_2_ were used, product **2a** was obtained in relatively lower yield in each case (Table 1, entries 9–13). Other metal additives including FeBr_2_, PdCl_2_, Mn(OAc)_2_, Ni(acac)_2_, Co(acac)_2_, and RhCl(PPh_3_)_3_ were also investigated. All of them were proved to be compatible with this reaction except Co(acac)_2_ (for details see Supplementary Table S1). The result of a control reaction conducted in the absence of a copper catalyst provided no desired product, indicating that the copper catalyst is indispensable for the reaction to occur (Table 1, entry 14). Temperature was proved to be another important factor for an efficient transformation, with reaction run at 60 °C afforded the best outcome (Table 1, entries 15–17). Furthermore, the screening on dosage of O_2_NO-I(III) **1d** indicated that 1.5 equivalents of the hypervalent iodine(III) reagent were necessary for complete consumption of the starting enamine **2a** (Table 1, entries 18–19).

**Table 1:** Optimization of the reaction conditions^a,b^

Entry	1d (x eqiuv)	Catalyst	Solvent (mL)	T (°C)	Yield (%)^b^
1	1.5	CuI	MeCN	50	72
2	1.5	CuI	DCE	50	43
3	1.5	CuI	1,4-dioxane	50	59
4	1.5	CuI	THF	50	nd
5	1.5	CuI	DMF	50	nd
6	1.5	CuI	HFIP	50	nd
7	1.5	CuBr	MeCN	50	66
8	1.5	CuSCN	MeCN	50	63
9	1.5	CuCl	MeCN	50	53
10	1.5	Cu_2_O	MeCN	50	58
11	1.5	CuBr_2_	MeCN	50	60
12	1.5	Cu(OAc)_2_	MeCN	50	31
13	1.5	Cu(OTf)_2_	MeCN	50	47
14	1.5	FeBr_2_	MeCN	50	67
15	1.5	PdCl_2_	MeCN	50	23
16	1.5	Mn(OAc)_2_	MeCN	50	29
17	1.5	Ni(acac)_2_	MeCN	50	52
18	1.5	Co(acac)_2_	MeCN	50	nd
19	1.5	RhCl(PPh_3_)_3_	MeCN	50	14
20	1.5	none	MeCN	50	nd
21	1.5	CuI	MeCN	rt	trace
22	1.5	CuI	MeCN	30	22
23	1.5	CuI	MeCN	60	80
24	1.0	CuI	MeCN	60	64

^a^Reaction conditions: **2a** (0.3 mmol, 1.0 equiv), O_2_NO-I(III) compounds **1d** (x equiv), catalyst (10 mol%), solvent (4 mL), nitrogen atmosphere, T °C. ^b^Isolated yield of **3a**. *nd* no detection.

**Fig. 3:**
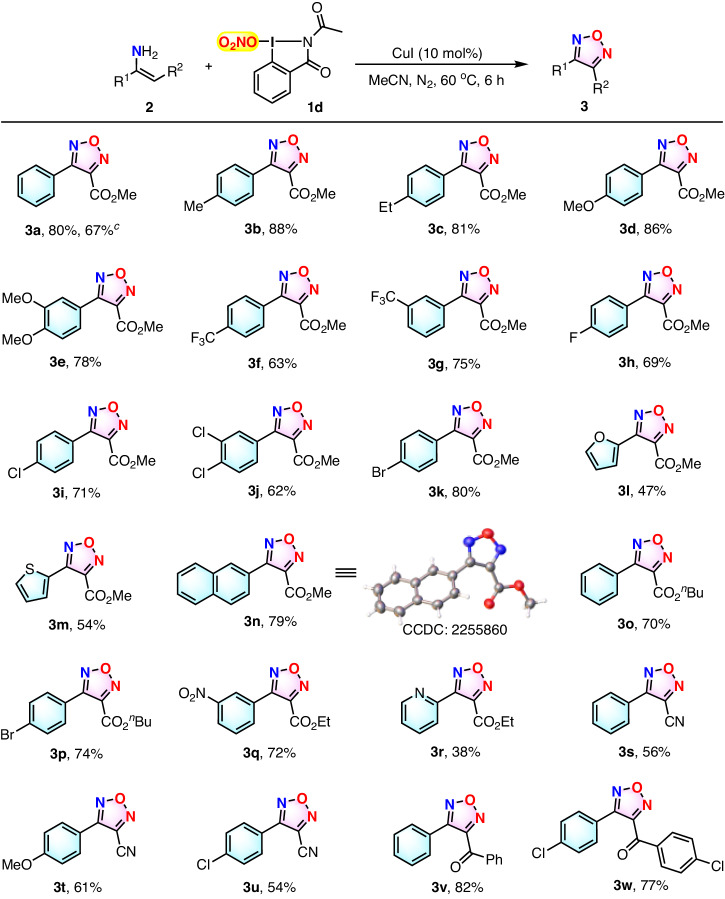
Substrate scope study for synthesis of furazans 3. ^[a]^ Reaction conditions: enamine **2** (0.3 mmol, 1.0 equiv), O_2_NO-I(III) **1d** (0.45 mmol, 1.5 equiv), CuI (10% mol) in acetonitrile (4 mL) under N_2_ atmosphere at 60 °C for 6 h. ^[b]^ Isolated yield. ^[c]^ Gram-scale synthesis of **3a**, 67% (10 mmol of **2a** was used).

With the optimized reaction conditions in hand (Table 1, entry 17), the substrate scope of this newly established method was evaluated (Fig. 3). A series of substituted enamines **2** were proved to be compatible with the protocol, with all of the reactions proceeded successfully to afford the corresponding substituted furazans **3a–w**. As can be seen from Fig. 3, aryl enamines substituted with electron-neutral, -donating, and -withdrawing groups reacted favorably to afford furazans **3a–g** in moderate to good yields. In addition, halogen-containing substrates were also conveniently converted to the desired products **3h–k** in satisfactory yields. In addition, enamines equipping heterocyclic furyl and thienyl groups, or an aromatic naphthyl substituent, were also suitable for this transformation, yields. Notably, the structure of product **3n** unambiguously provides the corresponding furazans **3l–n** in acceptable to good confirmed by X-ray single-crystal diffraction analysis (for details see Supplementary Data 5). Furthermore, the reaction of substrates bearing other alkoxycarbonyl substituents, such as butoxycarbonyl and ethoxycarbonyl group, also proceeded well with good efficiency (**3o–r**). Strikingly, the method could also be well applied to enamines containing the analogous electron-withdrawing cyano or aroyl substituents (**3s–w**). The utility of this method was further demonstrated by the gram-scale synthesis of compound **3a** in a yield of 67% when 10 mmol of **2a** was used under optimized conditions.

**Fig. 4:**
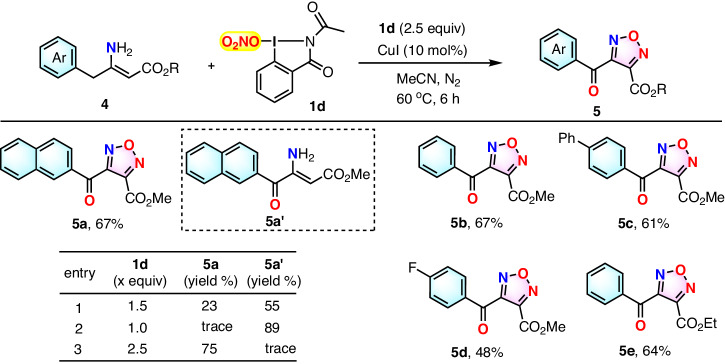
Substrate scope study for synthesis of furazans 5. ^[a]^ Reaction conditions: enamine **4** (0.3 mmol, 1.0 equiv), O_2_NO-I(III) **1d** (0.75 mmol, 2.5 equiv), CuI (10% mol) in acetonitrile (4 mL) under N_2_ atmosphere at 60 °C for 6 h. ^[b]^Isolated yield.

To our surprise, when the reaction of substrate **4a** with a menaphthyl moiety was conducted under the above-optimized reaction conditions, it was not the expected menaphthyl furazan but the benzylic CH_2_-oxidized compounds, i.e., naphthoyl furazan **5a** as well as its precursor **5a’** that were isolated in a yield of 23% and 55%, respectively (Fig. 4, entry 1). Further study revealed that reaction of menaphthyl-substituted enamine **4a** with 1.0 equiv of O_2_NO-I(III) **1d** in the presence of CuI catalyst under nitrogen atmosphere gave 89% naphthoyl-containing enamine **5a’** (Fig. 4, entry 2), which could be further converted to product **5a** under standard reaction conditions (see SI for details). Considering above facts as well as the result of the control reaction (see SI for details) where trace yield of **5a’** was formed in the absence of CuI catalyst (with most of starting materiel **4b** unconsumed), we tentatively presumed that enamine **4a** was first oxidized to benzoyl enamine **5a’** by O_2_NO-I(III) **1d** assisted by CuI catalyst and then the formed **5a’** was converted to product **5a** by further reacting with O_2_NO-I(III) **1d**. Thus, a larger amount of **1d** was employed to facilitate the complete conversion of enamine **4a** to furazan **5a**. When the amount of O_2_NO-I(III) **1d** was increased to 2.5 equivalents, a 75% yield of furazan **5a** was attained (Fig. 4, entry 3). Under the most optimal conditions, other benzyl enamines were investigated, and they were all converted to the corresponding furazans **5b–e** with acceptable yields (Fig. 4).

**Fig. 5:**
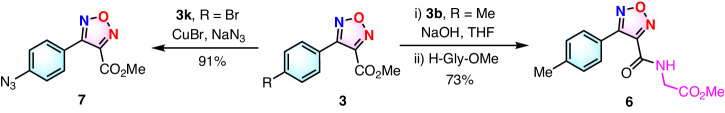
Further derivatization of the obtained furazans. Conversion to furazans 6–7 from compounds **3b** and **3k** via amidation and substitution, respectively.

Derivatization of the obtained furazan derivatives was carried out to prove the utility of this method (Fig. 5). To our delight, furazan **3b** could be further transformed into compound **6** via the one-pot two-step amidation^70^. In addition, azide **7** could be achieved from furazan **3k** through nucleophilic substitution reaction^71,72^. Both of the two transformations provided access to new derivatized furazan-containing molecules, demonstrating the stability of the exclusive furazan skeleton under the respective reaction conditions.

**Fig. 6:**
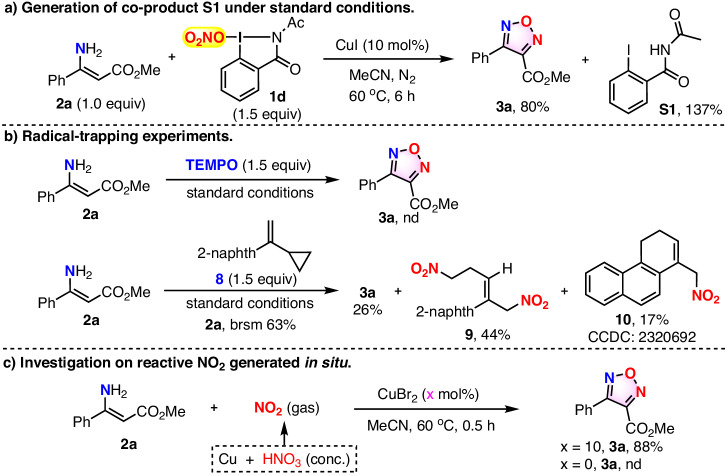
Mechanism investigation. **a** Generation of co-product S1 under standard conditions. **b** Radical-trapping experiments. **c** Investigation on reactive NO_2_ generated in situ.

To understand the mechanism of this copper-catalyzed O_2_NO-I(III) **1d**-mediated transformation, a series of control experiments were conducted (Fig. 6). First, the reaction of substrate **2a** under standard conditions produced co-product *N*-acetyl-2-iodobenzamide **S1** in high yield (based on starting material **2a**) (Fig. 6a). Then radical scavenger was introduced to investigate whether the reaction adopts a radical pathway. Specifically, when 1.5 equivalents of TEMPO was employed under standard conditions, the transformation was almost completely inhibited (Fig. 6b). Next, a radical clock experiment was carried out by introducing 1.5 equivalents of compound **8** to the reaction of substrate **2a** under standard reactions, and it was found that furazan **3a**, nitrated compounds **9** and **10** were isolated in yield of 26%, 44%, 17%, respectively (Fig. 6b). The outcomes of the above experiments strongly indicate that reaction process might encompass a radical pathway, and the reactive NO_2_ species^73–76^ might be a crucial intermediate formed in situ from O_2_NO-I(III) **1d** during the process. To corroborate whether NO_2_ was the intermediate, control experiment by replacing O_2_NO-I(III) **1d** with exogenous brown NO_2_ gas, generated from the known reaction^77,78^ of copper powder and concentrated nitric acid, were conducted (Fig. 6c). To our delight, furazan **3a** was obtained in 88% from the reaction of treating substrate **2a** with NO_2_ gas in presence of CuBr_2_ catalyst in acetonitrile at 60 °C for 0.5 h, while no **3a** was detected when no copper catalyst was used. The result of the above control experiment strongly supports our assumption that NO_2_ is the reactive species that enables the nitration reaction to occur.

**Fig. 7:**
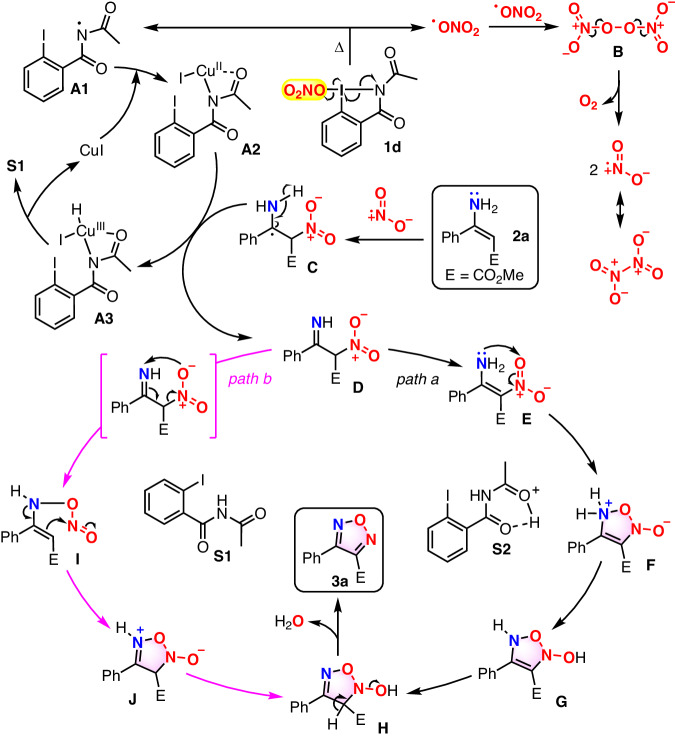
Plausible mechanism. The formation of NO_2_ and the following NO_2_-radical addition/cyclization/dehydration cascade.

Based on the above results as well as the previous reports^73–76,79–83^, a plausible radical pathway including two parts (the formation of NO_2_ and the following NO_2_-radical addition/cyclization/dehydration cascade) was proposed for this transformation (Fig. 7). Initially, homolysis^79–83^ of O_2_NO-I(III) **1d** under heating gives O_2_NO radical and *N*-radical **A1**. Single electron oxidation of CuI by **A1** affords Cu(II) species **A2**. Meanwhile, dimerization of the generated O_2_NO radical generates intermediate **B**, which is unstable and undergoes dissociation to release oxygen gas as well as NO_2_ molecule, a reactive radical species that can dimerize into N_2_O_4_. Then, the radical addition of NO_2_ to the C–C double bond of enamine **2a** furnishes radical species **C**. Next, one H radical of intermediate **C** is captured by Cu(II) species **A2**, leading to the formation of imine **D** as well as Cu(III) species **A3**, which undergoes reductive elimination to form *N*-acetyl-2-iodobenzamide **S1** and CuI.

Next, two pathways (Fig. 7, the path a and b) were postulated for the formation of furazan **3a** from intermediate **D**. In path a, enamine **E** was formed from imine **D** via tautomerism first. Then nucleophilic attack of the nitrogen atom of enamino moiety in intermediate **E** to its oxygen center of nitrone gave the cyclized intermediate **F**. Subsequent tautomerization of **F** achieved via the system of **S1**/**S2** and following dehydration of the resulting intermediate **H** gave product **3a**. While in path b, the intramolecular attack of oxygen atom of nitrone in intermediate **D** to nitrogen center of its imino moiety, with the concomitant formation of C–C double bond and cleavage of C–N bond occurred first to give intermediate **I**. Then intramolecular cyclization of **I** provided the cyclized intermediate **J**, which underwent similar tautomerization and following dehydration of the resulting intermediate **H** to afford furazan **3a**.

**Fig. 8:**
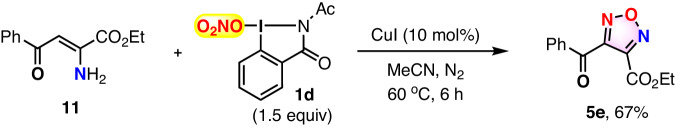
Reaction utilizing enaminone 11 as starting material. Compound **11** was also a suitable substrate for our nitration/cyclization/dehydration cascade reaction.

Finally, enaminone **11** was also examined to explore whether it is applicable to this radical nitration/cyclization/dehydration cascade reaction. The results showed that enamine **11** was equally applicable for this transformation under standard conditions, furnishing furazan **5e** in 76% yield (Fig. 8). Interestingly, the two N atoms in product **5e** originate from the amino moiety of enamine **11** and the nitrooxyl moiety of O_2_NO-I(III) **1d**, respectively, but in a completely reversed pattern to the ones of **5e** generated from **4e** (Fig. 4).

In conclusion, we prepared a benziodazole-type hypervalent O_2_NO-I(III) compound **1d** and had it applied to the synthesis of a series of exclusively heterocyclic furazans from β-monosubstituted enamines via an unprecedented copper-catalyzed radical nitration/cyclization/dehydration cascade. Differing from the existing O_2_NO-I(III) reagents that have been uniformly used as nitrooxylating reagents for introducing O_2_NO moiety, the O_2_NO-I(III) 1d described in this work can be regarded as a nitrating reagent and incorporate its NO moiety to furazan skeleton in a fully-*endo* pattern. Furthermore, the current method also provides an alternative approach, which is in nature different from the existing strategies^34,49–65^, to the biologically interesting furazan heterocycles.

## Methods

### Procedure for synthesis of O_2_NO-Iodine(III) 1d

To a 200 mL two-necked round-bottomed flask were added compound **1e** (3.23 g, 10 mmol, 1.0 equiv), AgNO_3_ (3.4 g, 20 mmol, 2.0 equiv) and dried CHCl_3_ (70 mL) under N_2_ atmosphere. The reaction mixture was stirred at room temperature in the dark for 3.5 days. The mixture was then filtered through a pad of Celite and washed with CHCl_3_ (1000 mL). The solvents were concentrated in a vacuum to give compound **1d** as a white solid.

### General procedure for the synthesis of substituted furazans 3 and 5

To a 20 mL Schlenk tube equipped with a stirrer was added β-monosubstituted enamine **2** (0.3 mmol, 1.0 equiv), O_2_NO-I(III) **1d** (0.45 mmol, 1.5 equiv) and CuI (0.03 mmol, 6 mg, 10 mol%) under N_2_ atmosphere, followed by addition of acetonitrile (4 mL). The tube was screw-capped and stirred at 60 °C. After stirring for 6 h, the reaction mixture was diluted with dichloromethane, filtered through a pad of Celite, and concentrated in a vacuum. The residue was purified with silica gel chromatography (PE/EtOAc) to afford furazans **3**. (When enamine **4** and O_2_NO-I(III) **1d** (0.75 mmol, 2.5 equiv) were employed as starting materials under the above conditions, substituted furazans **5** was obtained).

### Supplementary information

Peer Review File

Supporting Information

Description of Additional Supplementary Files

Supplementary Data 1

Supplementary Data 2

Supplementary Data 3

Supplementary Data 4

Supplementary Data 5

Supplementary Data 6

## Data Availability

All data generated during this study are included in this article and Supplementary Information. Experimental procedure, conditions optimization and product characterization are provided in the Supplementary Information. The NMR spectra of all compounds are available in Supplementary Data 1. The infrared spectra of compound **1d** is available in Supplementary Data 2. The TG-DTA and DSC analysis of compound **1d** is available in Supplementary Data 3. The crystallographic data for compounds **1d**, **3n**, and **10** can be obtained free of charge from the Cambridge Crystallographic Data Centre (CCDC) under deposition numbers 2253476 (**1d**, Supplementary Data 4), 2255860 (**3n**, Supplementary Data 5) and 2320692 (**10**, Supplementary Data 6), respectively. These data can be obtained free of charge from the Cambridge Crystallographic Data Centre via www.ccdc.cam.ac.uk/data_request/cif.

## References

[1] Koser GF (2003). The synthesis of heterocyclic compounds with hypervalent organoiodine reagents. Adv. Heterocycl. Chem..

[2] Moriarty RM (2005). Organohypervalent iodine: development, applications, and future directions. J. Org. Chem..

[3] Kohlhepp SV, Gulder T (2016). Hypervalent iodine(III) fluorinations of alkenes and diazo compounds: new opportunities in fluorination chemistry. Chem. Soc. Rev..

[4] Yoshimura A, Zhdankin VV (2016). Advances in synthetic applications of hypervalent iodine compounds. Chem. Rev..

[5] Morales-Rojas H, Moss RA (2002). Phosphorolytic reactivity of *o*-iodosylcarboxylates and related nucleophiles. Chem. Rev..

[6] Yang Z, Du F-H, Zhang C, Du Y (2023). Accessing aryl azides via copper powder-catalyzed cross-coupling of arylboronic acids with the hypervalent azido-iodine reagent ABZ(I). Org. Chem. Front..

[7] Kieltsch I, Eisenberger P, Togni A (2007). Mild electrophilic trifluoromethylation of carbon- and sulfur-centered nucleophiles by a hypervalent iodine(III)–CF_3_ reagent. Angew. Chem. Int. Ed..

[8] Yang J-D, Li M, Xue X-S (2019). Computational I(III)—X BDEs for benziodoxol(on)e-based hypervalent iodine reagents: implications for their functional group transfer abilities. Chin. J. Chem..

[9] Ilchenko NO, Tasch BOA, Szabó KJ (2014). Mild silver-mediated geminal difluorination of styrenes using an air- and moisture-stable fluoroiodane reagent. Angew. Chem. Int. Ed..

[10] Kiefl GM, Gulder T (2020). α-Functionalization of ketones via a nitrogen directed oxidative umpolung. J. Am. Chem. Soc..

[11] Zhdankin VV, Kuehl CJ, Krasutsky AP, Formaneck MS, Bolz JT (1994). Preparation and chemistry of stable azidoiodinanes: 1-azido-3,3-bis(trifluoromethyl)-3-(1*H*)-1,2-benziodoxol and 1-azido-1,2-benziodoxol-3-(1*H*)-one. Tetrahedron Lett..

[12] Zhdankin VV (1996). Preparation, X-ray crystal structure, and chemistry of stable azidoiodinanes-derivatives of benziodoxole. J. Am. Chem. Soc..

[13] Brand JP, Charpentier J, Waser J (2009). Direct alkynylation of indole and pyrrole heterocycles. Angew. Chem. Int. Ed..

[14] Mironova IA, Noskov DM, Yoshimura A, Yusubov MS, Zhdankin VV (2023). Aryl-, akynyl-, and alkenylbenziodoxoles: synthesis and synthetic applications. Molecules.

[15] Stang PJ (1992). Alkynyl- and alkenyl(phenyl)iodonium compounds. New synthetic methods. Angew. Chem. Int. Ed..

[16] Stridfeldt E (2016). Synthesis, characterization and unusual reactivity of vinylbenziodoxolones-novel hypervalent iodine reagents. Chem. Eur. J..

[17] Declas N, Vaillant FL, Waser J (2019). Revisiting the urech synthesis of hydantoins: direct access to enantiopure 1,5-substituted hydantoins using cyanobenziodoxolone. Org. Lett..

[18] Genoux A, González JA, Merino E, Nevado C (2020). Mechanistic insights into C(sp^2^)−C(sp)N reductive elimination from gold(III) cyanide complexes. Angew. Chem. Int. Ed..

[19] Wang X, Yang T, Cheng X, Shen Q (2013). Enantioselective electrophilic trifluoromethylthiolation of β-ketoesters: a case of reactivity and selectivity bias for organocatalysis. Angew. Chem. Int. Ed..

[20] Shao X, Wang X, Yang T, Lu L, Shen Q (2013). An electrophilic hypervalent iodine reagent for trifluoromethylthiolation. Angew. Chem. Int. Ed..

[21] Vinogradova EV, Müller P, Buchwald SL (2014). Structural reevaluation of the electrophilic hypervalent iodine reagent for trifluoromethylthiolation supported by the crystalline sponge method for X-ray analysis. Angew. Chem. Int. Ed..

[22] Xiao J-A (2019). Selenocyanobenziodoxolone: a practical electrophilic selenocyanation reagent and its application for solid-state synthesis of α-carbonyl selenocyanates. Org. Chem. Front..

[23] Liu Z, Wu S, Chen Y (2021). Selective C(sp^3^)-C(sp^3^) Cleavage/alkynylation of Cycloalkylamides Enables Aminoalkyne Synthesis with Hypervalent Iodine Reagents. ACS Catal..

[24] Liu Z (2022). Hypervalent iodine reagents enable C-H alkynylation with iminophenylacetic acids via alkoxyl radicals. Org. Lett..

[25] Alcock, N. W. & Waddington, T. C. 780. Chemistry of positive iodine. Part II. Reactions of iodobenzene dichloride with silver salts. *J. Chem. Soc*. 4103–4109 (1963).

[26] Akai S (1996). Preparation of novel cyclic hypervalent idoine(III) compounds having azido, cyano, and nitrato ligands. Heterocycles.

[27] Calvo R (2020). Synthesis, characterization, and reactivity of a hypervalent-iodine-based nitrooxylating rreagent. Angew. Chem. Int. Ed..

[28] Li B (2020). Zinc-catalyzed asymmetric nitrooxylation of β-keto esters/amides with a benziodoxole-derived nitrooxy transfer reagent. Org. Chem. Front..

[29] He C, Wu Z, Zhou Y, Cao W, Feng X (2022). Asymmetric catalytic nitrooxylation and azidation of β-keto amides/esters with hypervalent iodine reagents. Org. Chem. Front..

[30] Cheng X (2023). Simple and versatile nitrooxylation: noncyclic hypervalent iodine nitrooxylating reagent. Angew. Chem. Int. Ed..

[31] Zhang, S.-S., Li, M., Gu, Q. & You, S.-L. Nitrooxylative dearomatization reaction of β-naphthols with hypervalent iodine reagent. *Asian J. Org. Chem*. **13**, e202400005 (2024).

[32] Olofson RA, Thompson WR, Michelman JS (1964). Heterocyclic nitrogen ylides. J. Am. Chem. Soc..

[33] Olofson RA, Michelman JS (1965). Furazan. J. Org. Chem..

[34] Sheremetev AB, Makhova NN, Friedrichsen W (2001). Monocyclic furazans and furoxans. Adv. Heterocycl. Chem..

[35] Makhova NN (2020). Progress in the chemistry of nitrogen-, oxygen- and sulfur-containing heterocyclic systems. Russ. Chem. Rev..

[36] Cao W-L, Li Z-M, Yang J-Q, Zhang J-G (2022). Recent advances on the nitrogen-rich 1,2,4-oxadiazole-azoles-based energetic materials. Def. Technol..

[37] Wang R, Guo Y, Zeng Z, Twamley B, Shreeve JM (2009). Furazan-functionalized tetrazolate-based salts: a new family of insensitive energetic materials. Chem. Eur. J..

[38] Wang B, Zhang G, Huo H, Fan Y, Fan X (2011). Synthesis, characterization and thermal properties of energetic compounds derived from 3-amino-4-(tetrazol-5-yl)furazan. Chin. J. Chem..

[39] Tang Y, Zhang J, Mitchell LA, Parrish DA, Shreeve JM (2015). Taming of 3,4-di(nitramino)furazan. J. Am. Chem. Soc..

[40] Zhang J, Dharavath S, Mitchell LA, Parrish DA, Shreeve JM (2016). Bridged bisnitramide-substituted furazan-based energetic materials. J. Mater. Chem. A.

[41] Xu Z (2017). A facile and versatile synthesis of energetic furazan-functionalized 5-nitroimino-1,2,4-triazoles. Angew. Chem. Int. Ed..

[42] Zhang J, Zhou J, Bi F, Wang B (2020). Energetic materials based on poly furazan and furoxan structures. Chin. Chem. Lett..

[43] Liu Y (2023). Three-dimensional metal-organic frameworks as super heat-resistant explosives: potassium 4,4’-oxybis[3,3’-(5-tetrazol)]furazan and potassium (1,2,4-triazol-3-yl)tetrazole. Inorg. Chem..

[44] Mataka S, Takahashi K, Imura T, Tashiro M (1982). Reduction of 4,7-diphenyl-1,2,5-thia(oxa)diazolo[3,4-*c*]pyridines affording 2,5-diphenyl-3,4-diaminopyridines and ring closure of the diamines to fluorescent azaheterocycles. J. Heterocycl. Chem..

[45] Kulikov AS (2013). Synthesis and antineoplastic properties of (1*H*-1,2,3-Triazol-1-yl)furazans. Russ. Chem. Bull..

[46] Yue EW (2017). INCB24360 (epacadostat), a highly potent and selective indoleamine-2,3-dioxygenase 1 (IDO1) inhibitor for immuno-oncology. ACS Med. Chem. Lett..

[47] Chen T (2019). Synthesis and characterization of furazan derivatives and their evaluation as antitumor agents. Chem. Pap..

[48] Mancini RS, Barden CJ, Weaver DF, Reed MA (2021). Furazans iN Medicinal Chemistry. J. Med. Chem..

[49] Grundmann C (1964). Über die spezifische reduktion von furoxanen zu furazanen. Chem. Ber..

[50] Boulton, A. J., Hadjimihalakis, P., Katritzky, A. R. & Hamid, A. M. *N*-oxides and related compounds. Part XXXVI. Isomerism in the oxadiazole series. *J. Chem. Soc. C* **14**, 1901–1903 (1969).

[51] Tironi C, Calvino R, Menziani E, Carazzone M (1984). Furazan sulfanilamides. Farm. Sci..

[52] Thakore AN, Buchshriber J, Oehlschlager AC (1973). Vinyl azides as diazoenamines. Can. J. Chem..

[53] Neel AJ, Zhao R (2018). Mild synthesis of substituted 1,2,5-oxadiazoles using 1,1’-carbonyldiimidazole as a dehydrating agent. Org. Lett..

[54] Shaposhnikov SD (2003). Ring-opening and recyclization of 3,4-diacylfuroxans by nitrogen nucleophiles. Tetrahedron.

[55] Samsonov VA, Sal’nikov GE, Genayev AM (2009). Synthesis of 1-hydroxybenzotriazoles angularly annulated by furazan or furoxan rings. Russ. Chem. Bull..

[56] Tokura N, Tada R, Yokoyama K (1961). Formation of cyclohexano[*c*]1,2,5-oxadiazole from 1,2-cyclohexanedione dioxime. An attempted beckmann rearrangement with thionyl chloride in liquid sulfur dioxide. Bull. Chem. Soc. Jpn.

[57] Tokura N, Shirai I, Sugahara T (1962). The reactions of 1,2-cyclopentanedione dioxime and of 1,2,3- and 1,3,5-cyclohexanetrione trioximes in liquid sulfur dioxide. Bull. Chem. Soc. Jpn.

[58] Ohta G, Takegoshi T, Ueno K, Shimizu M (1965). Investigations on steroids. IV. Syntheses of androstano[2,3-*c*]-furazans and related compounds. Chem. Pharm. Bull..

[59] Olofson RA, Michelman JS (1964). Furazans and furazanium salts. J. Am. Chem. Soc..

[60] Britsun VN, Borisevich AN, Samoilenko LS, Lozinskii MO (2005). Synthesis of 3-(6-R-Benzothiazol-2-yl)-4-methyl-1,2,5-oxadiazoles. Russ. J. Org. Chem..

[61] Kamitori Y (1999). A convenient and facile synthesis of 3-trifluoromethyl-1,2,5-oxadiazoles with the use of silica gel as an effective catalyst. Heterocycles.

[62] Telvekar VN, Takale BS (2013). Reaction of oximes of α-diketones with diphosphorous tetraiodide for preparation of oxadiazoles and nitriles. Synth. Commun..

[63] Tron GC, Pagliai F, Del Grosso E, Genazzani AA, Sorba G (2005). Synthesis and cytotoxic evaluation of combretafurazans. J. Med. Chem..

[64] Andrianov VG, Eremeev AV (1984). Aminofurazans. Chem. Heterocycl. Compd..

[65] Deng G, Zhong R, Song J, Choy PY, Kwong FY (2021). Assembly of furazan-fused quinolines via an expeditious metal-free [2+2+1] radical tandem cyclization process. Org. Lett..

[66] Yang Z (2023). Preparation, structural identification and reactivities of two benziodazole-type I(III) reagents. Adv. Synth. Catal..

[67] Zhang G (2020). A new hypervalent iodine(III/V) oxidant and its application to the synthesis of 2*H*-azirines. Chem. Sci..

[68] Yang XG, Zheng K, Zhang C (2020). Electrophilic hypervalent trifluoromethylthio-iodine(III) reagent. Org. Lett..

[69] Ren J (2021). Ring expansion fluorination of unactivated cyclopropanes mediated by a new monofluoroiodane(III) reagent. Angew. Chem. Int. Ed..

[70] Yang Z-F, Xu C, Zheng X, Zhang X (2020). Nickel-catalyzed carbodifunctionalization of *N*-vinylamides enables access to γ-amino acids. Chem. Commun..

[71] Yang X (2007). Synthesis of difluoromethylene-containing 1,2,4-oxadiazole compounds via the reaction of 5-(difluoroiodomethyl)-3-phenyl-1,2,4-oxadiazole with unsaturated compounds initiated by sodium dithionite. Synthesis.

[72] Lamarque J-F (2008). Copper catalyzed 1,3-dipolar cycloaddition reaction of azides with *N*-(2-trifluoroacetylaryl) propargylamines: a mild entry to novel 1,4-disubstituted-[1,2,3]-triazole derivatives. J. Fluor. Chem..

[73] Maity S (2013). Efficient and stereoselective nitration of mono-and disubstituted olefins with AgNO_2_ and TEMPO. J. Am. Chem. Soc..

[74] Fan Z, Ni J, Zhang A (2016). Meta-selective C_Ar_–H nitration of arenes through a Ru_3_(CO)_12_-catalyzed *ortho*-metalation strategy. J. Am. Chem. Soc..

[75] Parrino F, Livraghi S, Giamello E, Palmisano L (2018). The existence of nitrate radicals in irradiated TiO_2_ aqueous suspensions in the presence of nitrate ions. Angew. Chem. Int. Ed..

[76] Huang J, Ding F, Rojsitthisak P, He F-S, Wu J (2020). Recent advances in nitro-involved radical reactions. Org. Chem. Front..

[77] Kirovskaya IA, Mironova EV, Bykova EI, Timoshenko OT, Filatova TN (2008). Adsorption and electrophysical studies of the sensitivity and selectivity of the surface of the InSb-CdTe system with respect to toxic gases. Russ. J. Phys. Chem..

[78] Rilyanti M, Hadi S (2011). Synthesis, characterization and thermal stability of complex *Cis*-[Co(bipy)_2_(CN)_2_] and its interaction with NO_2_ gas. Russ. J. Inorg. Chem..

[79] Li Y, Gao L-X, Han F-S (2010). Reliable and diverse synthesis of aryl azides through copper-catalyzed coupling of boronic acids or esters with TMSN_3_. Chem. Eur. J..

[80] Wang Y, Li G-X, Yang G, He G, Chen G (2016). A visible-light-promoted radical reaction system for azidation and halogenation of tertiary aliphatic C–H bonds. Chem. Sci..

[81] Rabet PTG, Fumagalli G, Boyd S, Greaney MF (2016). Benzylic C-H azidation using the zhdankin reagent and a copper photoredox catalyst. Org. Lett..

[82] Shinomoto Y (2015). Tetra-*n*-butylammonium iodide catalyzed C-H azidation of aldehydes with thermally stable azidobenziodoxolone. Org. Lett..

[83] Muriel B, Waser J (2021). Azide radical initiated ring opening of cyclopropenes leading to alkenyl nitriles and polycyclic aromatic compounds. Angew. Chem. Int. Ed..

