# Supplementary material for: Preparation of a benziodazole-type iodine(III) compound and its application as a nitrating reagent for synthesis of furazans via a copper-catalyzed cascade process
Source: Commun Chem. 2024 Jul 9;7:155. doi: 10.1038/s42004-024-01238-8 (PMC11233585; doi:10.1038/s42004-024-01238-8)
Supplement: Supplementary file 2 — Supporting Information [file 42004_2024_1238_MOESM2_ESM.pdf]

Preparation of A Benziodazole-type Iodine(III) Compound and Its Application as A Nitrating Reagent for Synthesis of Furazans via a Copper-catalyzed Cascade Process

Zhifang Yang, Jun Xu, Yuli Sun, Xuemin Li, Bohan Jia, and Yunfei Du*

Tianjin Key Laboratory for Modern Drug Delivery & High-Efficiency, School of Pharmaceutical Science and Technology, Tianjin University, Tianjin 300072, China. E-mail: duyunfeier@tju.edu.cn

Supporting Information

List of Contents

Supplementary Methods		S1
**I**	General Information	S1
**II**	Experimental Procedures and Spectroscopic Data	S1-S35
**III**	FTIR spectrum of I(III)-ONO_2 **1d**	S36
**IV**	Thermogravimetric and Differential Scanning Calorimetry (TGA-DSC) Profile of Compound **1d**	S37
**V**	X-Ray Diffraction Data of Compounds **1d**, **3n** and **10**	S38-S49
**VI**	Supplementary References	S50-S51

Supplementary Methods

I. General Information

^{1}H and $^{13}C\{^{1}H\}$ NMR spectra were recorded on a 400 MHz or 600 MHz spectrometer at 25 °C. Chemical shifts values are given in ppm and referred as the internal standard to TMS: 0.00 ppm. Chemical shifts were expressed in parts per million (δ) downfield from the internal standard tetramethylsilane, and were reported as s (singlet), d (doublet), t (triplet), q (quadruple), dd (doublet of doublet), m (multiplet), etc. The coupling constants *J*, are reported in Hertz (Hz). High resolution mass spectrometry (HRMS) data were recorded on Q Exactive HF (Q Exactive™ HF/UltiMate™ 3000 RSLCnano) using electron spray ionization (ESI) in positive (or negative) mode. The infrared spectra of the samples were collected using a Tensor 27 FT-IR spectrophotometer (Bruker, Ettlingen, Germany). The TG-DTA and DSC analysis were conducted by Rigaku Thermo plus EVO2 TG8121. Melting points were determined with a Micromelting point apparatus. TLC plates were visualized by exposure to ultraviolet light.

Reagents and solvents were purchased as reagent grade and were used without further purification. All reactions were performed in standard glassware, heated at 70 °C for 3 h before used. Flash column chromatography was performed over silica gel (200-300 m) using a mixture of ethyl acetate (EtOAc) and petroleum ether (PE).

II. Experimental Procedures and Spectroscopic Data

1. Optimization of the Reaction Conditions for Preparation of 3a.

To a 20 mL of Schlenk tube equipped with a stirrer was added β-monosubstituted enamine **2a** (0.3 mmol, 53 mg, 1.0 equiv), **1d** (0.45 mmol, 158 mg, 1.5 equiv) and metal catalyst (0.03 mmol, 10 mol%) under N_2 atmosphere, followed by addition of acetonitrile (4 mL). The tube was screw-capped and stirred at T ^{o}C. After stirring for 6 h, the reaction mixture was diluted with DCM, filtered through a pad of Celite and concentrated in vacuum. The residue was purified with silica gel chromatography (PE/EtOAc = 25:1) to afford **3a**.

Table S1. Optimization of the reaction conditions[a,b]

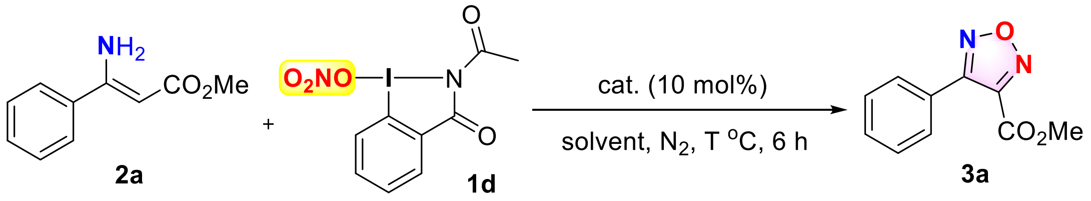

Entry	**1d** (x eqiuv)	Catalyst	Solvent (mL)	T (°C)	Yield (%)[b]
1	1.5	CuI	MeCN	50	72
2	1.5	CuI	DCE	50	43
3	1.5	CuI	1,4-dioxane	50	59
4	1.5	CuI	THF	50	nd
5	1.5	CuI	DMF	50	nd
6	1.5	CuI	HFIP	50	nd
7	1.5	CuBr	MeCN	50	66
8	1.5	CuSCN	MeCN	50	63
9	1.5	CuCl	MeCN	50	53
10	1.5	Cu_2O	MeCN	50	58
11	1.5	$CuBr_2$	MeCN	50	60
12	1.5	$Cu(OAc)_2$	MeCN	50	31
13	1.5	$Cu(OTf)_2$	MeCN	50	47
14	1.5	$FeBr_2$	MeCN	50	67
15	1.5	$PdCl_2$	MeCN	50	23
16	1.5	$Mn(OAc)_2$	MeCN	50	29
17	1.5	$Ni(acac)_2$	MeCN	50	52
18	1.5	$Co(acac)_2$	MeCN	50	nd
19	1.5	$RhCl(PPh_3)_3$	MeCN	50	14
20	1.5	none	MeCN	50	nd
21	1.5	CuI	MeCN	rt	trace
22	1.5	CuI	MeCN	30	22
23	1.5	CuI	MeCN	60	80
24	1.0	CuI	MeCN	60	64

[a]Reaction conditions: **2a** (0.3 mmol, 1.0 equiv), O_2NO-I(III) compounds **1d** (x equiv), catalyst (10 mol%), solvent (4 mL), nitrogen atmosphere, T °C. [b] Isolated yield of **3a**. nd = no detection.

2. General Procedure and Characterization Data of O_2NO-Iodine(III) 1d.

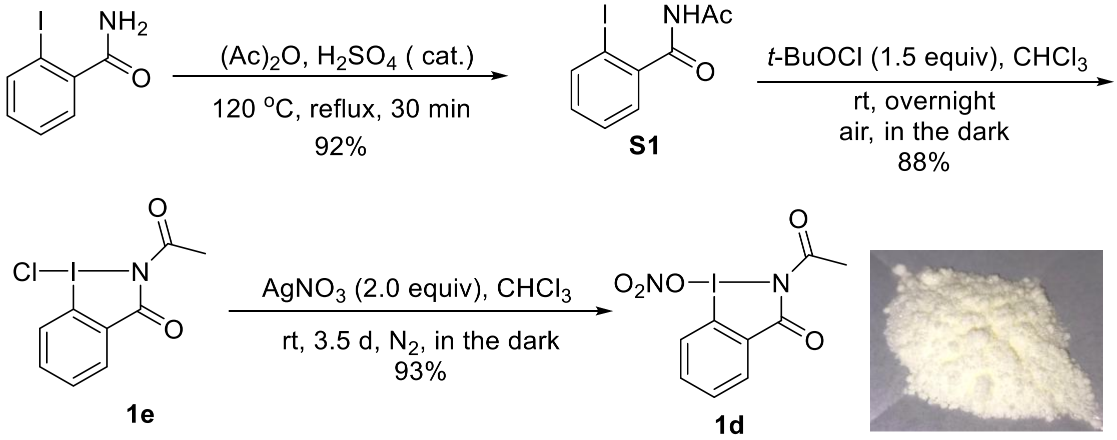

Preparation of ***N*-acetyl-2-iodobenzamide (S1)**:[1] To a 150 mL round-bottomed flask were added 2-iodobenzamide (20 mol, 5.0 g, 1.0 equiv), acetic anhydride (10 mL), and 2 drops of conc. H_2SO_4. Then the reaction mixture was heated to 120 °C and refluxed in the dark. The reaction progress was monitored by TLC. After the starting material was completely consumed, the reaction mixture was cooled to room temperature and was neutralized with aq. $NaHCO_3$ and extracted with EtOAc (3 × 120 mL), dried over anhydrous $MgSO_4$ and concentrated in vacuum. The crude material was purified by flash column chromatography with PE and EtOAc (v/v = 5:1) to give compound **S1** as a white solid (5.32 g, 92%). ^{1}H NMR (400 MHz, $CDCl_3$) δ 8.59 (br, 1H), 7.90 (d, *J* = 8.0 Hz, 1H), 7.42 (d, *J* = 4.4 Hz, 2H), 7.20 – 7.12 (m, 1H), 2.54 (s, 3H). ^{13}C{^{1}H} NMR (100 MHz, $CDCl_3$) δ 172.5, 167.7, 140.3, 134.0, 132.2, 128.3, 128.2, 91.9, 25.4.

Preparation of **2-acetyl-1-chloro-1,2-dihydro-3*H*-1λ^3-benzo[*d*][1,2]iodazol-3-one (1e)**:[1] To a 250 mL round-bottomed flask were added compound **S1** (18.4 mmol, 5.3 g, 1.0 equiv), *t*-BuOCl (28 mmol, 3.2 mL, 1.5 equiv) and $CHCl_3$ (100 mL). The reaction mixture was stirred at room temperature overnight in the dark under air. The precipitate was filtered and washed with *n*-hexane (50 mL) to give compound **1e** as a white solid (5.2 g, 88%). ^{1}H NMR (400 MHz, $CDCl_3$) δ 8.49 (dd, *J* = 9.0, 0.4 Hz, 1H), 8.21 (dd, *J* = 7.6, 1.6 Hz, 1H), 7.94 (td, *J* = 8.8, 1.6 Hz, 1H), 7.78 (td, *J* = 7.6, 0.8 Hz, 1H), 2.67 (s, 3H). ^{13}C{^{1}H} NMR (100 MHz, $CDCl_3$) δ 175.5, 161.5, 136.8, 134.4, 132.4, 131.6, 128.3, 114.0, 25.6.

Preparation of **2-acetyl-3-oxo-2,3-dihydro-1*H*-1λ^3-benzo[*d*][1,2]iodazol-1-yl nitrate (1d)**: To a 200 mL two-necked round-bottomed flask were added compound **1e** (10 mmol, 3.23 g, 1.0 equiv), $AgNO_3$ (20 mmol, 3.4 g, 2.0 equiv) and dried $CHCl_3$ (70 mL) under N_2 atmosphere. The reaction

mixture was stirred at room temperature in the dark for 3.5 days. The mixture was then filtered through a pad of Celite and washed with $CHCl_3$ (1000 mL). The solvents were concentrated in vacuum to give compound **1d** as a white solid (3.26 g, 93%). ^{1}H NMR (400 MHz, $CDCl_3$) δ 8.22 (dd, *J* = 7.7, 1.6 Hz, 1H), 8.09 (d, *J* = 8.0 Hz, 1H), 7.97 (td, *J* = 8.5, 7.9, 1.7 Hz, 1H), 7.78 (td, *J* = 7.6, 0.8 Hz, 1H), 2.74 (s, 3H). ^{13}C{^{1}H} NMR (100 MHz, $CDCl_3$) δ 175.0, 161.4, 137.7, 133.8, 133.3, 131.7, 130.1, 115.4, 25.0. FTIR (neat, cm^{-1}): 3293, 3065, 2925, 2855, 1697, 1649, 1576, 1481, 1442, 1359, 1268, 1149, 991, 866, 784, 729, 653, 586.

3. Synthesis of and Characterization Data of β-Monosubstituted Enamines.

General Procedure A[2-3] (R^1 = aryl; R^2 = Me, Et)

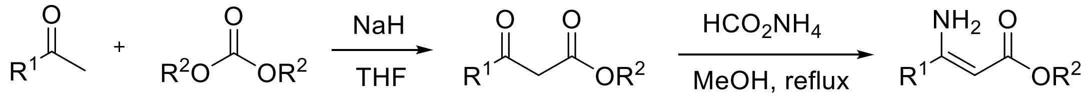

2a-n, 2q-r

Substrates **2a-n** were prepared according to the previously reported procedure[2-3] with some modification. To a solution of ketone (10 mmol, 1.0 equiv) in THF (80 mL) was added methyl dicarbonate (2.5 mL, 30 mmol, 3.0 equiv) and NaH (20 mmol, 60%, 0.8 g, 2.0 equiv). The reaction mixture was refluxed until TLC indicated the total consumption of ketone. After cooling, the reaction mixture was poured into ice-water (80 mL), acidified with aqueous HCl (3.0 M) to pH 2~3 and extracted with EtOAc (100 mL × 3). The combined organic layer was dried over Na_2SO_4 and evaporated under reduced pressure. The obtained ketone methyl ester was further dissolved in absolute methanol (50 mL), followed by the addition of ammonium formate (50 mmol, 3.15 g, 5.0 equiv). The reaction mixture was stirred under reflux for about 5 h and then was filtered through a short pad of Celite. The filtrate was concentrated in vacuum. To the residue was added water (100 mL), then EtOAc (150 mL × 3) was used to extract the mixture and the organic layer was combined, dried over Na_2SO_4 and evaporated to dryness. The desired pure product was obtained by silica gel chromatography using a mixture of PE and EtOAc as eluent. Enamines **2q-r** were prepared under similar enamination approach by using diethyl carbonate as starting material.

General Procedure B[3-4]

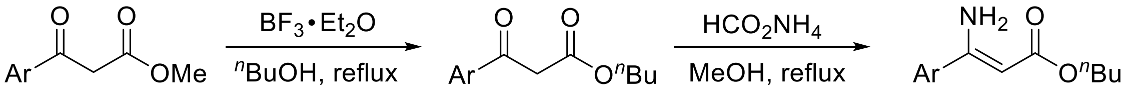

2o-p

Substrates **2o-p** were prepared according to the previously reported procedure[3-4] with some modification. To a solution of ketone methyl ester (10 mmol, 1.0 equiv) in absolute toluene (50 mL) was added $BF_3{\cdot}Et_2O$ and *n*-butyl alcohol (15 mmol, 1.5 mL, 1.5 equiv) in toluene (10 mL). The resulting reaction mixture was refluxed for 6 h. The progress of the reaction was monitored by TLC. After completion, the reaction mixture was cooled and poured into water to remove the catalyst, and the mixture was extracted with CH_2Cl_2 (100 mL × 3). The organic phase was dried with anhydrous Na_2SO_4, and the solvent was removed. The resulting crude product was purified by column chromatography on silica gel and eluted with PE and EtOAc (v/v = 10:1) to afford the corresponding ketone ester, which was further dissolved in absolute methanol (50 mL), followed by the addition of ammonium formate (50 mmol, 3.15 g, 5.0 equiv). The reaction mixture was stirred under reflux for about 5 h and then was filtered through a short pad of Celite. The filtrate was concentrated in vacuum. To the residue was added water (100 mL), then EtOAc (150 mL × 3) was used to extract the mixture and the organic layer was combined, dried over Na_2SO_4 and evaporated to dryness. The desired pure product was obtained by silica gel chromatography using a mixture of PE and EtOAc as eluent.

General Procedure C[2, 5]

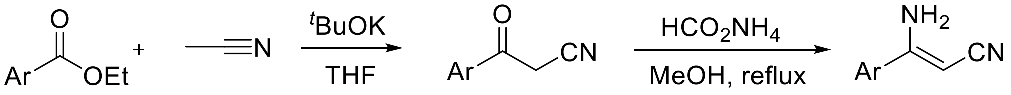

2s-u

Substrates **2s-u** were prepared according to the previously reported procedure.[2, 5] Potassium *tert*-butoxide (24 mmol, 2.7 g, 3.0 equiv) was added to a solution of acetonitrile (8 mmol, 0.4 mL, 1.0 equiv) in anhydrous THF (20 ml) followed by aryl ester (32 mmol, 4.0 equiv). The mixture was stirred at room temperature for 1 h, then diluted with aq. HCl (3.0 M, 50 ml), H_2O (75 ml) and

EtOAc (100 ml). The organic layer was separated, washed with H_2O (100 mL × 2) and brine (100 mL × 2), dried with anhydrous Na_2SO_4, and the solvent was concentrated and the obtained mixture was purified by silica gel chromatography using a mixture of PE and EtOAc as eluent to afford benzoylacetonitrile as colorless oil, which was further dissolved in absolute methanol (50 mL), followed by the addition of ammonium formate (40 mmol, 2.52 g, 5.0 equiv). The reaction mixture was stirred under reflux for about 5 h and then was filtered through a short pad of Celite. The filtrate was concentrated in vacuum. To the residue was added water (100 mL), then EtOAc (150 mL × 3) was used to extract the mixture and the organic layer was combined, dried over Na_2SO_4 and evaporated to dryness. The desired pure product was obtained by silica gel chromatography using a mixture of PE and EtOAc as eluent.

General Procedure D[2-3] (R^1 = aryl, R^2 = aryl, methyl, benzyl)

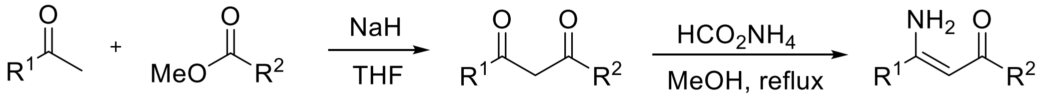

2v-w, **12**

Substrates **2v-w** and **12** were prepared according to the previously reported procedure.[2-3] To a suspension of ketone (10 mmol, 1.0 equiv) in THF (40 mL) was added NaH (20 mmol, 60%, 0.8 g, 2.0 equiv). After the reaction mixture was stirred at 0 °C for about 1 h, ester (12 mmol, 1.2 equiv) was added dropwise at the same temperature. Then the mixture was stirred at room temperature until TLC indicated the total consumption of ketone. The reaction mixture was poured into ice-water (100 mL), acidified with aq. HCl (3.0 M) to pH 2~3 and extracted with EtOAc (100 mL × 3). The combined organic layer was dried over Na_2SO_4 and evaporated under reduced pressure to obtained the corresponding β-diketone, which was further dissolved in absolute methanol (50 mL), followed by the addition of ammonium formate (50 mmol, 3.15 g, 5.0 equiv). The reaction mixture was stirred under reflux for about 5 h and then was filtered through a short pad of Celite. The filtrate was concentrated in vacuum. To the residue was added water (100 mL), then EtOAc (150 mL × 3) was used to extract the mixture and the organic layer was combined, dried over Na_2SO_4 and evaporated to dryness. The desired pure product was obtained by silica gel chromatography using a mixture of PE and EtOAc as eluent.

General Procedure E[6] (R = aryl, naphthyl)

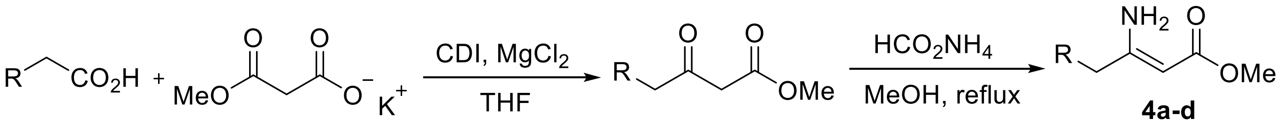

4a-d

Substrates **4a-d** were prepared according to the previously reported procedure.[6] Carboxylic acid (8 mmol, 1.0 equiv) and CDI (8.8 mmol, 1.43 g, 1.1 equiv) were dissolved in anhydrous THF (30 mL) and the mixture was stirred at room temperature for 1 h. Monomethyl potassium malonate (9.6 mmol, 1.5 g, 1.2 equiv) and anhydrous $MgCl_2$ (12 mmol, 1.2 g, 1.5 equiv) were then added in sequence and the mixture was stirred at room temperature overnight. The reaction was treated with aq. HCl (1.0 M, 30 mL). The phases were separated and the aqueous phase was extracted with EtOAc. The combined organic phase was washed with water and brine, dried over Na_2SO_4, filtered and concentrated under reduced pressure. Purification by flash column chromatography using a mixture of PE and EtOAc as eluent to afforded the corresponding β-ketoester, which was further dissolved in absolute methanol (50 mL), followed by the addition of ammonium formate (40 mmol, 2.52 g, 5.0 equiv). The reaction mixture was stirred under reflux for about 48 h and then was filtered through a short pad of Celite. The filtrate was concentrated in vacuum. To the residue was added water (100 mL), then EtOAc (150 mL × 3) was used to extract the mixture and the organic layer was combined, dried over Na_2SO_4 and evaporated to dryness. The desired pure product was obtained by silica gel chromatography using a mixture of PE and EtOAc as eluent.

Procedure F[7]

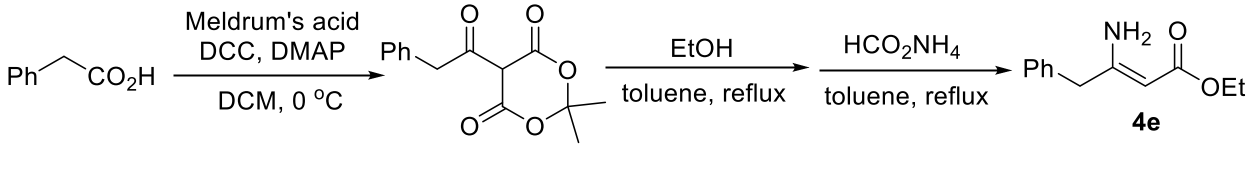

4e

Substrate **4e** was prepared according to the previously reported procedure.[7] 2-Phenylacetic acid (7.35 mmol, 1.0 g, 1.0 equiv), DMAP (8.09 mmol, 0.99 g, 1.1 equiv) and Meldrum's acid (7.35 mmol, 1.06 g, 1.0 equiv) were dissolved in anhydrous DCM (30 mL). Then a solution of DCC (8.09 mmol, 1.7 g, 1.1 equiv) dissolved in anhydrous DCM (15 mL) was added slowly at 0 °C. The

reaction mixture was stirred at 0 °C for about 16 h and then was filtered through a short pad of Celite. The filtrate was washed with aq. $NaHSO_4$ (M = 1) and dried over Na_2SO_4 and evaporated to dryness. To the residue was added alcohol (3 mL) and toluene (25 mL), and the reaction mixture was stirred under reflux for 4 h. Then ammonium formate (29.4 mmol, 1.85 g, 4.0 equiv) was added and the reaction mixture was stirred under reflux for about 6 h and then was filtered through a short pad of Celite. The filtrate was concentrated in vacuum. To the residue was added water (100 mL), then EtOAc (150 mL × 3) was used to extract the mixture and the organic layer was combined, dried over Na_2SO_4 and evaporated to dryness. Ethyl (*Z*)-3-amino-4-phenylbut-2-enoate (**4e**) was obtained by silica gel chromatography using a mixture of PE and EtOAc as eluent.

Procedure G[8]

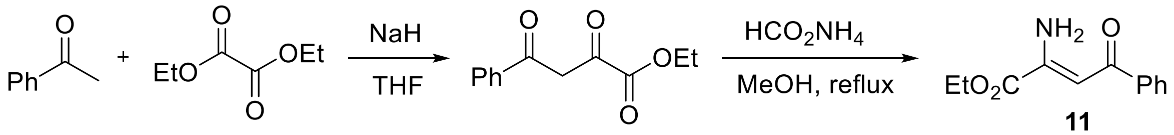

11

Substrate **11** was prepared according to the previously reported procedure.[8] To a suspension of acetophenone (10 mmol, 1.2 mL, 1.0 equiv) in THF (40 mL) was added NaH (20 mmol, 60%, 0.8 g, 2.0 equiv). After the reaction mixture was stirred at 0 °C for about 1 h, diethyl oxalate (12 mmol, 1.63 mL, 1.2 equiv) was added dropwise at the same temperature. Then the mixture was stirred at room temperature until TLC indicated the total consumption of ketone. The reaction mixture was poured into ice-water (100 mL), acidified with aq. HCl (3.0 M) to pH 2~3 and extracted with EtOAc (100 mL × 3). The combined organic layer was dried over Na_2SO_4 and evaporated under reduced pressure to obtained ethyl 2,4-dioxo-4-phenylbutanoate, which was further dissolved in absolute methanol (50 mL), followed by the addition of ammonium formate (50 mmol, 3.15 g, 5.0 equiv). The reaction mixture was stirred under reflux for about 5 h and then was filtered through a short pad of Celite. The filtrate was concentrated in vacuum. To the residue was added water (100 mL), then EtOAc (150 mL × 3) was used to extract the mixture and the organic layer was combined, dried over Na_2SO_4 and evaporated to dryness. Ethyl (*Z*)-2-amino-4-oxo-4-phenylbut-2-enoate (**11**) was obtained by silica gel chromatography using a mixture of PE and EtOAc as eluent.

Enamines **2**, **4**, **11** and **12** are known compounds[2-8] except **2c**, **4a**, **4c-d**.

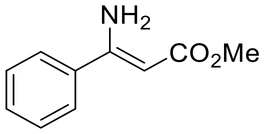

Methyl (*Z*)-3-amino-3-phenylacrylate (2a)

Compound **2a** (1.46 g, 83% yield) was purified with silica gel chromatography (PE/EtOAc = 10:1, R_f = 0.25) as a white solid. ^{1}H NMR (400 MHz, $CDCl_3$) δ 7.53 (dd, *J* = 7.8, 1.6 Hz, 2H), 7.47 – 7.37 (m, 3H), 4.97 (s, 1H), 3.70 (s, 3H). ^{13}C{^{1}H} NMR (100 MHz, $CDCl_3$) δ 170.7, 160.6, 137.5, 130.2, 128.8, 126.1, 84.1, 50.4.

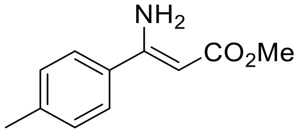

Methyl (*Z*)-3-amino-3-(*p*-tolyl)acrylate (2b)

Compound **2b** (1.55 g, 81% yield) was purified with silica gel chromatography (PE/EtOAc = 10:1, R_f = 0.25) as a white solid. ^{1}H NMR (400 MHz, $CDCl_3$) δ 7.43 (d, *J* = 8.2 Hz, 2H), 7.22 (d, *J* = 7.9 Hz, 2H), 4.96 (s, 1H), 3.71 (s, 3H), 2.38 (s, 3H). ^{13}C{^{1}H} NMR (100 MHz, $CDCl_3$) δ 170.8, 160.6, 140.5, 134.6, 129.5, 126.0, 83.6, 50.4, 21.3.

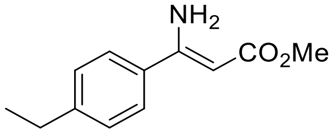

Methyl (*Z*)-3-amino-3-(4-ethylphenyl)acrylate (2c)

Compound **2c** (1.56 g, 76% yield) was purified with silica gel chromatography (PE/EtOAc = 10:1, R_f = 0.25) as a colorless solid. m.p. 36 ~ 38 °C. ^{1}H NMR (400 MHz, $CDCl_3$) δ 7.46 (d, *J* = 8.2 Hz, 2H), 7.24 (d, *J* = 8.2 Hz, 2H), 4.97 (s, 1H), 3.70 (s, 3H), 2.68 (q, *J* = 7.6 Hz, 2H), 1.25 (t, *J* = 7.6 Hz, 3H).

$^{13}C\{^1H\}$ NMR (100 MHz, $CDCl_3$) δ 170.8, 160.6, 146.8, 134.9, 128.3, 126.1, 83.6, 50.3, 28.6, 15.3. HRMS (ESI): Calcd. for $C_{12}H_{16}NO_2$: 206.1176 $(M + H)^+$; Found: 206.2645.

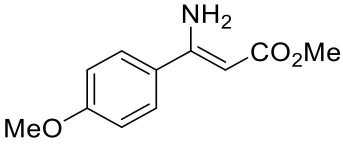

Methyl (*Z*)-3-amino-3-(4-methoxyphenyl)acrylate (2d)

Compound **2d** (1.3 g, 63% yield) was purified with silica gel chromatography (PE/EtOAc = 8:1, R_f = 0.25) as a white solid. ^1H NMR (400 MHz, $CDCl_3$) δ 7.49 (d, J = 8.7 Hz, 2H), 6.92 (d, J = 8.8 Hz, 2H), 4.94 (s, 1H), 3.83 (s, 3H), 3.70 (s, 2H). $^{13}C\{^1H\}$ NMR (100 MHz, $CDCl_3$) δ 170.8, 161.3, 160.3, 129.7, 127.5, 114.1, 83.2, 55.4, 50.4 .

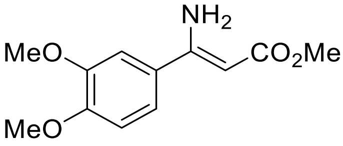

Methyl (*Z*)-3-amino-3-(3,4-dimethoxyphenyl)acrylate (2e)

Compound **2e** (1.33 g, 56% yield) was purified with silica gel chromatography (PE/EtOAc = 5:1, R_f = 0.25) as a white solid. ^1H NMR (400 MHz, $CDCl_3$) δ 7.14 (d, J = 7.7 Hz, 1H), 7.04 (s, 1H), 6.89 (d, J = 8.4 Hz, 1H), 4.94 (s, 1H), 3.91 (s, 6H), 3.71 (s, 3H). $^{13}C\{^1H\}$ NMR (100 MHz, $CDCl_3$) δ 170.6, 160.5, 150.7, 148.9, 130.1, 118.7, 110.9, 109.1, 83.3, 55.9, 50.3.

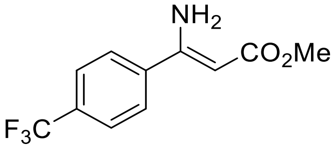

Methyl (*Z*)-3-amino-3-(4-(trifluoromethyl)phenyl)acrylate (2f)

Compound **2f** (1.42 g, 58% yield) was purified with silica gel chromatography (PE/EtOAc = 15:1, R_f = 0.25) as a white solid. ^1H NMR (400 MHz, $CDCl_3$) δ 7.70 – 7.61 (m, 4H), 4.96 (s, 1H), 3.70 (s, 3H). ^{19}F NMR (376 MHz, $CDCl_3$) δ -62.9 (s, 3F). $^{13}C\{^1H\}$ NMR (100 MHz, $CDCl_3$) δ 170.4, 158.9, 141.1, 132.1 (q, J = 32.8 Hz), 126.7, 125.8 (q, J = 3.8 Hz), 123.7 (q, J = 272.4 Hz), 85.5, 50.5.

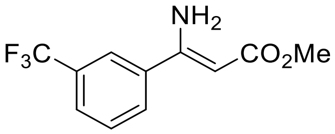

Methyl (*Z*)-3-amino-3-(3-(trifluoromethyl)phenyl)acrylate (2g)

Compound **2g** (1.86 g, 76% yield) was purified with silica gel chromatography (PE/EtOAc = 15:1, R_f = 0.25) as light orange oil. ^{1}H NMR (400 MHz, $CDCl_3$) δ 7.79 (s, 1H), 7.71 (t, *J* = 8.4 Hz, 2H), 7.55 (t, *J* = 7.8 Hz, 1H), 4.97 (s, 1H), 3.72 (s, 3H). ^{19}F NMR (376 MHz, $CDCl_3$) δ -62.9 (s, 3F). ^{13}C{^{1}H} NMR (100 MHz, $CDCl_3$) δ 170.4, 158.8, 138.5, 131.4 (q, *J* = 32.7 Hz), 129.52 (d, *J* = 1.0 Hz), 129.45, 126.9 (q, *J* = 3.7 Hz), 123.7 (q, *J* = 270.0 Hz), 123.2 (q, *J* = 3.8 Hz), 85.5, 50.6.

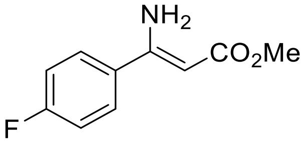

Methyl (*Z*)-3-amino-3-(4-fluorophenyl)acrylate (2h)

Compound **2h** (1.56 g, 80% yield) was purified with silica gel chromatography (PE/EtOAc = 12:1, R_f = 0.25) as a white solid. ^{1}H NMR (400 MHz, $CDCl_3$) δ 7.53 (dd, *J* = 8.8, 5.3 Hz, 2H), 7.10 (t, *J* = 8.6 Hz, 2H), 4.92 (s, 1H), 3.71 (s, 3H). ^{19}F NMR (376 MHz, $CDCl_3$) δ -110.2 – -110.3 (m, 1F). ^{13}C{^{1}H} NMR (100 MHz, $CDCl_3$) δ 170.5, 163.8 (d, *J* = 250.2 Hz), 159.5, 133.6 (d, *J* = 2.0 Hz), 128.1 (d, *J* = 8.5 Hz), 115.8 (d, *J* = 21.8 Hz), 84.3, 50.4.

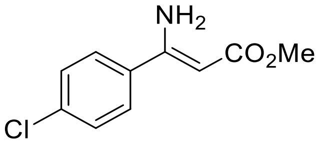

Methyl (*Z*)-3-amino-3-(4-chlorophenyl)acrylate (2i)

Compound **2i** (1.77 g, 84% yield) was purified with silica gel chromatography (PE/EtOAc = 12:1, R_f = 0.25) as a white solid. ^{1}H NMR (400 MHz, $CDCl_3$) δ 7.47 (d, *J* = 8.7 Hz, 2H), 7.38 (d, *J* = 8.6 Hz, 2H), 4.93 (s, 1H), 3.71 (s, 3H). ^{13}C{^{1}H} NMR (100 MHz, $CDCl_3$) δ 170.5, 159.2, 136.2, 136.0, 129.1, 127.5, 84.7, 50.5.

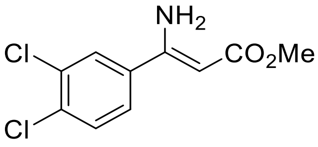

Methyl (*Z*)-3-amino-3-(3,4-dichlorophenyl)acrylate (2j)

Compound **2j** (1.76 g, 72% yield) was purified with silica gel chromatography (PE/EtOAc = 12:1, R_f = 0.25) as a white solid. ^{1}H NMR (400 MHz, $CDCl_3$) δ 7.63 (d, *J* = 2.1 Hz, 1H), 7.49 (d, *J* = 8.4 Hz, 1H), 7.37 (dd, *J* = 8.4, 2.1 Hz, 1H), 4.93 (s, 1H), 3.71 (s, 3H). ^{13}C{^{1}H} NMR (100 MHz, $CDCl_3$) δ 170.4, 157.8, 137.5, 134.4, 133.2, 130.9, 128.2, 125.4, 85.4, 50.6.

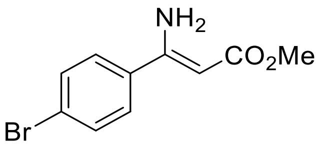

Methyl (*Z*)-3-amino-3-(4-bromophenyl)acrylate (2k)

Compound **2k** (1.56 g, 61% yield) was purified with silica gel chromatography (PE/EtOAc = 15:1, R_f = 0.25) as a white solid. ^{1}H NMR (400 MHz, $CDCl_3$) δ 7.54 (d, *J* = 8.3 Hz, 2H), 7.40 (d, *J* = 8.4 Hz, 2H), 4.93 (s, 1H), 3.70 (s, 3H). ^{13}C{^{1}H} NMR (100 MHz, $CDCl_3$) δ 170.5, 159.3, 136.5, 132.0, 127.7, 124.5, 84.7, 50.5.

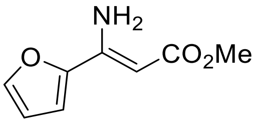

Methyl (*Z*)-3-amino-3-(furan-2-yl)acrylate (2l)

Compound **2l** (1.25 g, 75% yield) was purified with silica gel chromatography (PE/EtOAc = 8:1, R_f = 0.25) as light yellow oil. ^{1}H NMR (400 MHz, $CDCl_3$) δ 7.47 (d, *J* = 0.8 Hz, 1H), 6.74 (d, *J* = 3.5 Hz, 1H), 6.46 (dd, *J* = 3.5, 1.8 Hz, 1H), 5.13 (s, 1H), 3.69 (s, 3H). ^{13}C{^{1}H} NMR (100 MHz, $CDCl_3$) δ 170.8, 149.1, 148.8, 143.7, 112.0, 109.4, 80.8, 50.4.

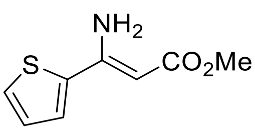

Methyl (*Z*)-3-amino-3-(thiophen-2-yl)acrylate (2m)

Compound **2m** (0.9 g, 49% yield) was purified with silica gel chromatography (PE/EtOAc = 8:1, R_f = 0.25) as a yellow solid. ^{1}H NMR (400 MHz, $CDCl_3$) δ 7.36 (t, *J* = 4.5 Hz, 2H), 7.07 (dd, *J* = 4.9, 3.8 Hz, 1H), 5.11 (s, 1H), 3.70 (s, 3H). ^{13}C{^{1}H} NMR (100 MHz, $CDCl_3$) δ 170.5, 153.1, 139.9, 127.8, 127.3, 125.7, 83.9, 50.5.

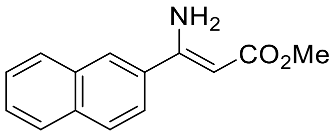

Methyl (*Z*)-3-amino-3-(naphthalen-2-yl)acrylate (2n)

Compound **2n** (1.68 g, 74% yield) was purified with silica gel chromatography (PE/EtOAc = 10:1, R_f = 0.25) as a white solid. ^{1}H NMR (400 MHz, $CDCl_3$) δ 8.05 (d, *J* = 1.1 Hz, 1H), 7.91 – 7.84 (m, 3H), 7.62 (dd, *J* = 8.6, 1.8 Hz, 1H), 7.57 – 7.51 (m, 2H), 5.11 (s, 1H), 3.74 (s, 3H). ^{13}C{^{1}H} NMR (100 MHz, $CDCl_3$) δ 170.7, 160.5, 134.8, 134.1, 133.0, 128.7, 128.5, 127.7, 127.1, 126.8, 125.8, 123.6, 84.8, 50.5.

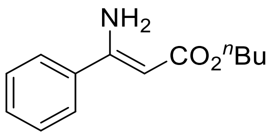

Butyl (*Z*)-3-amino-3-phenylacrylate (2o)

Compound **2o** (1.27 g, 58% yield) was purified with silica gel chromatography (PE/EtOAc = 15:1, R_f = 0.25) as a white solid. ^{1}H NMR (400 MHz, $CDCl_3$) δ 7.54 (dd, *J* = 7.6, 1.7 Hz, 2H), 7.45 – 7.38 (m, 3H), 4.97 (s, 1H), 4.12 (t, *J* = 6.7 Hz, 2H), 1.70 – 1.61 (m, 2H), 1.47 – 1.37 (m, 2H), 0.95 (t, *J* = 7.4 Hz, 3H). ^{13}C{^{1}H} NMR (100 MHz, $CDCl_3$) δ 170.5, 160.4, 137.7, 130.2, 128.8, 126.1, 84.7, 62.9, 31.1, 19.3, 13.8.

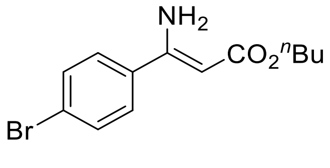

Butyl (*Z*)-3-amino-3-(4-bromophenyl)acrylate (2p)

Compound **2p** (1.51 g, 51% yield) was purified with silica gel chromatography (PE/EtOAc = 15:1, R_f = 0.25) as a white solid. ^{1}H NMR (400 MHz, $CDCl_3$) δ 7.54 (dd, *J* = 8.5, 1.9 Hz, 2H), 7.41 (dd, *J* = 8.4, 1.6 Hz, 2H), 4.93 (s, 1H), 4.11 (td, *J* = 6.7, 1.5 Hz, 2H), 1.69 – 1.59 (m, 2H), 1.47 – 1.35 (m, 2H), 0.94 (t, *J* = 7.4 Hz, 3H). ^{13}C{^{1}H} NMR (100 MHz, $CDCl_3$) δ 170.3, 159.1, 136.6, 132.0, 127.7, 124.4, 85.2, 63.0, 31.0, 19.2, 13.8.

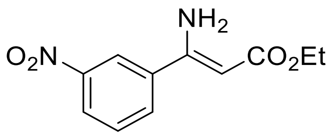

Ethyl (*Z*)-3-amino-3-(3-nitrophenyl)acrylate (2q)

Compound **2q** (1.75 g, 74% yield) was purified with silica gel chromatography (PE/EtOAc = 8:1, R_f = 0.25) as a yellow solid. ^{1}H NMR (400 MHz, $CDCl_3$) δ 8.40 (t, *J* = 1.9 Hz, 1H), 8.28 (ddd, *J* = 8.2, 2.2, 1.0 Hz, 1H), 7.89 – 7.85 (m, 1H), 7.61 (t, *J* = 8.0 Hz, 1H), 5.00 (s, 1H), 4.18 (q, *J* = 7.1 Hz, 2H), 1.30 (t, *J* = 7.1 Hz, 3H). ^{13}C{^{1}H} NMR (100 MHz, $CDCl_3$) δ 169.9, 157.4, 148.4, 139.4, 132.1, 123.0, 124.8, 121.3, 86.6, 59.3, 14.5.

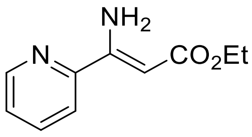

Ethyl (*Z*)-3-amino-3-(pyridin-2-yl)acrylate (2r)

Compound **2r** (1.25 g, 65% yield) was purified with silica gel chromatography (PE/EtOAc = 5:1, R_f = 0.25) as a brown solid. ^{1}H NMR (400 MHz, $CDCl_3$) δ 8.63 (d, *J* = 4.8 Hz, 1H), 7.77 – 7.71 (m, 2H), 7.36 – 7.32 (m, 1H), 5.34 (s, 1H), 4.20 (q, *J* = 7.1 Hz, 2H), 1.31 (t, *J* = 7.1 Hz, 3H). ^{13}C{^{1}H} NMR (100 MHz, $CDCl_3$) δ 170.6, 155.4, 151.2, 148.7, 136.7, 124.6, 120.1, 82.2, 58.9, 14.5.

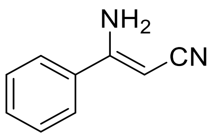

(*Z*)-3-Amino-3-phenylacrylonitrile (2s)

Compound **2s** (1.05 g, 91% yield) was purified with silica gel chromatography (PE/EtOAc = 5:1, R_f = 0.25) as an orange solid. ^{1}H NMR (400 MHz, $CDCl_3$) δ 7.51 – 7.40 (m, 5H), 4.96 (s, 2H), 4.24 (s, 1H). $^{13}C\{^{1}H\}$ NMR (100 MHz, $CDCl_3$) δ 161.5, 135.3, 130.9, 129.0, 126.0, 119.5, 63.7.

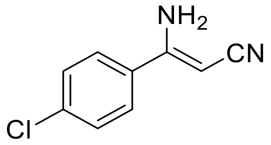

(*Z*)-3-Amino-3-(4-chlorophenyl)acrylonitrile (2t)

Compound **2t** (1.08 g, 76% yield) was purified with silica gel chromatography (PE/EtOAc = 5:1, R_f = 0.25) as a yellow solid. ^{1}H NMR (400 MHz, $CDCl_3$) δ 7.44 (d, *J* = 8.8 Hz, 2H), 6.92 (d, *J* = 8.8 Hz, 2H), 4.91 (s, 2H), 4.17 (s, 1H), 3.83 (s, 3H). $^{13}C\{^{1}H\}$ NMR (100 MHz, $CDCl_3$) δ 161.7, 161.1, 127.5, 127.4, 119.9, 114.2, 62.4, 55.4.

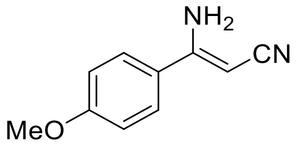

(*Z*)-3-Amino-3-(4-methoxyphenyl)acrylonitrile (2u)

Compound **2u** (0.93 g, 67% yield) was purified with silica gel chromatography (PE/EtOAc = 3:1, R_f = 0.25) as a yellow solid. ^{1}H NMR (400 MHz, $CDCl_3$) δ 7.46 – 7.37 (m, 4H), 4.93 (s, 2H), 4.22 (s, 1H). $^{13}C\{^{1}H\}$ NMR (100 MHz, $CDCl_3$) δ 160.2, 137.0, 133.7, 129.2, 127.3, 119.1, 64.4.

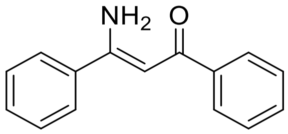

(*Z*)-3-Amino-1,3-diphenylprop-2-en-1-one (2v)

Compound **2v** (2.05 g, 92% yield) was purified with silica gel chromatography (PE/EtOAc = 2:1, R_f = 0.25) as a white solid. ^{1}H NMR (400 MHz, $CDCl_3$) δ 10.43 (br, 1H), 7.95 (dd, *J* = 7.9, 1.5 Hz, 2H),

7.65 (dd, J = 7.8, 1.7 Hz, 2H), 7.52 – 7.41 (m, 6H), 6.16 (s, 1H), 5.45 (br, 1H). $^{13}C\{^1H\}$ NMR (100 MHz, $CDCl_3$) δ 190.1, 162.9, 140.3, 137.6, 131.0, 130.7, 129.0, 128.3, 127.2, 126.3, 91.9.

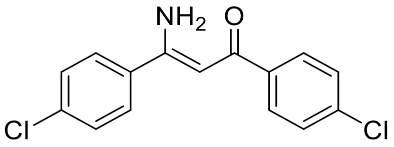

(*Z*)-3-Amino-1,3-bis(4-chlorophenyl)prop-2-en-1-one (2w)

Compound **2w** (2.39 g, 82% yield) was purified with silica gel chromatography (PE/EtOAc = 4:1, R_f = 0.25) as a white solid. ^1H NMR (400 MHz, $CDCl_3$) δ 10.37 (br, 1H), 7.86 (d, J = 8.3 Hz, 2H), 7.56 (d, J = 8.3 Hz, 2H), 7.42 (dd, J = 18.4, 8.4 Hz, 4H), 6.04 (s, 1H), 5.48 (br, 1H). $^{13}C\{^1H\}$ NMR (100 MHz, $CDCl_3$) δ 188.8, 162.0, 138.4, 137.4, 137.0, 135.8, 129.3, 128.6, 128.6, 127.7, 91.7.

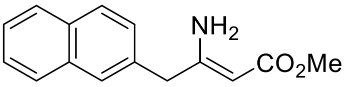

Methyl (*Z*)-3-amino-4-(naphthalen-2-yl)but-2-enoate (4a)

Compound **4a** (1.7 g, 88% yield) was purified with silica gel chromatography (PE/EtOAc = 10:1, R_f = 0.25) as a yellow solid. m.p. 53 ~ 56 °C. ^1H NMR (400 MHz, $CDCl_3$) δ 7.85 – 7.78 (m, 3H), 7.69 (s, 1H), 7.53 – 7.44 (m, 2H), 7.34 (dd, J = 8.4, 1.7 Hz, 1H), 4.71 (s, 1H), 3.66 (s, 3H), 3.63 (s, 2H), 2.09 (s, 1H). $^{13}C\{^1H\}$ NMR (100 MHz, $CDCl_3$) δ 170.6, 161.4, 133.6, 133.4, 132.5, 128.6, 127.8, 127.7, 127.5, 126.9, 126.4, 125.9, 84.7, 50.2, 42.4. HRMS (ESI): Calcd. for $C_{15}H_{16}NO_2$: 242.1176 $(M + H)^+$; Found: 242.1186.

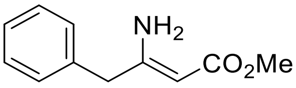

Methyl (*Z*)-3-amino-4-phenylbut-2-enoate (4b)

Compound **4b** (1.2 g, 77% yield) was purified with silica gel chromatography (PE/EtOAc = 10:1, R_f = 0.25) as colorless oil. ^1H NMR (400 MHz, $CDCl_3$) δ 7.35 – 7.21 (m, 5H), 4.64 (s, 1H), 3.64 (s, 3H), 3.45 (s, 2H). $^{13}C\{^1H\}$ NMR (100 MHz, $CDCl_3$) δ 170.4, 161.6, 136.1, 128.9, 128.7, 127.1, 84.4, 50.1, 42.1, 29.6.

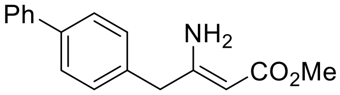

Methyl (*Z*)-4-([1,1'-biphenyl]-4-yl)-3-aminobut-2-enoate (4c)

Compound **4c** (1.22 g, 57% yield) was purified with silica gel chromatography (PE/EtOAc = 10:1, R_f = 0.25) as a white solid. m.p. 99 ~ 101 °C. ^{1}H NMR (400 MHz, $CDCl_3$) δ 7.57 (t, *J* = 7.6 Hz, 4H), 7.44 (t, *J* = 7.5 Hz, 2H), 7.35 (t, *J* = 7.3 Hz, 1H), 7.31 (d, *J* = 8.2 Hz, 2H), 4.68 (s, 1H), 3.67 (s, 3H), 3.51 (s, 2H). $^{13}C\{^1H\}$ NMR (100 MHz, $CDCl_3$) δ 170.5, 140.6, 140.3, 135.1, 129.5, 128.8, 127.5, 127.4, 127.0, 84.7, 50.2, 41.9. HRMS (ESI): Calcd. for $C_{17}H_{18}NO_2$: 268.1332 $(M + H)^+$; Found: 268.1343.

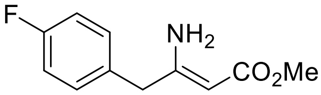

Methyl (*Z*)-3-amino-4-(4-fluorophenyl)but-2-enoate (4d)

Compound **4d** (1.25 g, 75% yield) was purified with silica gel chromatography (PE/EtOAc = 10:1, R_f = 0.25) as light yellow oil. ^{1}H NMR (400 MHz, $CDCl_3$) δ 7.19 (dd, *J* = 8.5, 5.5 Hz, 2H), 7.01 (t, *J* = 8.6 Hz, 2H), 4.60 (s, 1H), 3.64 (s, 3H), 3.43 (s, 2H). ^{19}F NMR (376 MHz, $CDCl_3$) δ -115.3 – -115.4 (m, 1F). $^{13}C\{^1H\}$ NMR (100 MHz, $CDCl_3$) δ 170.5, 162.0 (d, *J* = 245.0 Hz), 161.3, 131.8 (d, *J* = 3.1 Hz), 130.5 (d, *J* = 8.0 Hz), 115.6 (d, *J* = 21.4 Hz), 84.7, 50.2, 41.4. HRMS (ESI): Calcd. for $C_{11}H_{13}FNO_2$: 210.0925 $(M + H)^+$; Found: 210.0934.

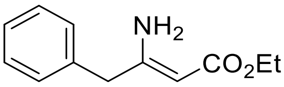

Ethyl (*Z*)-3-amino-4-phenylbut-2-enoate (4e)

Compound **4e** (1.44 g, 88% yield) was purified with silica gel chromatography (PE/EtOAc = 10:1, R_f = 0.25) as a white solid. ^{1}H NMR (400 MHz, $CDCl_3$) δ 7.34 – 7.21 (m, 5H), 4.62 (s, 1H), 4.10 (q, *J* = 7.1 Hz, 2H), 3.44 (s, 2H), 1.25 (t, *J* = 7.1 Hz, 3H). $^{13}C\{^1H\}$ NMR (100 MHz, $CDCl_3$) δ 170.1, 161.4, 136.1, 129.0, 128.7, 127.1, 84.8, 58.6, 42.2, 14.5.

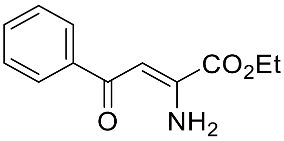

Ethyl (*Z*)-2-amino-4-oxo-4-phenylbut-2-enoate (11)

Compound **11** (1.92 g, 88% yield) was purified with silica gel chromatography (PE/EtOAc = 2:1, R_f = 0.25) as a colorless solid. ^{1}H NMR (400 MHz, $CDCl_3$) δ 9.45 (br, 1H), 7.95 (d, *J* = 8.1 Hz, 2H), 7.54 – 7.40 (m, 3H), 6.65 (s, 1H), 6.04 (br, 1H), 4.38 (q, *J* = 7.1 Hz, 2H), 1.39 (t, *J* = 7.1 Hz, 3H). ^{13}C{^{1}H} NMR (100 MHz, $CDCl_3$) δ 191.8, 163.9, 147.5, 139.3, 131.8, 128.4, 127.4, 93.2, 62.7, 14.1.

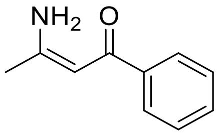

(*Z*)-3-Amino-1-phenylbut-2-en-1-one (12)

Compound **12** (1.38 g, 86% yield) was purified with silica gel chromatography (PE/EtOAc = 2:1, R_f = 0.25) as a white solid. ^{1}H NMR (400 MHz, $CDCl_3$) δ 10.19 (br, 1H), 7.87 (dd, *J* = 8.0, 1.6 Hz, 2H), 7.48 – 7.36 (m, 3H), 5.72 (s, 1H), 5.43 (br, 1H), 2.03 (s, 3H). ^{13}C{^{1}H} NMR (100 MHz, $CDCl_3$) δ 189.4, 163.2, 140.1, 130.7, 128.2, 127.0, 92.2, 22.8.

4. General Procedure and Characterization Data of Compounds 3.

To a 20 mL of Schlenk tube equipped with a stirrer was added β-monosubstituted enamine **2** (0.3 mmol, 1.0 equiv), O_2NO-I(III) **1d** (0.45 mmol, 158 mg, 1.5 equiv) and CuI (0.03 mmol, 6 mg, 10 mol%) under N_2 atmosphere, followed by addition of acetonitrile (4 mL). The tube was screw-capped and stirred at 60 °C. After stirring for 6 h, the reaction mixture was diluted with dichloromethane, filtered through a pad of Celite and concentrated in vacuum. The residue was purified with silica gel chromatography (PE/EtOAc) to afford **3**.

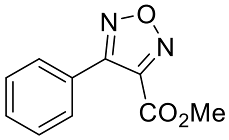

Methyl 4-phenyl-1,2,5-oxadiazole-3-carboxylate (3a)

Compound **3a** (49 mg, 80% yield) was purified with silica gel chromatography (PE/EtOAc = 25:1, R_f = 0.20) as light yellow oil. ^{1}H NMR (400 MHz, $CDCl_3$) δ 7.81 (d, *J* = 7.0 Hz, 2H), 7.60 – 7.47 (m, 3H), 4.02 (s, 3H). ^{13}C{^{1}H} NMR (100 MHz, $CDCl_3$) δ 159.1, 154.4, 146.0, 131.1, 129.3, 128.7,

124.2, 53.5. HRMS (ESI): Calcd. for $C_{10}H_9N_2O_3$: 205.0608 $(M + H)^+$; Found: 205.0619.

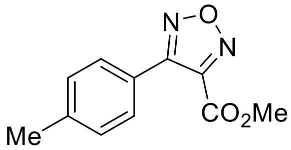

Methyl 4-(*p*-tolyl)-1,2,5-oxadiazole-3-carboxylate (3b)

Compound **3b** (58 mg, 88% yield) was purified with silica gel chromatography (PE/EtOAc = 25:1, R_f = 0.20) as a white solid. m.p. 52 ~ 55 °C. ^{1}H NMR (400 MHz, $CDCl_3$) δ 7.70 (d, *J* = 8.2 Hz, 2H), 7.31 (d, *J* = 8.0 Hz, 2H), 4.01 (s, 3H), 2.43 (s, 3H). $^{13}C\{^1H\}$ NMR (100 MHz, $CDCl_3$) δ 159.1, 154.3, 146.0, 141.4, 129.4, 129.2, 121.2, 53.4, 21.5. HRMS (ESI): Calcd. for $C_{11}H_{10}N_2O_3Na$: 241.0584 $(M + Na)^+$; Found: 241.0575.

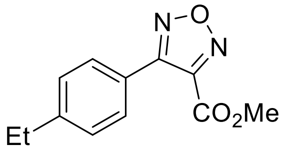

Methyl 4-(4-ethylphenyl)-1,2,5-oxadiazole-3-carboxylate (3c)

Compound **3c** (56 mg, 81% yield) was purified with silica gel chromatography (PE/EtOAc = 25:1, R_f = 0.20) as a white solid. m.p. 57 ~ 60 °C. ^{1}H NMR (400 MHz, $CDCl_3$) δ 7.73 (d, *J* = 8.2 Hz, 2H), 7.34 (d, *J* = 8.2 Hz, 2H), 4.02 (s, 3H), 2.73 (q, *J* = 7.6 Hz, 2H), 1.28 (t, *J* = 7.6 Hz, 3H). $^{13}C\{^1H\}$ NMR (100 MHz, $CDCl_3$) δ 159.2, 154.3, 147.7, 146.0, 129.3, 128.3, 121.4, 53.5, 28.8, 15.2. HRMS (ESI): Calcd. for $C_{12}H_{13}N_2O_3$: 233.0921 $(M + H)^+$; Found: 233.0913.

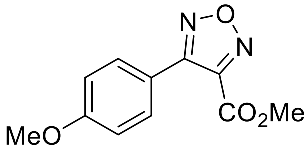

Methyl 4-(4-methoxyphenyl)-1,2,5-oxadiazole-3-carboxylate (3d)

Compound **3d** (60 mg, 86% yield) was purified with silica gel chromatography (PE/EtOAc = 10:1, R_f = 0.20) as a white solid. m.p. 79 ~ 81 °C. ^{1}H NMR (400 MHz, $CDCl_3$) δ 7.48 7.78 (d, *J* = 8.7 Hz, 2H), 7.00 (d, *J* = 8.7 Hz, 2H), 4.01 (s, 3H), 3.86 (s, 3H). $^{13}C\{^1H\}$ NMR (100 MHz, $CDCl_3$) δ 161.7, 159.2, 153.9, 145.8, 130.8, 116.3, 114.2, 55.3, 53.4. HRMS (ESI): Calcd. for $C_{11}H_{10}N_2O_4Na$: 257.0533 $(M + Na)^+$; Found: 257.0521.

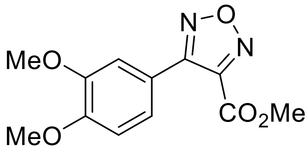

Methyl 4-(3,4-dimethoxyphenyl)-1,2,5-oxadiazole-3-carboxylate (3e)

Compound **3e** (62 mg, 78% yield) was purified with silica gel chromatography (PE/EtOAc = 4:1, R_f = 0.20) as a white solid. m.p. 112 ~ 115 °C. ^{1}H NMR (400 MHz, $CDCl_3$) δ 7.48 – 7.40 (m, 2H), 6.95 (d, J = 8.3 Hz, 1H), 4.00 (s, 3H), 3.92 (s, 6H). ^{13}C{^{1}H} NMR (100 MHz, $CDCl_3$) δ 159.3, 153.9, 151.3, 149.0, 145.8, 122.6, 116.4, 112.1, 110.9, 56.0, 55.9, 53.5. HRMS (ESI): Calcd. for $C_{12}H_{12}N_2O_5Na$: 287.0638 $(M + Na)^+$; Found: 287.0629.

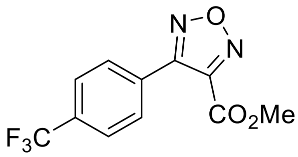

Methyl 4-(4-(trifluoromethyl)phenyl)-1,2,5-oxadiazole-3-carboxylate (3f)

Compound **3f** (51 mg, 63% yield) was purified with silica gel chromatography (PE/EtOAc = 30:1, R_f = 0.20) as a white solid. m.p. 40 ~ 42 °C. ^{1}H NMR (400 MHz, $CDCl_3$) δ 7.97 (d, J = 8.1 Hz, 2H), 7.78 (d, J = 8.2 Hz, 2H), 4.03 (s, 3H). ^{19}F NMR (376 MHz, $CDCl_3$) δ -63.13 (s, 3F). ^{13}C{^{1}H} NMR (100 MHz, $CDCl_3$) δ 158.8, 153.5, 145.9, 133.0 (q, J = 33.0 Hz), 129.9, 127.8, 125.7 (q, J = 3.3 Hz), 123.6 (q, J = 271.0 Hz), 53.6. HRMS (ESI): Calcd. for $C_{11}H_7F_3N_2O_3Na$: 295.0301 $(M + Na)^+$; Found: 295.0333.

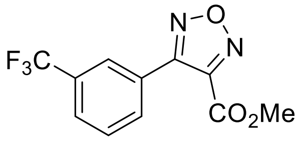

Methyl 4-(3-(trifluoromethyl)phenyl)-1,2,5-oxadiazole-3-carboxylate (3g)

Compound **3g** (61 mg, 75% yield) was purified with silica gel chromatography (PE/EtOAc = 30:1, R_f = 0.20) as a white solid. m.p. 41 ~ 43 °C. ^{1}H NMR (400 MHz, $CDCl_3$) δ 8.13 (s, 1H), 8.04 (d, J = 7.8 Hz, 1H), 7.82 (d, J = 7.9 Hz, 1H), 7.66 (t, J = 7.8 Hz, 1H), 4.03 (s, 3H). ^{19}F NMR (376 MHz, $CDCl_3$) δ -62.92 (s, 3F). ^{13}C{^{1}H} NMR (100 MHz, $CDCl_3$) δ 158.8, 153.4, 145.9, 132.7, 131.4 (q, J = 32.7 Hz), 129.3, 127.8 (q, J = 3.7 Hz), 126.5 (q, J = 3.7 Hz), 125.2, 123.6 (q, J = 271.0 Hz), 53.6.

HRMS (ESI): Calcd. For $C_{11}H_7F_3N_2O_3Na$: 295.0301 $(M + Na)^+$; Found: 295.0297.

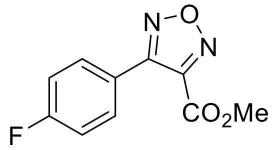

Methyl 4-(4-fluorophenyl)-1,2,5-oxadiazole-3-carboxylate (3h)

Compound **3h** (46 mg, 69% yield) was purified with silica gel chromatography (PE/EtOAc = 25:1, R_f = 0.20) as a yellow solid. m.p. 49 ~ 52 °C. ^{1}H NMR (400 MHz, $CDCl_3$) δ 7.87 – 7.80 (m, 2H), 7.18 (t, J = 8.6 Hz, 2H), 4.00 (s, 3H). ^{19}F NMR (376 MHz, $CDCl_3$) δ -108.40 – -108.54 (m, 1F). ^{13}C{^{1}H} NMR (100 MHz, $CDCl_3$) δ 164.3 (d, J = 250.0 Hz), 159.0, 153.5, 145.8, 131.6 (d, J = 9.0 Hz), 120.3 (d, J = 3.0 Hz), 115.9 (d, J = 22.0 Hz), 53.5. HRMS (ESI): Calcd. for $C_{10}H_7FN_2O_3Na$: 245.0333 $(M + Na)^+$; Found: 245.0325.

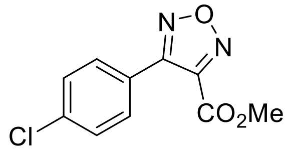

Methyl 4-(4-chlorophenyl)-1,2,5-oxadiazole-3-carboxylate (3i)

Compound **3i** (51 mg, 71% yield) was purified with silica gel chromatography (PE/EtOAc = 25:1, R_f = 0.20) as a yellow solid. m.p. 70 ~ 72 °C. ^{1}H NMR (400 MHz, $CDCl_3$) δ 7.79 (d, J = 8.6 Hz, 2H), 7.49 (d, J = 8.6 Hz, 2H), 4.02 (s, 3H). ^{13}C{^{1}H} NMR (100 MHz, $CDCl_3$) δ 159.0, 153.6, 145.8, 137.5, 130.7, 129.1, 122.7, 53.6. HRMS (ESI): Calcd. for $C_{10}H_7N_2O_3ClNa$: 261.0037 $(M + Na)^+$; Found: 261.0026.

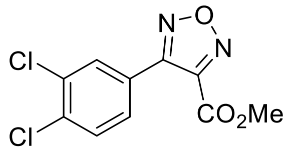

Methyl 4-(3,4-dichlorophenyl)-1,2,5-oxadiazole-3-carboxylate (3j)

Compound **3j** (51 mg, 62% yield) was purified with silica gel chromatography (PE/EtOAc = 30:1, R_f = 0.20) as a white solid. m.p. 103 ~ 105 °C. ^{1}H NMR (400 MHz, $CDCl_3$) δ 7.99 (d, J = 2.0 Hz, 1H), 7.71 (dd, J = 8.4, 2.1 Hz, 1H), 7.59 (d, J = 8.4 Hz, 1H), 4.04 (s, 3H). ^{13}C{^{1}H} NMR (100 MHz, $CDCl_3$) δ 158.8, 152.7, 145.7, 135.8, 133.2, 131.3, 130.8, 128.6, 124.1, 53.7. HRMS (ESI): Calcd.

for $C_{10}H_7N_2O_3Cl_2$: 272.9828 $(M + H)^+$; Found: 272.9846.

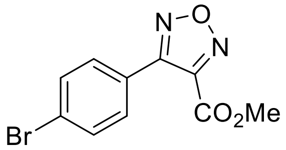

Methyl 4-(4-bromophenyl)-1,2,5-oxadiazole-3-carboxylate (3k)

Compound **3k** (68 mg, 80% yield) was purified with silica gel chromatography (PE/EtOAc = 30:1, R_f = 0.20) as a yellow solid. m.p. 81 ~ 84 °C. ^{1}H NMR (400 MHz, $CDCl_3$) δ 7.71 (d, J = 8.5 Hz, 2H), 7.65 (d, J = 8.3 Hz, 2H), 4.02 (s, 3H). ^{13}C{^{1}H} NMR (100 MHz, $CDCl_3$) δ 158.9, 153.6, 145.8, 132.1, 130.9, 125.9, 123.1, 53.6. HRMS (ESI): Calcd. for $C_{10}H_7N_2O_3BrNa$: 304.9532 $(M + Na)^+$; Found: 304.9533.

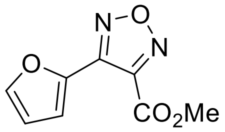

Methyl 4-(furan-2-yl)-1,2,5-oxadiazole-3-carboxylate (3l)

Compound **3l** (27 mg, 47% yield) was purified with silica gel chromatography (PE/EtOAc = 20:1, R_f = 0.20) as a white solid. m.p. 67 ~ 70 °C. ^{1}H NMR (400 MHz, $CDCl_3$) δ 7.67 (dd, J = 1.7, 0.6 Hz, 1H), 7.56 (dd, J = 3.6, 0.6 Hz, 1H), 6.60 (dd, J = 3.6, 1.8 Hz, 1H), 4.07 (s, 3H). ^{13}C{^{1}H} NMR (100 MHz, $CDCl_3$) δ 158.7, 145.9, 145.8, 144.6, 139.4, 116.9, 112.1, 53.6. HRMS (ESI): Calcd. for $C_8H_6N_2O_4Na$: 217.0220 $(M + Na)^+$; Found: 217.0222.

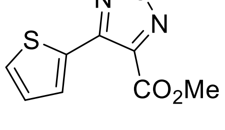

Methyl 4-(thiophen-2-yl)-1,2,5-oxadiazole-3-carboxylate (3m)

Compound **3m** (34 mg, 54% yield) was purified with silica gel chromatography (PE/EtOAc = 20:1, R_f = 0.20) as light yellow oil. ^{1}H NMR (400 MHz, $CDCl_3$) δ 8.06 (d, J = 3.8 Hz, 1H), 7.57 (d, J = 5.1 Hz, 1H), 7.18 (t, J = 4.4 Hz, 1H), 4.07 (s, 3H). ^{13}C{^{1}H} NMR (100 MHz, $CDCl_3$) δ 159.0, 149.3, 144.9, 132.2, 130.3, 128.0, 124.5, 53.6. HRMS (ESI): Calcd. for $C_8H_7N_2O_3S$: 211.0172 $(M + H)^+$; Found: 211.0176.

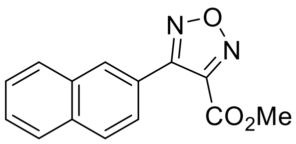

Methyl 4-(naphthalen-2-yl)-1,2,5-oxadiazole-3-carboxylate (3n)

Compound **3n** (60 mg, 79% yield) was purified with silica gel chromatography (PE/EtOAc = 25:1, R_f = 0.20) as a white solid. m.p. 69 ~ 72 °C. ^{1}H NMR (400 MHz, $CDCl_3$) δ 8.39 (s, 1H), 7.95 (t, *J* = 8.2 Hz, 2H), 7.90 (d, *J* = 7.6 Hz, 1H), 7.85 (dd, *J* = 8.6, 1.7 Hz, 1H), 7.67 – 7.47 (m, 2H), 4.03 (s, 3H). ^{13}C{^{1}H} NMR (100 MHz, $CDCl_3$) δ 159.1, 154.4, 146.1, 134.2, 132.7, 129.8, 128.7, 128.5, 127.8 (d, *J* = 1.0 Hz), 126.9, 125.6, 121.5, 53.5. HRMS (ESI): Calcd. for $C_{14}H_{10}N_2O_3Na$: 277.0584 $(M + Na)^+$; Found: 277.0597.

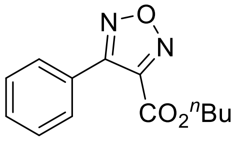

Butyl 4-phenyl-1,2,5-oxadiazole-3-carboxylate (3o)

Compound **3o** (52 mg, 70% yield) was purified with silica gel chromatography (PE/EtOAc = 25:1, R_f = 0.20) as colorless oil. ^{1}H NMR (400 MHz, $CDCl_3$) δ 7.81 – 7.76 (m, 2H), 7.56 – 7.47 (m, 3H), 4.41 (t, *J* = 6.7 Hz, 2H), 1.78 – 1.69 (m, 2H), 1.45 – 1.35 (m, 2H), 0.94 (t, *J* = 7.4 Hz, 3H). ^{13}C{^{1}H} NMR (100 MHz, $CDCl_3$) δ 158.7, 154.3, 146.3, 131.0, 129.3, 128.7, 124.4, 66.9, 30.3, 19.0, 13.6. HRMS (ESI): Calcd. for $C_{13}H_{15}N_2O_3$: 247.1077 $(M + H)^+$; Found: 247.1080.

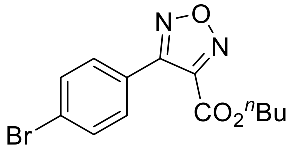

Butyl 4-(4-bromophenyl)-1,2,5-oxadiazole-3-carboxylate (3p)

Compound **3p** (72 mg, 74% yield) was purified with silica gel chromatography (PE/EtOAc = 30:1, R_f = 0.20) as colorless oil. ^{1}H NMR (400 MHz, $CDCl_3$) δ 7.70 (d, *J* = 8.6 Hz, 2H), 7.64 (d, *J* = 8.6 Hz, 2H), 4.41 (t, *J* = 6.7 Hz, 2H), 1.79 – 1.70 (m, 2H), 1.48 – 1.35 (m, 2H), 0.95 (t, *J* = 7.4 Hz, 3H). ^{13}C{^{1}H} NMR (100 MHz, $CDCl_3$) δ 158.6, 153.6, 146.1, 132.0, 130.9, 125.8, 123.3, 67.0, 30.3, 19.0, 13.6. HRMS (ESI): Calcd. for $C_{13}H_{13}N_2O_3BrK$: 362.9741 $(M + K)^+$; Found: 362.9732.

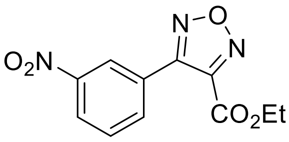

Ethyl 4-(3-nitrophenyl)-1,2,5-oxadiazole-3-carboxylate (3q)

Compound **3q** (57 mg, 72% yield) was purified with silica gel chromatography (PE/EtOAc = 8:1, R_f = 0.20) as light yellow oil. ^{1}H NMR (400 MHz, $CDCl_3$) δ 8.76 (t, *J* = 1.9 Hz, 1H), 8.41 (ddd, *J* = 8.3, 2.2, 1.0 Hz, 1H), 8.20 (dt, *J* = 7.8, 1.2 Hz, 1H), 7.73 (t, *J* = 8.0 Hz, 1H), 4.51 (q, *J* = 7.1 Hz, 2H), 1.43 (t, *J* = 7.1 Hz, 3H). $^{13}C\{^1H\}$ NMR (100 MHz, $CDCl_3$) δ 158.2, 152.8, 148.2, 146.0, 135.3, 129.9, 126.1, 125.7, 124.7, 63.5, 13.9. HRMS (ESI): Calcd. for $C_{11}H_9N_3O_5Na$: 286.0434 $(M + Na)^+$; Found: 286.0446.

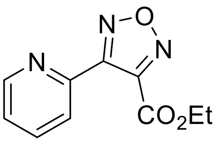

Ethyl 4-(pyridin-2-yl)-1,2,5-oxadiazole-3-carboxylate (3r)

Compound **3r** (25 mg, 38% yield) was purified with silica gel chromatography (PE/EtOAc = 10:1, R_f = 0.20) as a yellow solid. m.p. 40 ~ 42 ^{o}C. ^{1}H NMR (400 MHz, $CDCl_3$) δ 8.69 (d, *J* = 4.6 Hz, 1H), 8.00 (d, *J* = 7.9 Hz, 1H), 7.87 (td, *J* = 7.8, 1.7 Hz, 1H), 7.44 (ddd, *J* = 7.6, 4.9, 1.1 Hz, 1H), 4.50 (d, *J* = 7.1 Hz, 2H), 1.40 (t, *J* = 7.1 Hz, 3H). $^{13}C\{^1H\}$ NMR (100 MHz, $CDCl_3$) δ 159.0, 153.3, 149.9, 147.3, 144.6, 137.1, 125.3, 123.8, 63.1, 13.9. HRMS (ESI): Calcd. for $C_{10}H_{10}N_3O_3$: 220.0717 $(M + H)^+$; Found: 220.0720.

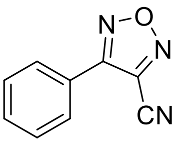

4-Phenyl-1,2,5-oxadiazole-3-carbonitrile (3s)

Compound **3s** (29 mg, 56% yield) was purified with silica gel chromatography (PE/EtOAc = 20:1, R_f = 0.20) as light yellow oil. ^{1}H NMR (400 MHz, $CDCl_3$) δ 8.05 – 7.98 (m, 2H), 7.67 – 7.53 (m, 3H). $^{13}C\{^1H\}$ NMR (100 MHz, $CDCl_3$) δ 154.5, 132.5, 130.6, 129.7, 127.8, 122.6, 108.3. HRMS (ESI): Calcd. for $C_9H_6N_3O$: 172.0505 $(M + H)^+$; Found: 172.0513.

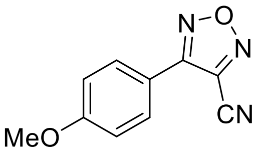

4-(4-Methoxyphenyl)-1,2,5-oxadiazole-3-carbonitrile (3t)

Compound **3t** (37 mg, 61% yield) was purified with silica gel chromatography (PE/EtOAc = 15:1, R_f = 0.20) as a white solid. m.p. 54 ~ 56 °C. ^{1}H NMR (400 MHz, $CDCl_3$) δ 7.97 (d, *J* = 8.9 Hz, 2H), 7.08 (d, *J* = 8.9 Hz, 2H), 3.90 (s, 3H). ^{13}C{^{1}H} NMR (100 MHz, $CDCl_3$) δ 162.8, 154.1, 130.3, 129.4, 115.2, 114.7, 108.6, 55.5. HRMS (ESI): Calcd. for $C_{10}H_8N_3O_2$: 202.0611 $(M + H)^+$; Found: 202.0617.

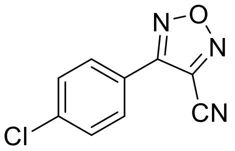

4-(4-Chlorophenyl)-1,2,5-oxadiazole-3-carbonitrile (3u)

Compound **3u** (33 mg, 54% yield) was purified with silica gel chromatography (PE/EtOAc = 20:1, R_f = 0.20) as a white solid. m.p. 52 ~ 55 °C. ^{1}H NMR (400 MHz, $CDCl_3$) δ 7.97 (d, *J* = 8.6 Hz, 2H), 7.58 (d, *J* = 8.6 Hz, 2H). ^{13}C{^{1}H} NMR (100 MHz, $CDCl_3$) δ 153.6, 139.0, 130.5, 130.2, 129.0, 121.0, 108.2. HRMS (ESI): Calcd. for $C_9H_5N_3ClO$: 206.0116 $(M + H)^+$; Found: 206.0120.

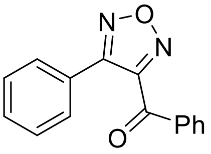

Phenyl(4-phenyl-1,2,5-oxadiazol-3-yl)methanone (3v)

Compound **3v** (62 mg, 82% yield) was purified with silica gel chromatography (PE/EtOAc = 50:1, R_f = 0.20) as yellow oil. ^{1}H NMR (400 MHz, $CDCl_3$) δ 8.09 (dd, *J* = 8.4, 1.2 Hz, 2H), 7.75 (dd, *J* = 8.2, 1.4 Hz, 2H), 7.70 (t, *J* = 7.4 Hz, 1H), 7.58 – 7.52 (m, 2H), 7.52 – 7.43 (m, 3H). ^{13}C{^{1}H} NMR (100 MHz, $CDCl_3$) δ 185.1, 154.3, 150.4, 135.6, 135.1, 131.0, 130.5, 128.94, 128.93, 128.8, 124.5. HRMS (ESI): Calcd. for $C_{15}H_{11}N_2O_2$: 251.0815 $(M + H)^+$; Found: 251.0820.

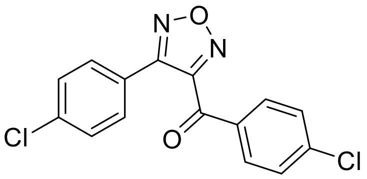

(4-Chlorophenyl)(4-(4-chlorophenyl)-1,2,5-oxadiazol-3-yl)methanone (3w)

Compound **3w** (73 mg, 77% yield) was purified with silica gel chromatography (PE/EtOAc = 50:1, R_f = 0.20) as a white solid. m.p. 90 ~ 93 °C. ^{1}H NMR (400 MHz, $CDCl_3$) δ 8.07 (d, J = 8.7 Hz, 2H), 7.72 (d, J = 8.7 Hz, 2H), 7.53 (d, J = 8.7 Hz, 2H), 7.46 (d, J = 8.6 Hz, 2H). ^{13}C{^{1}H} NMR (100 MHz, $CDCl_3$) δ 183.5, 153.7, 149.9, 142.1, 137.5, 133.8, 131.9, 130.4, 129.4, 129.3, 122.9. HRMS (ESI): Calcd. for $C_{15}H_9N_2O_2Cl_2$: 319.0036 $(M + H)^+$; Found: 319.0043.

5. General Procedure and Characterization Data of Compound 5.

To a 20 mL of Schlenk tube equipped with a stirrer was added β-monosubstituted enamine **4** (0.3 mmol, 1.0 equiv), O_2NO-I(III) **1d** (0.75 mmol, 262 mg, 2.5 equiv) and CuI (0.03 mmol, 6 mg, 10 mol%) under N_2 atmosphere, followed by addition of acetonitrile (4 mL). The tube was screw-capped and stirred at 60 °C. After stirring for 6 h, the reaction mixture was diluted with DCM, filtered through a pad of Celite and concentrated in vacuum. The residue was purified with silica gel chromatography (PE/EtOAc) to afford **5**.

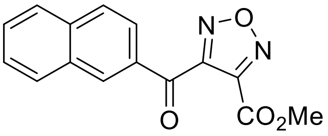

Methyl 4-(2-naphthoyl)-1,2,5-oxadiazole-3-carboxylate (5a)

Compound **5a** (63 mg, 75% yield) was purified with silica gel chromatography (PE/EtOAc = 15:1, R_f = 0.20) as light yellow oil. ^{1}H NMR (400 MHz, $CDCl_3$) δ 8.48 (s, 1H), 8.12 (dd, J = 8.7, 1.6 Hz, 1H), 7.97 (dd, J = 8.2, 5.8 Hz, 2H), 7.92 (d, J = 8.2 Hz, 1H), 7.68 (t, J = 7.5 Hz, 1H), 7.59 (t, J = 7.2 Hz, 1H), 3.98 (s, 3H). ^{13}C{^{1}H} NMR (100 MHz, $CDCl_3$) δ 182.4, 157.6, 151.8, 147.6, 136.5, 134.1, 132.2, 130.1, 129.9, 129.2, 127.9, 127.4, 123.8, 53.8. HRMS (ESI): Calcd. for $C_{15}H_{11}N_2O_4$: 283.0713 $(M + H)^+$; Found: 283.0703.

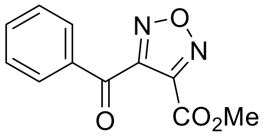

Methyl 4-benzoyl-1,2,5-oxadiazole-3-carboxylate (5b)

Compound **5b** (47 mg, 67% yield) was purified with silica gel chromatography (PE/EtOAc = 20:1, R_f = 0.20) as light yellow oil. ^{1}H NMR (400 MHz, $CDCl_3$) δ 8.03 (d, *J* = 8.5 Hz, 2H), 7.72 (t, *J* = 7.4 Hz, 1H), 7.56 (t, *J* = 7.8 Hz, 2H), 3.99 (s, 3H). ^{13}C{^{1}H} NMR (100 MHz, $CDCl_3$) δ 182.6, 157.6, 151.7, 147.6, 135.4, 134.8, 130.2, 129.1, 53.8. HRMS (ESI): Calcd. for $C_{11}H_9N_2O_4$: 233.0557 (M + H)$^+$; Found: 233.0560

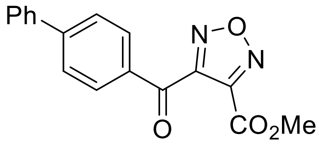

Methyl 4-([1,1'-biphenyl]-4-carbonyl)-1,2,5-oxadiazole-3-carboxylate (5c)

Compound **5c** (56 mg, 61% yield) was purified with silica gel chromatography (PE/EtOAc = 20:1, R_f = 0.20) as a white solid. m.p. 97 ~ 100 °C. ^{1}H NMR (400 MHz, $CDCl_3$) δ 8.11 (d, *J* = 8.5 Hz, 2H), 7.77 (d, *J* = 8.5 Hz, 2H), 7.65 (d, *J* = 7.0 Hz, 2H), 7.58 – 7.42 (m, 4H), 4.00 (s, 3H). ^{13}C{^{1}H} NMR (100 MHz, $CDCl_3$) δ 182.0, 157.6, 151.7, 148.1, 147.6, 139.2, 133.4, 130.8, 129.1, 128.8, 127.6, 127.3, 53.8. HRMS (ESI): Calcd. for $C_{17}H_{13}N_2O_4$: 309.0870 (M + H)$^+$; Found: 309.0879.

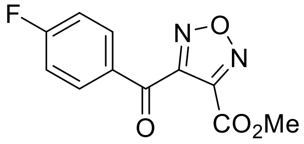

Methyl 4-(4-fluorobenzoyl)-1,2,5-oxadiazole-3-carboxylate (5d)

Compound **5d** (36 mg, 48% yield) was purified with silica gel chromatography (PE/EtOAc = 20:1, R_f = 0.20) as light yellow oil. ^{1}H NMR (400 MHz, $CDCl_3$) δ 8.13 – 8.07 (m, 2H), 7.23 (t, *J* = 8.6 Hz, 2H), 4.00 (s, 3H). ^{19}F NMR (376 MHz, $CDCl_3$) δ -100.19 – -100.28 (m, 1F). ^{13}C{^{1}H} NMR (100 MHz, $CDCl_3$) δ 180.8, 167.1 (d, *J* = 257.0 Hz), 157.6, 151.5, 147.6, 133.2 (d, *J* = 10.0 Hz), 131.2 (d, *J* = 3.0 Hz), 116.5 (d, *J* = 22.0 Hz), 53.9. HRMS (ESI): Calcd. for $C_{11}H_7FN_2O_4Na$: 273.0282 (M + Na)$^+$; Found: 273.0291.

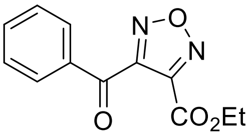

Ethyl 4-benzoyl-1,2,5-oxadiazole-3-carboxylate (5e)

Compound **5e** (47 mg, 64% yield) was purified with silica gel chromatography (PE/EtOAc = 20:1, R_f = 0.20) as light yellow oil. ^{1}H NMR (400 MHz, $CDCl_3$) δ 8.00 (d, *J* = 8.1 Hz, 2H), 7.69 (t, *J* = 7.4 Hz, 1H), 7.53 (t, *J* = 7.8 Hz, 2H), 4.41 (q, *J* = 7.1 Hz, 2H), 1.30 (t, *J* = 7.1 Hz, 3H). ^{13}C{^{1}H} NMR (100 MHz, $CDCl_3$) δ 182.7, 156.9, 151.7, 147.7, 135.3, 134.8, 130.1, 129.0, 63.4, 13.7. HRMS (ESI): Calcd. for $C_{12}H_{11}N_2O_4$: 247.0713 $(M + H)^+$; Found: 247.0725.

6. Gram-scale Synthesis of Product 3a.

To a 100 mL of Schlenk tube equipped with a stirrer was added enamine **2a** (10 mmol, 1.78 g, 1.0 equiv), O_2NO-I(III) **1d** (15 mmol, 5.35 g, 1.5 equiv) and CuI (1 mmol, 195 mg, 10 mol%) under N_2 atmosphere, followed by addition of acetonitrile (50 mL). The tube was screw-capped and stirred at 60 °C. After stirring for 24 h, the reaction mixture was diluted with DCM, filtered through a pad of Celite and concentrated in vacuum. The residue was purified with silica gel chromatography (PE/EtOAc) to afford **3a** (1.37 g, 67%).

7. Procedure for Synthesis of Methyl (4-(*p*-tolyl)-1,2,5-oxadiazole-3-carbonyl)glycinate (6).

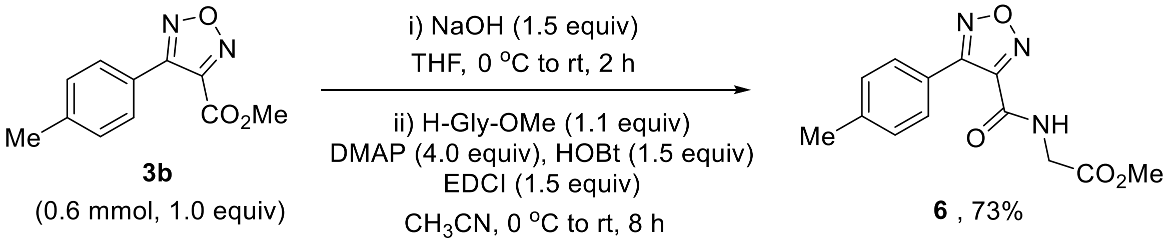

Compound **6** was prepared according to the previously reported procedure.[9] To a 25 mL tube equipped with a stirrer was added compound **3b** (0.6 mmol, 131 mg, 1.0 equiv) and THF (5 mL), a solution of NaOH (0.9 mmol, 360 mg, 1.5 equiv) in H_2O (2 mL) was added at 0 °C for 2 h. After the consumption of **3b**, the organic phase was concentrated, and the residue was extracted with EtOAc (3×10 mL). The organic phase was dried over Na_2SO_4, filtered, and concentrated. The residue was used for the next step without further purification. To a stirred solution of the residue in CH_3CN (8

mL) was added glycine methyl ester hydrochloride (0.66 mmol, 1.1 equiv), DMAP (2.4 mmol, 4.0 equiv), HOBt (0.9 mmol, 1.5 equiv) and EDCI (0.9 mmol, 1.5 equiv) at 0 °C. The resulting mixture was allowed to stir at room temperature for 8 h. The mixture was diluted with EtOAc (25 mL), washed with sat. aq. NH_4Cl (7 mL), brine (7 mL), dried over Na_2SO_4, filtered, and concentrated. The crude product was purified by column chromatography.

Methyl (4-(*p*-tolyl)-1,2,5-oxadiazole-3-carbonyl)glycinate (6)

Compound **6** (120 mg, 73% yield) was purified with silica gel chromatography (PE/EtOAc = 5:1, R_f = 0.20) as a white solid. m.p. 96 ~ 99 °C. ^{1}H NMR (400 MHz, $CDCl_3$) δ 7.82 (d, *J* = 8.2 Hz, 2H), 7.32 – 7.26 (m, 3H), 4.25 (d, *J* = 5.4 Hz, 2H), 3.81 (s, 3H), 2.41 (s, 3H). ^{13}C{^{1}H} NMR (100 MHz, $CDCl_3$) δ 169.2, 157.5, 154.1, 147.4, 141.3, 129.36, 129.32, 121.3, 52.6, 41.3, 21.4. HRMS (ESI): Calcd. for $C_{13}H_{14}N_3O_4$: 276.0979 $(M + H)^+$; Found: 276.089.

8. Procedure for Synthesis of Methyl 4-(4-azidophenyl)-1,2,5-oxadiazole-3-carboxylate (7).

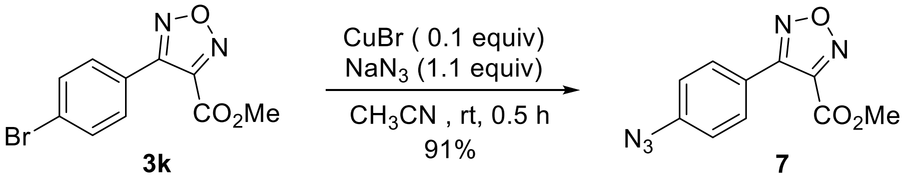

Compound **7** was prepared according to the previously reported procedure.[10] To a 25 mL tube equipped with a stirrer was added compound **3k** (0.3 mmol, 85 mg, 1.0 equiv), CuBr (0.03 mmol, 4.3 mg, 0.1 equiv), NaN_3 (0.33 mmol, 22 mg, 1.1 equiv) and CH_3CN (4 mL). The mixture was stirred at room temperature for 0.5 h. After the completion of the reaction, the mixture was concentrated *via* the rotary evaporation and the residue was purified with silica gel chromatography.

Methyl 4-(4-azidophenyl)-1,2,5-oxadiazole-3-carboxylate (7)

Compound **7** (67 mg, 91% yield) was purified with silica gel chromatography (PE/EtOAc = 25:1, R_f = 0.25) as a white solid. m.p. 83 ~ 85 °C. ^{1}H NMR (400 MHz, $CDCl_3$) δ 7.69 (d, *J* = 8.5 Hz, 2H), 7.62 (d, *J* = 8.5 Hz, 2H), 4.00 (s, 3H). ^{13}C{^{1}H} NMR (100 MHz, $CDCl_3$) δ 158.8, 153.5, 145.7, 131.9, 130.8, 125.8, 123.0, 53.5. HRMS (EI): Calcd. for $C_{10}H_8N_5O_3$: 246.0622 $(M + H)^+$; Found: 246.0617.

9. Preparation of compound 8.

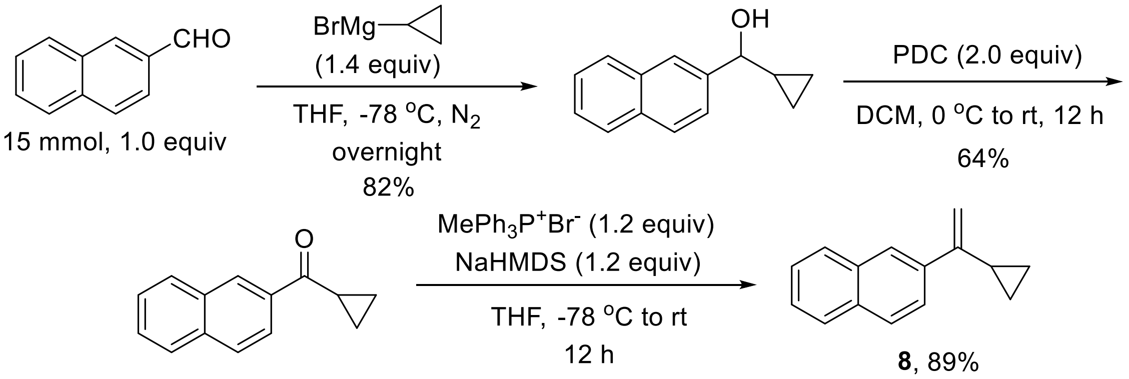

2-(1-Cyclopropylvinyl)naphthalene (**8**) was prepared according to the previously reported procedure.[11] A flame dried 250 mL round-bottom flask was equipped with a magnetic stir bar, was fit with a rubber septum, and backfilled with nitrogen three times. 2-naphthaldehyde (1.0 equiv, 2.34 g, 15 mmol) was added into the flask followed by the addition of 100 mL of dry THF. Freshly prepared methyl magnesium iodide (1.4 equiv, 21 mmol, 42 mL, M = 0.5) was slowly added to the aldehyde solution at -78 °C and stirred overnight. Excess of methyl magnesium iodide was quenched by adding 80 mL saturated NH_4Cl (aq.) and extracted with EtOAc (3 × 150 mL). Combined organic layers were dried over $MgSO_4$, filtered, and concentrated under reduced pressure. Pure compound was purified with silica gel chromatography (PE/EtOAc = 5:1, R_f = 0.25) to give cyclopropyl(naphthalen-2-yl)methanol as colorless oil (2.43 g, 82%). ^1H NMR (400 MHz, $CDCl_3$) δ 7.88 – 7.81 (m, 4H), 7.58 (dd, *J* = 8.6, 1.5 Hz, 1H), 7.52 – 7.44 (m, 2H), 4.18 (d, *J* = 8.3 Hz, 1H), 1.36 – 1.26 (m, 1H), 0.73 – 0.65 (m, 1H), 0.63 – 0.50 (m, 2H), 0.49 – 0.41 (m, 1H). $^{13}C\{^1H\}$ NMR (100 MHz, $CDCl_3$) δ 141.2, 133.3, 133.0, 128.1, 128.0, 127.7, 126.1, 125.8, 124.5, 124.4, 78.7, 19.2, 3.7, 2.9. Then cyclopropyl(naphthalen-2-yl)methanol (1.0 equiv, 2.43 g, 12.3 mmol) was dissolved in reagent grade 100 mL DCM and the resulting solution was cooled to 0 °C and PDC (24.6 mmol, 9.3 g, 2.0 equiv) was added portionwise (~1 g/min) with vigorous stirring. The solution was allowed to warm to room temperature where it was stirred for 12 hours. The reaction mixture was then filtered through a shourt plug of SiO_2 which was washed with DCM. After concentration of the filtrate, the residue was purified with silica gel chromatography (PE/EtOAc = 5:1, R_f = 0.25) to give cyclopropyl(naphthalen-2-yl)methanone as a colorless solid (1.54 g, 64%). ^1H NMR (400 MHz, $CDCl_3$) δ 8.57 (s, 1H), 8.07 (dd, *J* = 8.6, 1.7 Hz, 1H), 7.99 (d, *J* = 8.0 Hz, 1H), 7.90 (t, *J* = 8.6 Hz, 2H), 7.64 – 7.52 (m, 2H), 2.85 (tt, *J* = 7.8, 4.6 Hz, 1H), 1.35 – 1.28 (p, *J* = 3.6 Hz, 2H), 1.14 – 1.07 (dq, *J* = 7.2, 3.6 Hz, 2H). $^{13}C\{^1H\}$ NMR (100 MHz, $CDCl_3$) δ 200.5, 135.4, 135.3, 132.5, 129.54,

129.50, 128.3, 128.2, 127.7, 126.7, 124.0, 17.2, 11.7. A flame dried 250 mL round-bottom flask was equipped with a magnetic stir bar, was fit with a rubber septum, and backfilled with nitrogen three times. A solution of methyltriphenylphosphonium bromide (9.35 mmol, 3.34 g, 1.2 equiv) in anhydrous 30 mL THF was added into the flash, which was added dropwise the sodium bis(trimethylsilyl)amide (NaHDMS, 9.35 mmol, 9.35 mL of a 1.0 M solution in THF, 1.2 equiv) at 0 °C. After complete addition, the reaction mixture is stirred at 0 °C for additional 30 min, followed by the dropwise addition of a solution of cyclopropyl(naphthalen-2-yl)methanone (1.54 g, 7.8 mmol, 1.0 equiv) in 50 mL THF at -78 °C. The reaction mixture is allowed to warm to room temperature overnight and quensched by the addition of 10 mL water and saturated 30 mL NH_4Cl solution (aq.). The organic phase is separated, and the aqueous layer is extracted with EtOAc (3 × 100 mL). The combined organic phases are dried over $MgSO_4$ and concentrated under reduced pressure. The residue is purified with silica gel chromatography (PE/EtOAc = 10:1, R_f = 0.20) to give 2-(1-cyclopropylvinyl)naphthalene (**8**) as a white solid (1.35g, 89%). ^{1}H NMR (400 MHz, $CDCl_3$) δ 8.10 (s, 1H), 7.93 – 7.80 (m, 3H), 7.75 (d, *J* = 8.6 Hz, 1H), 7.54 – 7.44 (m, 2H), 5.46 (s, 1H), 5.08 (s, 1H), 1.89 – 1.73 (m, 1H), 0.99 – 0.88 (m, 2H), 0.73 – 0.65 (m, 2H). ^{13}C{^{1}H} NMR (100 MHz, $CDCl_3$) δ 149.1, 138.8, 133.3, 132.9, 128.3, 127.6, 127.5, 126.0, 125.8, 124.8, 124.6, 109.7, 15.7, 6.7.

10. Control Experiments.

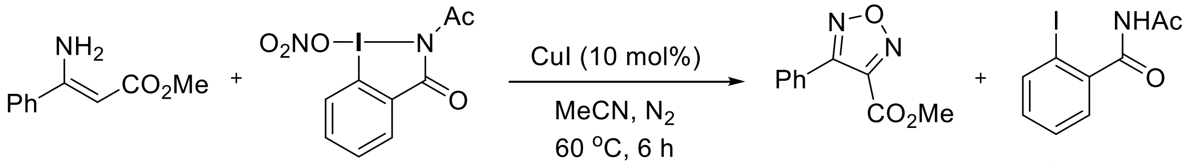

2a (1.0 equiv) **1d** (1.5 equiv) **3a**, 80% **S1**, 137%

Procedure: to a 20 mL of Schlenk tube equipped with a stirrer was added enamine **2a** (0.3 mmol, 53 mg, 1.0 equiv), compound **1d** (0.45 mmol, 158 mg, 1.5 equiv) and CuI (0.03 mmol, 6 mg, 10 mol%) under N_2 atmosphere, followed by addition of acetonitrile (4 mL). The tube was screw-capped and stirred at 60 °C. After stirring for 6 h, the reaction mixture was diluted with DCM, filtered through a pad of Celite and concentrated in vacuum. The residue was purified with silica gel chromatography (PE/EtOAc = 20:1 to 4:1) to afford **3a** as light yellow oil (49 mg, 80%) as well as co-product **S1** as a white solid (119 mg, 137%).

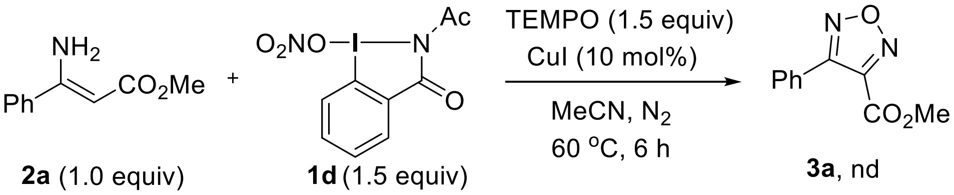

Procedure: to a 20 mL of Schlenk tube equipped with a stirrer was added enamine **2a** (0.3 mmol, 53 mg, 1.0 equiv), compound **1d** (0.45 mmol, 158 mg, 1.5 equiv), CuI (0.03 mmol, 6 mg, 10 mol%) and TEMPO (0.45 mmol, 70 mg, 1.5 equiv) under N_2 atmosphere, followed by addition of acetonitrile (4 mL). The tube was screw-capped and stirred at 60 °C. After stirring for 6 h, no **3a** was detected.

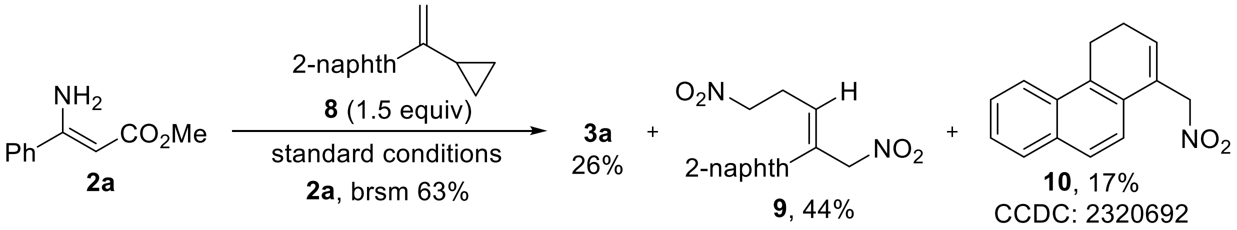

Procedure: to a 20 mL of Schlenk tube equipped with a stirrer was added enamine **2a** (0.3 mmol, 53 mg, 1.0 equiv), compound **1d** (0.45 mmol, 158 mg, 1.5 equiv), CuI (0.03 mmol, 6 mg, 10 mol%) and compound **8** (0.45 mmol, 87 mg, 1.5 equiv) under N_2 atmosphere, followed by addition of acetonitrile (4 mL). The tube was screw-capped and stirred at 60 °C. After stirring for 6 h, the reaction mixture was diluted with DCM, filtered through a pad of Celite and concentrated in vacuum. The residue was purified with silica gel chromatography (PE/EtOAc) to afford **3a** as light yellow oil (16 mg, 26%), **9** as orange oil (38 mg, 44%), **10** as a yellow solid (12 mg, 17%) as well as **2a** as a colorless solid (33 mg, 63%, brsm).

(*E*)-2-(1,5-Dinitropent-2-en-2-yl)naphthalene (9)

Compound **9** (38 mg, 44% yield) was purified with silica gel chromatography (PE/EtOAc = 30:1, R_f = 0.20) as orange oil. ^{1}H NMR (400 MHz, CD_3CN) δ 7.92 – 7.84 (m, 4H), 7.59 – 7.56 (m, 1H), 7.53 – 7.47 (m, 2H), 6.31 (t, *J* = 7.5 Hz, 1H), 5.53 (s, 2H), 4.64 (t, *J* = 6.5 Hz, 2H), 2.82 (q, *J* = 6.7 Hz, 2H). ^{13}C{^{1}H} NMR (100 MHz, CD_3CN) δ 137.5, 134.6, 134.3, 133.8, 133.4, 129.2, 129.1, 128.4, 127.5, 127.3, 126.0, 125.2, 73.3, 71.0, 27.2. HRMS (ESI): Calcd. for $C_{15}H_{15}N_2O_4$: 287.1026 (M + H)$^+$; Found: 287.1032.

1-(Nitromethyl)-3,4-dihydrophenanthrene (10)

Compound **10** (12 mg, 17% yield) was purified with silica gel chromatography (PE/EtOAc = 35:1, R_f = 0.20) as a yellow solid. m.p. 72 ~ 75 °C. ^{1}H NMR (400 MHz, $CDCl_3$) δ 8.07 (d, *J* = 8.4 Hz, 1H), 7.82 (d, *J* = 7.7 Hz, 1H), 7.74 (d, *J* = 8.6 Hz, 1H), 7.56 – 7.50 (m, 1H), 7.49 – 7.44 (m, 1H), 7.39 (d, *J* = 8.6 Hz, 1H), 6.41 (t, *J* = 4.6 Hz, 1H), 5.39 (s, 2H), 3.28 (t, *J* = 8.7 Hz, 2H), 2.56 (td, *J* = 8.7, 4.7 Hz, 2H). ^{13}C{^{1}H} NMR (100 MHz, $CDCl_3$) δ 135.4, 133.2, 131.7, 131.3, 128.9, 128.6, 128.3, 126.9, 126.4, 125.7, 123.6, 120.6, 78.3, 23.0, 22.4. HRMS (ESI): Calcd. for $C_{15}H_{13}NO_2Na$: 262.0839 (M + Na)$^+$; Found: 262.0858.

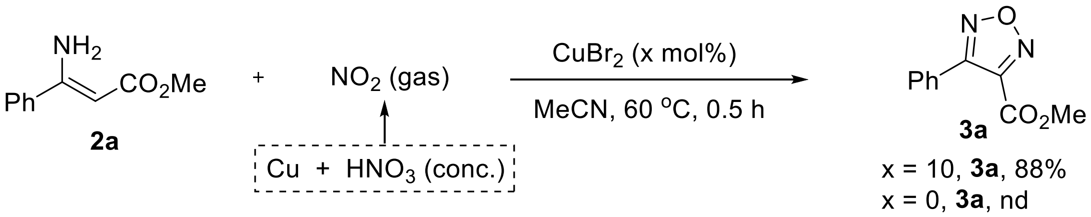

Procedure: to a 50 mL of three-necked round-bottomed flask equipped with a stirrer was added β-monosubstituted enamine **2a** (0.3 mmol, 53 mg, 1.0 equiv), $CuBr_2$ (x mol%) and NO_2 gas (prepared *in situ* from the reaction[12] of copper powder and HNO_3 (conc.) and as shown in the following picture), followed by addition of acetonitrile (4 mL). The tube was stirred at 60 °C for 30 min, the reaction mixture was diluted with DCM, filtered through a pad of Celite and concentrated in vacuum. When $CuBr_2$ (10 mol%) was used, the residue was purified with silica gel chromatography (PE/EtOAc = 25:1) to afford **3a** (54 mg, 88%), while no **3a** was observed when no $CuBr_2$ was used.

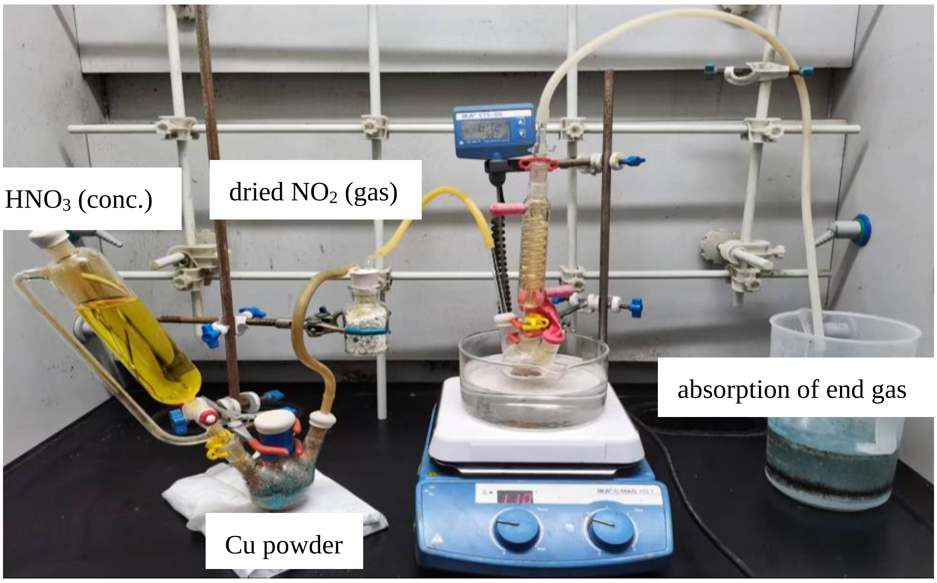

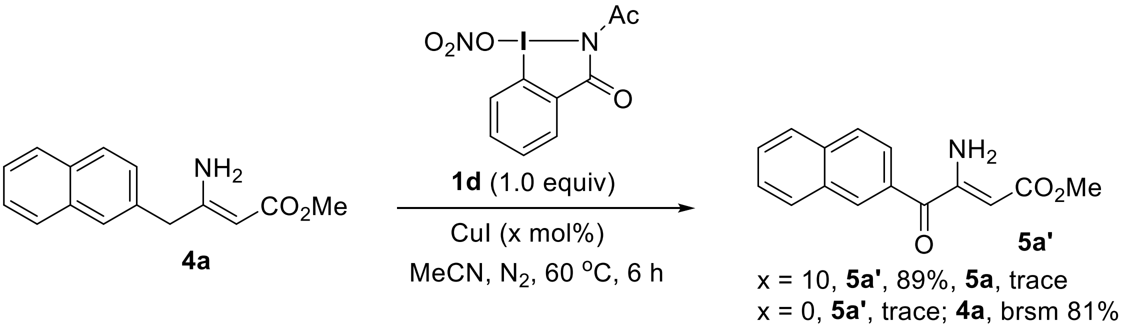

Procedure: to a 20 mL of Schlenk tube equipped with a stirrer was added enamine **4a** (0.3 mmol, 1.0 equiv), compound **1d** (0.3 mmol, 1.0 equiv), and CuI (x mol%) under N_2 atmosphere, followed by addition of acetonitrile (4 mL). The tube was screw-capped and stirred at 60 °C. After stirring for 6 h, the reaction mixture was diluted with DCM, filtered through a pad of Celite and concentrated in vacuum. The residue was purified with silica gel chromatography (PE/EtOAc = 12:1). When CuI (10 mol%) was used, **5a'** as a yellow solid (69 mg, 89%) as well as trace of **5a** was obtained respectively. when no CuI was used, **5a'** (trace) as well as **4a** (59 mg, 81%, brsm) was obtained respectively.

Methyl (*Z*)-3-amino-4-(naphthalen-2-yl)-4-oxobut-2-enoate (5a')

Compound **5a'** was purified with silica gel chromatography (PE/EtOAc = 12:1) as a yellow solid. m.p. 111 ~ 114 °C. ^{1}H NMR (400 MHz, CD_3CN) δ 8.33 (s, 1H), 7.98 – 7.81 (m, 4H), 7.63 (t, *J* = 7.0 Hz, 1H), 7.57 (t, *J* = 7.1 Hz, 1H), 5.20 (s, 1H), 3.73 (s, 3H). ^{13}C{^{1}H} NMR (100 MHz, CD_3CN) δ 193.1, 170.0, 152.0, 135.4, 132.6, 132.0, 131.5, 129.5, 128.7, 128.4, 127.8, 127.0, 125.1, 93.3, 51.0. HRMS (ESI): Calcd. for $C_{15}H_{14}NO_3$: 256.0968 $(M + H)^+$; Found: 256.0980.

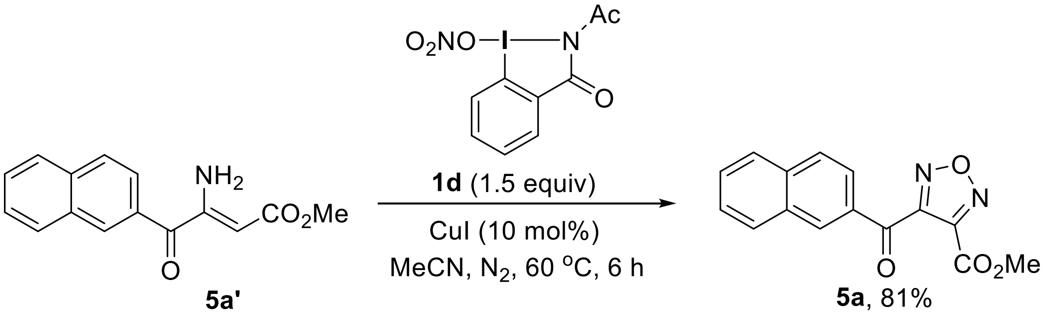

Procedure: to a 20 mL of Schlenk tube equipped with a stirrer was added enamine **5a'** (0.3 mmol, 1.0 equiv), compound **1d** (0.45 mmol, 1.5 equiv) and CuI (0.03 mmol, 6 mg, 10 mol%) under N_2 atmosphere, followed by addition of acetonitrile (4 mL). The tube was screw-capped and stirred at 60

°C. After stirring for 6 h, the reaction mixture was diluted with DCM, filtered through a pad of Celite and concentrated in vacuum. The residue was purified with silica gel chromatography (PE/EtOAc = 15:1) to afford **5a** as light yellow oil (69 mg, 81%).

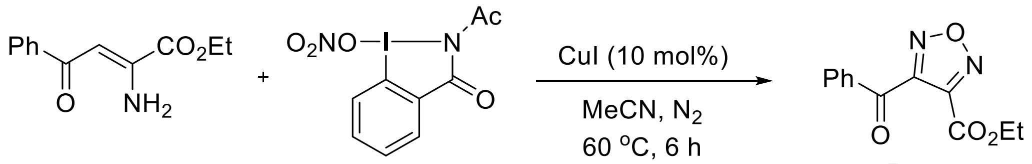

11 (1.0 equiv) **1d** (1.5 equiv) **5e**, 67%

Procedure: to a 20 mL of Schlenk tube equipped with a stirrer was added β-monosubstituted enamine **11** (0.3 mmol, 1.0 equiv), compound **1d** (0.45 mmol, 1.5 equiv) and CuI (0.03 mmol, 6 mg, 10 mol%) under N_2 atmosphere, followed by addition of acetonitrile (4 mL). The tube was screw-capped and stirred at 60 °C. After stirring for 6 h, the reaction mixture was diluted with DCM, filtered through a pad of Celite and concentrated in vacuum. The residue was purified with silica gel chromatography (PE/EtOAc = 20:1) to afford **5e** as light yellow oil (49 mg, 67%).

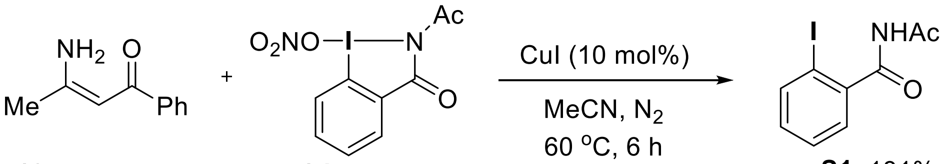

12 (1.0 equiv) **1d** (1.5 equiv) **S1**, 131%

Procedure: to a 20 mL of Schlenk tube equipped with a stirrer was added enamine **12** (0.3 mmol, 1.0 equiv), compound **1d** (0.45 mmol, 1.5 equiv) and CuI (0.03 mmol, 6 mg, 10 mol%) under N_2 atmosphere, followed by addition of acetonitrile (4 mL). The tube was screw-capped and stirred at 60 °C. After stirring for 6 h, the reaction mixture was diluted with DCM, filtered through a pad of Celite and concentrated in vacuum. The residue was purified with silica gel chromatography (PE/EtOAc = 4:1, R_f = 0.25) to afford **S1** as a white solid (114 mg, 131%).

III. FTIR spectrum of I(III)-ONO_2 1d.

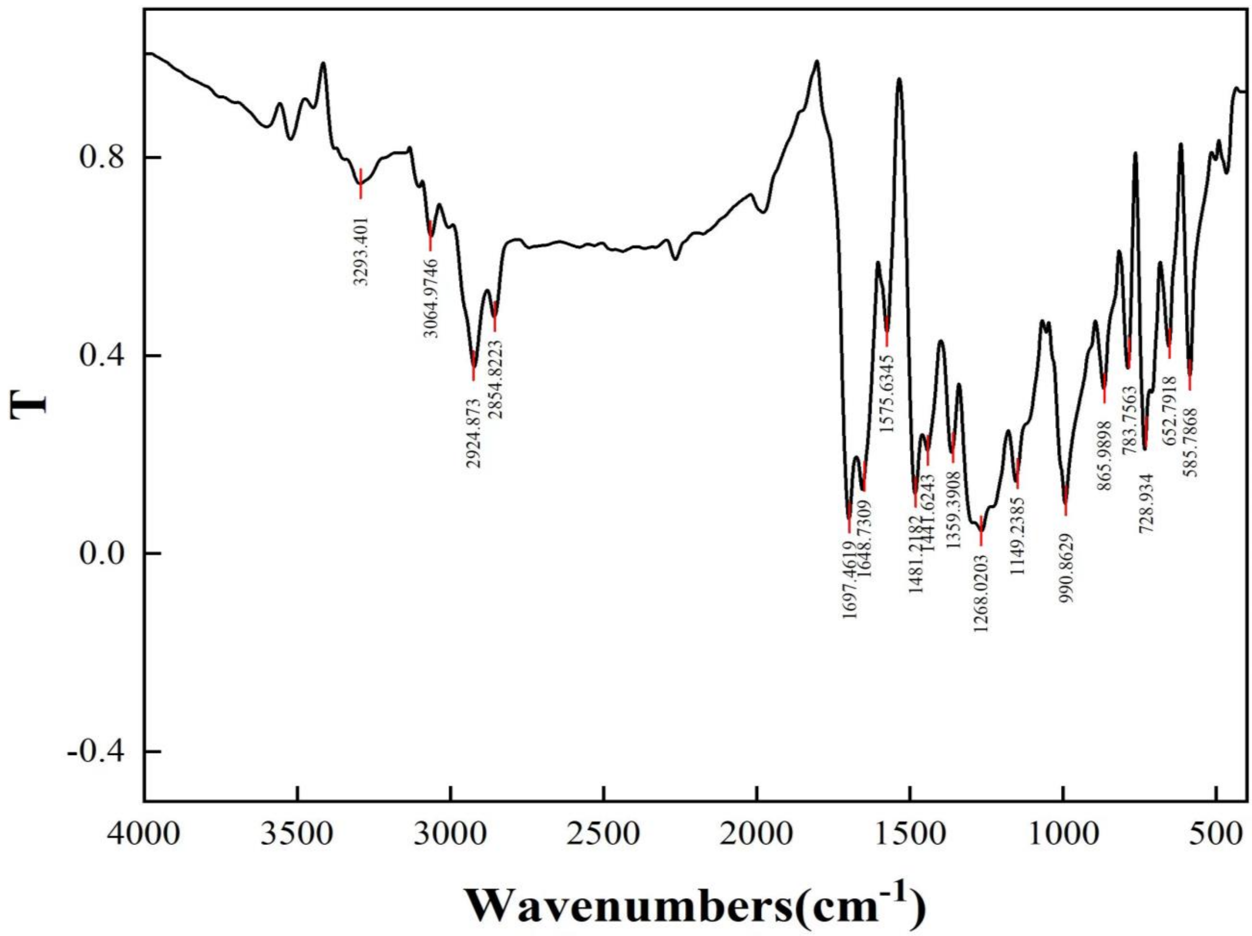

Figure S1. The FTIR spectrum of I(III)-ONO_2 **1d**.

IV. Thermogravimetric and Differential Scanning Calorimetry (TGA-DSC)

Profile of Compound 1d

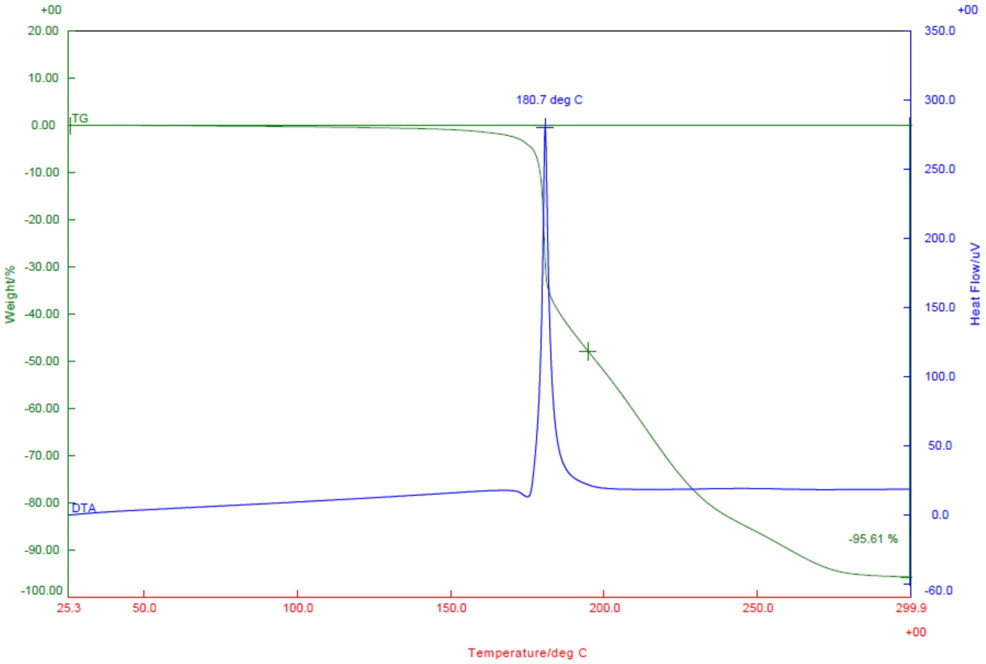

Figure S2. TGA-DSC profile of reagent **1d**. Thermogravimetric and differential scanning calorimetry (TGA-DSC) measurements were performed in order to determine the melting point and decomposition temperature of **1d**. Reagent **1d** does not have a melting point, and shows an exothermic decomposition at 180.7 °C, accompanied with a mass change of 95.6%.

V. X-Ray Diffraction Data of Compounds 1d, 3n and 10

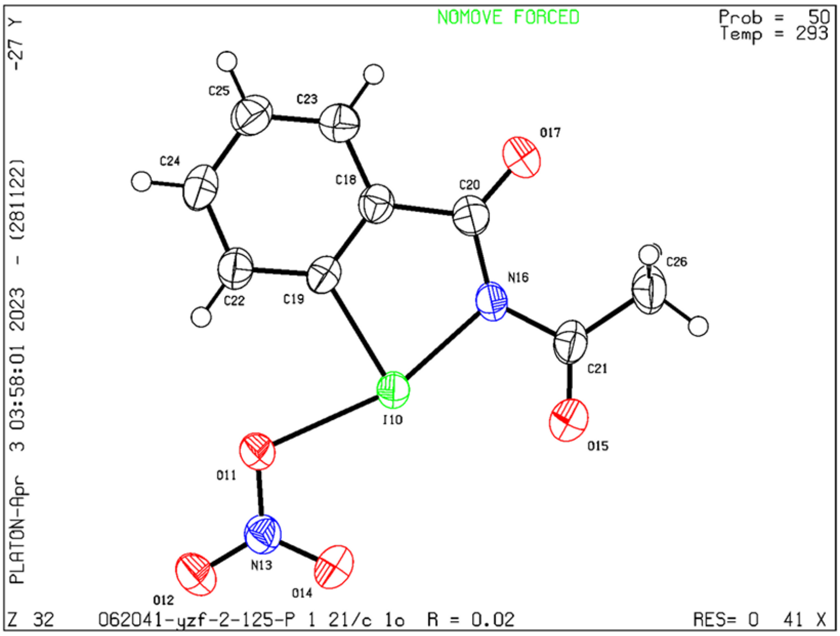

Figure S3. X-ray crystal structure of 1d with 50% ellipsoid probability.

Crystal Data for $C_9H_7IN_2O_5$ (M =350.07 g/mol): monoclinic, space group $P2_1/c$ (no. 14), a = 15.0319(2) Å, b = 5.87200(10) Å, c = 12.49510(10) Å, β = 98.6890(10)°, V = 1090.25(3) Å^3, Z = 4, T = 293(2) K, μ(Cu Kα) = 23.253 mm^{-1}, $Dcalc$ = 2.133 g/cm^3, 6653 reflections measured (5.948° ≤ 2Θ ≤ 152.122°), 2170 unique (R_{int} = 0.0232, R_{sigma} = 0.0222) which were used in all calculations. The final R_1 was 0.0210 (I > 2σ(I)) and wR_2 was 0.0578 (all data).

Table S2 Crystal data and structure refinement for 1d.

Identification code	062041-yzf-2-125-1-DJ-a_auto
Empirical formula	$C_9H_7IN_2O_5$
Formula weight	350.07
Temperature/K	293(2)
Crystal system	monoclinic
Space group	$P2_1/c$
a/Å	15.0319(2)
b/Å	5.87200(10)
c/Å	12.49510(10)
α/°	90
β/°	98.6890(10)
γ/°	90

Volume/Å^3	1090.25(3)
Z	4
ρ_{calc}g/cm^3	2.133
μ/mm^{-1}	23.253
F(000)	672.0
Crystal size/mm^3	0.25 × 0.02 × 0.02
Radiation	Cu Kα (λ = 1.54184)
2Θ range for data collection/°	5.948 to 152.122
Index ranges	-18 ≤ h ≤ 18, -5 ≤ k ≤ 7, -15 ≤ l ≤ 15
Reflections collected	6653
Independent reflections	2170 [R_{int} = 0.0232, R_{sigma} = 0.0222]
Data/restraints/parameters	2170/0/156
Goodness-of-fit on F^2	1.106
Final R indexes [I>=2σ (I)]	R_1 = 0.0210, wR_2 = 0.0571
Final R indexes [all data]	R_1 = 0.0219, wR_2 = 0.0578
Largest diff. peak/hole / e Å^{-3}	0.59/-0.56

Table S3 Fractional Atomic Coordinates (×10^4) and Equivalent Isotropic Displacement Parameters (Å^2×10^3) for 1d. U_{eq} is defined as 1/3 of the trace of the orthogonalised U_{IJ} tensor.

Atom	*x*	*y*	*z*	U(eq)
I10	2880.8(2)	4633.4(3)	6612.5(2)	34.09(10)
O11	1906.6(13)	2720(4)	5276.3(16)	42.5(5)
O12	1913.3(18)	1(4)	4120(2)	55.9(6)
N13	2316.7(16)	1012(4)	4902.6(19)	37.8(5)
O14	3069.1(16)	498(4)	5366(2)	54.0(6)
O15	4553.8(17)	4574(4)	7929(2)	55.3(6)
N16	3396.1(15)	6945(4)	7800.4(18)	37.4(5)
O17	3053.9(18)	10138(4)	8725(2)	54.0(6)
C18	1942.8(18)	8540(5)	7365(2)	34.8(6)
C19	1776.5(17)	6836(5)	6597(2)	32.3(5)
C20	2842.3(19)	8676(5)	8052(2)	38.2(6)
C21	4248.9(18)	6280(6)	8292(2)	40.4(6)
C22	960.0(18)	6628(6)	5922(2)	41.1(6)
C23	1262(2)	10091(5)	7472(3)	41.8(7)
C24	297.4(19)	8189(6)	6044(2)	44.6(7)
C25	442(2)	9898(5)	6815(3)	44.6(7)
C26	4715(2)	7654(7)	9204(3)	51.0(8)

Table S4 Anisotropic Displacement Parameters (Å^2×10^3) for 1d. The Anisotropic displacement factor exponent takes the form: $-2\pi^2[h^2a^{*2}U_{11}+2hka^*b^*U_{12}+\ldots]$.

Atom	U_{11}	U_{22}	U_{33}	U_{23}	U_{13}	U_{12}
I10	27.95(12)	41.51(14)	31.93(13)	-4.79(6)	1.66(8)	0.62(6)
O11	33.6(10)	51.4(12)	41.3(11)	-15.8(9)	2.0(8)	2.6(9)
O12	49.9(14)	63.8(14)	51.7(15)	-24.5(11)	0.8(12)	-1.4(11)
N13	32.2(12)	42.8(12)	38.7(13)	-3.7(11)	6.2(10)	-2.6(11)
O14	35.8(12)	59.2(15)	63.7(16)	-7.2(11)	-3.0(11)	9.9(10)
O15	40.2(13)	64.1(16)	57.9(15)	-12.6(12)	-4.2(11)	11.2(11)
N16	29.4(11)	47.9(13)	33.2(11)	-9.4(10)	-0.6(9)	-1.7(10)
O17	48.3(14)	56.2(13)	53.0(15)	-22.2(11)	-7.2(12)	3.0(11)
C18	33.9(13)	39.5(14)	30.6(13)	2.6(11)	3.5(10)	0.4(11)
C19	27.1(12)	39.3(13)	30.5(12)	4.1(11)	4.6(10)	3.0(11)
C20	36.0(14)	42.5(15)	35.8(14)	-2.1(12)	4.5(11)	-2.7(12)
C21	28.4(13)	54.3(18)	37.2(14)	-0.1(14)	1.3(11)	-1.1(13)
C22	33.6(14)	51.8(17)	36.5(14)	-2.7(13)	0.7(11)	2.1(13)
C23	42.2(18)	42.4(15)	41.0(17)	-3.0(12)	6.8(14)	2.9(13)
C24	31.3(14)	57.4(18)	43.0(16)	3.8(14)	-1.5(12)	3.0(14)
C25	38.6(17)	49.7(16)	45.9(18)	0.4(13)	7.8(15)	6.7(13)
C26	32.8(14)	73(2)	45.2(16)	-9.0(16)	-1.0(12)	-5.7(15)

Table S5 Bond Lengths for 1d.

Atom	Atom	Length/Å	Atom	Atom	Length/Å
I10	O11	2.3357(19)	O17	C20	1.210(4)
I10	N16	2.073(2)	C18	C19	1.382(4)
I10	C19	2.102(3)	C18	C20	1.491(4)
O11	N13	1.301(3)	C18	C23	1.392(4)
O12	N13	1.224(3)	C19	C22	1.386(4)
N13	O14	1.228(3)	C21	C26	1.483(4)
O15	C21	1.218(4)	C22	C24	1.379(4)
N16	C20	1.380(4)	C23	C25	1.378(5)
N16	C21	1.391(4)	C24	C25	1.386(5)

Table S6 Bond Angles for 1d.

Atom	Atom	Atom	Angle/°	Atom	Atom	Atom	Angle/°
N16	I10	O11	162.32(8)	C18	C19	I10	112.29(18)
N16	I10	C19	79.21(10)	C18	C19	C22	122.5(3)
C19	I10	O11	83.13(9)	C22	C19	I10	125.2(2)
N13	O11	I10	110.34(15)	N16	C20	C18	110.8(2)
O12	N13	O11	117.2(2)	O17	C20	N16	125.2(3)
O12	N13	O14	124.6(3)	O17	C20	C18	124.0(3)

Table S6 Bond Angles for 1d.

Atom	Atom	Atom	Angle/°	Atom	Atom	Atom	Angle/°
O14	N13	O11	118.2(2)	O15	C21	N16	116.1(3)
C20	N16	I10	118.20(17)	O15	C21	C26	124.9(3)
C20	N16	C21	130.8(2)	N16	C21	C26	118.9(3)
C21	N16	I10	110.62(19)	C24	C22	C19	117.5(3)
C19	C18	C20	119.5(2)	C25	C23	C18	119.6(3)
C19	C18	C23	118.8(3)	C22	C24	C25	121.3(3)
C23	C18	C20	121.7(3)	C23	C25	C24	120.4(3)

Table S7 Hydrogen Atom Coordinates (Å×10^4) and Isotropic Displacement Parameters (Å^2×10^3) for 1d.

Atom	*x*	*y*	*z*	U(eq)
H22	862.56	5478.65	5404.96	49
H23	1359.16	11250.67	7982.85	50
H24	-256.82	8092.24	5600.62	54
H25	-17.31	10920.16	6890.7	54
H26A	4376.64	7608.46	9796.84	76
H26B	4766.02	9201.24	8973.52	76
H26C	5305.32	7039.92	9433.64	76

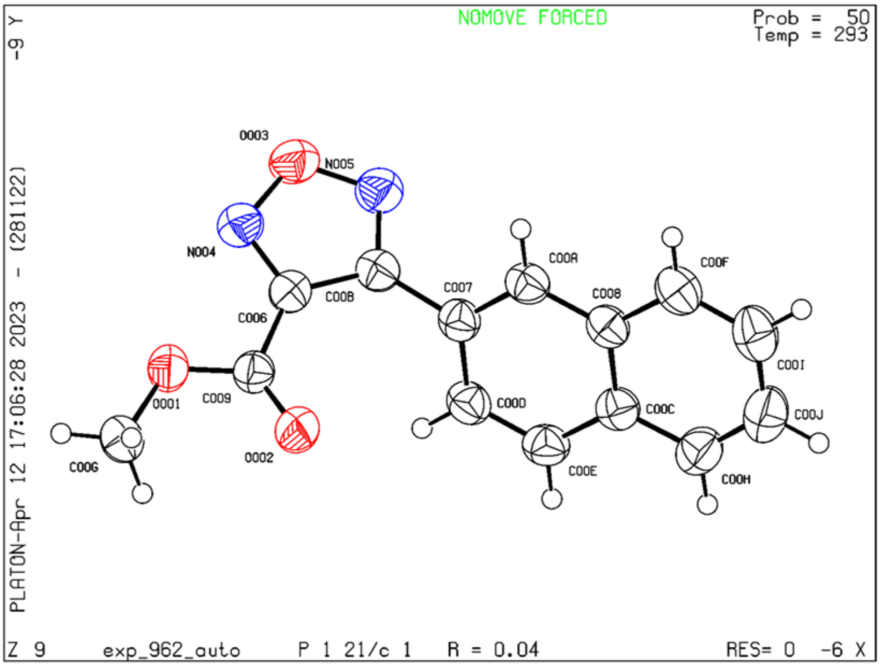

Figure S4. X-ray crystal structure of 3n with 50% ellipsoid probability.

Crystal Data for $C_{14}H_{10}N_2O_3$ (M =254.24 g/mol): monoclinic, space group P2$_1$/c (no. 14), a = 23.5669(4) Å, b = 6.12210(10) Å, c = 8.22150(10) Å, β = 90.955(2)°, V = 1186.02(3) Å^3, Z = 4, T = 293(2) K, μ(Cu Kα) = 0.851 mm^{-1}, $Dcalc$ = 1.424 g/cm^3, 6973 reflections measured (3.75° ≤ 2Θ ≤ 152.232°), 2339 unique (R_{int} = 0.0204, R_{sigma} = 0.0225) which were used in all calculations. The final R_1 was 0.0353 (I > 2σ(I)) and wR_2 was 0.1076 (all data).

Table S8 Crystal data and structure refinement for 3n.

Identification code	exp_962_auto
Empirical formula	$C_{14}H_{10}N_2O_3$
Formula weight	254.24
Temperature/K	293(2)
Crystal system	monoclinic
Space group	P2$_1$/c
a/Å	23.5669(4)
b/Å	6.12210(10)
c/Å	8.22150(10)
α/°	90
β/°	90.955(2)
γ/°	90
Volume/Å^3	1186.02(3)
Z	4
ρ_{calc}g/cm^3	1.424
μ/mm^{-1}	0.851
F(000)	528.0
Crystal size/mm^3	0.1 × 0.03 × 0.03
Radiation	Cu Kα (λ = 1.54184)
2Θ range for data collection/°	3.75 to 152.232
Index ranges	-29 ≤ h ≤ 26, -7 ≤ k ≤ 6, -10 ≤ l ≤ 9
Reflections collected	6973
Independent reflections	2339 [R_{int} = 0.0204, R_{sigma} = 0.0225]
Data/restraints/parameters	2339/0/174
Goodness-of-fit on F^2	1.050
Final R indexes [I>=2σ (I)]	R_1 = 0.0353, wR_2 = 0.1026
Final R indexes [all data]	R_1 = 0.0405, wR_2 = 0.1076
Largest diff. peak/hole / e Å^{-3}	0.15/-0.13

Table S9 Fractional Atomic Coordinates (×10^4) and Equivalent Isotropic Displacement Parameters (Å^2×10^3) for 3n. U_{eq} is defined as 1/3 of the trace of the orthogonalised U_{IJ} tensor.

**Atom**	***x***	***y***	***z***	**U(eq)**
O001	5602.9(4)	3061.8(16)	6288.6(12)	55.2(3)

Table S9 Fractional Atomic Coordinates (×10⁴) and Equivalent Isotropic Displacement Parameters (Å²×10³) for 3n. U_{eq} is defined as 1/3 of the trace of the orthogonalised U_{IJ} tensor.

Atom	*x*	*y*	*z*	U(eq)
O002	6466.1(4)	2215.5(17)	7303.6(13)	61.0(3)
O003	6272.9(4)	8463.7(16)	4486.4(12)	60.0(3)
N004	5965.8(5)	6750.5(18)	5090.4(14)	51.9(3)
N005	6848.9(5)	8126.4(19)	4729.2(15)	56.7(3)
C006	6339.4(5)	5388.4(19)	5682.2(14)	43.5(3)
C007	7463.7(5)	5414(2)	5940.8(14)	44.2(3)
C008	8371.8(5)	6070(2)	7274.4(14)	44.0(3)
C009	6154.7(5)	3360(2)	6521.4(15)	44.2(3)
C00A	7818.8(5)	6747(2)	6826.9(15)	45.0(3)
C00B	6897.4(5)	6246(2)	5462.3(15)	44.9(3)
C00C	8561.8(5)	3981(2)	6761.8(14)	45.9(3)
C00D	7649.6(5)	3316(2)	5455.5(16)	49.1(3)
C00E	8184.6(6)	2640(2)	5843.1(16)	50.0(3)
C00F	8739.4(6)	7405(2)	8221.3(16)	54.0(3)
C00G	5344.1(6)	1225(2)	7088.4(19)	56.5(3)
C00H	9121.6(6)	3333(2)	7197.2(18)	58.0(4)
C00I	9272.4(6)	6712(3)	8626.7(18)	63.9(4)
C00J	9464.3(6)	4661(3)	8103(2)	67.0(4)

Table S10 Anisotropic Displacement Parameters (Å²×10³) for 3n. The Anisotropic displacement factor exponent takes the form: $-2\pi^2[h^2a^{*2}U_{11}+2hka^*b^*U_{12}+\ldots]$.

Atom	U_{11}	U_{22}	U_{33}	U_{23}	U_{13}	U_{12}
O001	40.8(5)	58.1(6)	66.6(6)	12.6(4)	-2.6(4)	-3.2(4)
O002	49.5(5)	57.5(6)	75.7(7)	16.3(5)	-9.4(5)	-0.3(4)
O003	61.0(6)	52.3(5)	66.6(6)	13.6(4)	0.3(5)	5.5(4)
N004	52.9(6)	49.1(6)	53.5(6)	3.2(5)	-2.4(5)	2.5(5)
N005	55.2(6)	53.0(7)	62.2(7)	8.9(5)	5.8(5)	1.1(5)
C006	44.6(6)	44.5(6)	41.2(6)	-2.7(5)	-1.0(5)	2.3(5)
C007	43.3(6)	44.3(6)	45.1(6)	0.3(5)	6.5(5)	-2.2(5)
C008	46.8(6)	44.8(6)	40.4(6)	0.8(5)	5.7(5)	-4.8(5)
C009	42.1(6)	44.1(6)	46.4(6)	-2.2(5)	-1.3(5)	0.7(5)
C00A	48.3(7)	40.5(6)	46.4(6)	-2.3(5)	8.3(5)	-1.0(5)
C00B	47.9(6)	43.5(6)	43.6(6)	-1.8(5)	4.3(5)	-0.8(5)
C00C	47.3(6)	46.5(7)	44.1(6)	3.4(5)	5.9(5)	-0.3(5)
C00D	50.3(7)	43.6(7)	53.4(7)	-6.2(5)	2.9(5)	-4.7(5)
C00E	54.8(7)	40.9(6)	54.6(7)	-4.1(5)	6.9(5)	1.9(5)
C00F	57.9(8)	55.4(8)	48.6(7)	-4.1(6)	2.5(6)	-8.5(6)

Table S10 Anisotropic Displacement Parameters (Å^2×10^3) for 3n. The Anisotropic displacement factor exponent takes the form: -2π^2[h^{2}a*^{2}U$_{11}$+2hka*b*U$_{12}$+…].

Atom	U_{11}	U_{22}	U_{33}	U_{23}	U_{13}	U_{12}
C00G	48.2(7)	54.7(7)	66.8(8)	7.0(6)	3.5(6)	-6.1(6)
C00H	51.2(7)	60.8(8)	62.0(8)	3.6(7)	3.6(6)	7.6(6)
C00I	57.9(8)	75.7(10)	57.7(8)	-1.9(7)	-7.7(6)	-13.4(7)
C00J	49.2(7)	83.8(11)	67.8(9)	7.1(8)	-5.4(6)	1.5(7)

Table S11 Bond Lengths for 3n.

Atom	Atom	Length/Å	Atom	Atom	Length/Å
O001	C009	1.3240(14)	C007	C00D	1.4161(17)
O001	C00G	1.4428(16)	C008	C00A	1.4105(17)
O002	C009	1.1945(15)	C008	C00C	1.4210(18)
O003	N004	1.3720(14)	C008	C00F	1.4152(17)
O003	N005	1.3843(15)	C00C	C00E	1.4188(18)
N004	C006	1.3012(16)	C00C	C00H	1.4178(18)
N005	C00B	1.3035(17)	C00D	C00E	1.3600(18)
C006	C009	1.4894(17)	C00F	C00I	1.362(2)
C006	C00B	1.4302(17)	C00H	C00J	1.359(2)
C007	C00A	1.3700(17)	C00I	C00J	1.405(2)
C007	C00B	1.4761(17)			

Table S12 Bond Angles for 3n.

Atom	Atom	Atom	Angle/°	Atom	Atom	Atom	Angle/°
C009	O001	C00G	117.58(10)	O002	C009	C006	123.83(11)
N004	O003	N005	110.84(9)	C007	C00A	C008	121.13(11)
C006	N004	O003	105.52(10)	N005	C00B	C006	108.01(11)
C00B	N005	O003	106.05(10)	N005	C00B	C007	120.00(11)
N004	C006	C009	120.44(11)	C006	C00B	C007	131.97(11)
N004	C006	C00B	109.58(11)	C00E	C00C	C008	118.75(11)
C00B	C006	C009	129.88(11)	C00H	C00C	C008	118.26(12)
C00A	C007	C00B	118.53(11)	C00H	C00C	C00E	122.99(12)
C00A	C007	C00D	120.07(11)	C00E	C00D	C007	119.99(11)
C00D	C007	C00B	121.37(11)	C00D	C00E	C00C	121.25(12)
C00A	C008	C00C	118.78(11)	C00I	C00F	C008	120.55(14)
C00A	C008	C00F	121.88(12)	C00J	C00H	C00C	120.85(14)
C00F	C008	C00C	119.33(12)	C00F	C00I	C00J	120.26(14)
O001	C009	C006	109.99(10)	C00H	C00J	C00I	120.74(14)
O002	C009	O001	126.18(12)				

Table S13 Hydrogen Atom Coordinates (Å×10^4) and Isotropic Displacement Parameters (Å^2×10^3) for 3n.

**Atom**	*x*	*y*	*z*	**U(eq)**
H00A	7693.09	8122.32	7138.62	54
H00D	7405.73	2401.6	4871.76	59
H00E	8305.48	1272.14	5499.36	60
H00F	8616.04	8767.73	8569.62	65
H00B	4960.57	1057.31	6696.58	85
H00C	5344.36	1473.47	8241.32	85
H00G	5555.21	-76.83	6859.28	85
H00H	9255.98	1982.04	6858.89	70
H00I	9510.14	7600.5	9252.7	77
H00J	9829.96	4206.96	8379.05	80

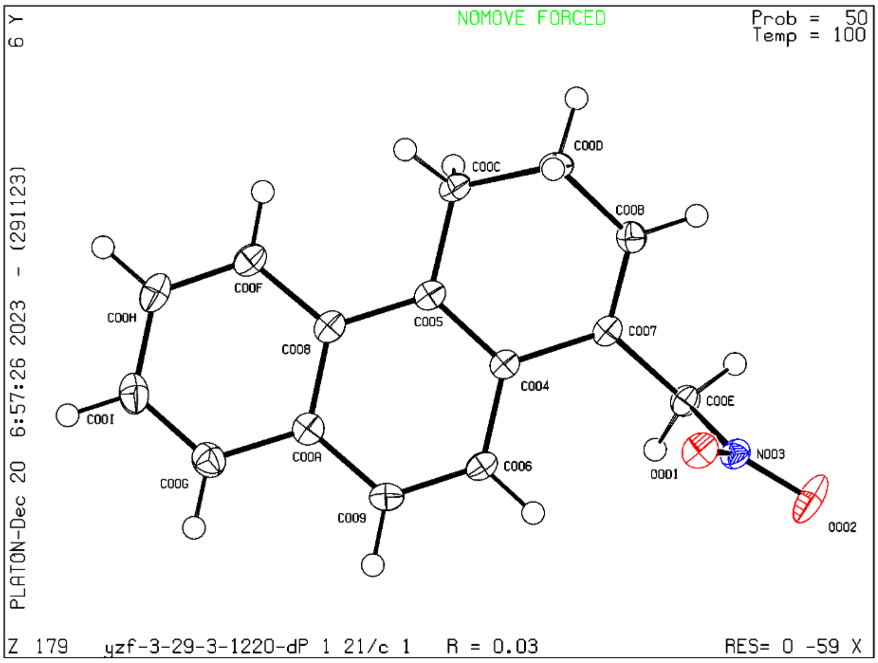

Figure S5. X-ray crystal structure of 10 with 50% ellipsoid probability.

Crystal Data for $C_{15}H_{13}NO_2$ (M =239.26 g/mol): monoclinic, space group P2$_1$/c (no. 14), a = 20.4165(3) Å, b = 8.17360(10) Å, c = 7.10370(10) Å, β = 94.1220(10)°, V = 1182.37(3) Å^3, Z = 4, T = 99.98(10) K, μ(Cu Kα) = 0.723 mm^{-1}, $Dcalc$ = 1.344 g/cm^3, 10868 reflections measured (4.34° ≤ 2Θ ≤ 144.528°), 2207 unique (R_{int} = 0.0215, R_{sigma} = 0.0162) which were used in all calculations. The final R_1 was 0.0329 (I > 2σ(I)) and wR_2 was 0.0908 (all data).

Table S14 Crystal data and structure refinement for 10.

Identification code	yzf-3-29-3-1220-DJ-a_auto
Empirical formula	$C_{15}H_{13}NO_2$

Formula weight	239.26
Temperature/K	99.98(10)
Crystal system	monoclinic
Space group	$P2_1/c$
a/Å	20.4165(3)
b/Å	8.17360(10)
c/Å	7.10370(10)
α/°	90
β/°	94.1220(10)
γ/°	90
Volume/Å^3	1182.37(3)
Z	4
ρ_{calc}g/cm^3	1.344
μ/mm^{-1}	0.723
F(000)	504.0
Crystal size/mm^3	0.3 × 0.02 × 0.02
Radiation	Cu Kα (λ = 1.54184)
2Θ range for data collection/°	4.34 to 144.528
Index ranges	-24 ≤ h ≤ 25, -9 ≤ k ≤ 9, -8 ≤ l ≤ 7
Reflections collected	10868
Independent reflections	2207 [R_{int} = 0.0215, R_{sigma} = 0.0162]
Data/restraints/parameters	2207/0/163
Goodness-of-fit on F^2	1.043
Final R indexes [I>=2σ (I)]	R_1 = 0.0329, wR_2 = 0.0889
Final R indexes [all data]	R_1 = 0.0356, wR_2 = 0.0908
Largest diff. peak/hole / e Å^{-3}	0.26/-0.21

Table S15 Fractional Atomic Coordinates (×10^4) and Equivalent Isotropic Displacement Parameters (Å^2×10^3) for 10. U_{eq} is defined as 1/3 of the trace of the orthogonalised U_{IJ} tensor.

**Atom**	***x***	***y***	***z***	**U(eq)**
O001	5876.5(4)	7982.9(10)	2906.3(11)	22.7(2)
O002	5029.6(4)	7130.9(12)	1197.4(13)	33.0(2)
N003	5560.6(4)	6904.9(12)	2066.6(12)	17.9(2)
C004	7080.4(5)	5244.1(13)	2970.3(15)	14.5(2)
C005	7601.2(5)	4544.9(13)	4054.7(15)	15.4(2)
C006	7205.3(5)	6179.0(13)	1345.7(15)	16.4(2)
C007	6400.6(5)	4937.9(13)	3488.7(15)	15.3(2)
C008	8252.2(5)	4662.5(13)	3450.5(15)	16.4(2)
C009	7828.6(5)	6369.8(14)	801.7(15)	18.2(2)
C00A	8366.2(5)	5601.6(14)	1815.6(15)	17.8(2)

Table S15 Fractional Atomic Coordinates (×10⁴) and Equivalent Isotropic Displacement Parameters (Å²×10³) for 10. U_{eq} is defined as 1/3 of the trace of the orthogonalised U_{IJ} tensor.

Atom	*x*	*y*	*z*	U(eq)
C00B	6290.4(5)	4395.7(13)	5213.5(15)	17.1(2)
C00C	7466.2(5)	3584.5(14)	5802.4(15)	17.6(2)
C00D	6846.3(5)	4172.3(14)	6680.6(15)	18.6(2)
C00E	5828.1(5)	5179.8(14)	2068.2(15)	17.0(2)
C00F	8794.0(5)	3823.4(15)	4384.6(16)	19.8(3)
C00G	9015.9(6)	5735.6(15)	1232.9(16)	22.7(3)
C00H	9408.6(6)	3941.4(16)	3751.3(17)	23.8(3)
C00I	9525.6(6)	4930.3(16)	2179.3(18)	25.4(3)

Table S16 Anisotropic Displacement Parameters (Å²×10³) for 10. The Anisotropic displacement factor exponent takes the form: $-2\pi^2[h^2a^{*2}U_{11}+2hka^*b^*U_{12}+\ldots]$.

Atom	U_{11}	U_{22}	U_{33}	U_{23}	U_{13}	U_{12}
O001	24.6(4)	18.7(4)	24.1(4)	-1.8(3)	-1.9(3)	-0.2(3)
O002	22.5(4)	39.4(6)	34.8(5)	-9.6(4)	-13.4(4)	14.1(4)
N003	17.1(5)	22.7(5)	13.6(4)	-1.0(4)	-0.7(3)	2.9(4)
C004	16.4(5)	12.3(5)	14.6(5)	-2.9(4)	-1.1(4)	-0.6(4)
C005	18.4(5)	12.1(5)	15.1(5)	-3.0(4)	-2.4(4)	-0.6(4)
C006	18.7(5)	14.8(5)	15.2(5)	-0.8(4)	-2.8(4)	1.6(4)
C007	16.0(5)	12.1(5)	17.4(5)	-2.0(4)	-2.2(4)	-0.1(4)
C008	17.9(5)	13.8(5)	17.0(5)	-4.1(4)	-3.0(4)	-0.8(4)
C009	22.7(6)	16.0(5)	15.9(5)	1.5(4)	0.5(4)	-0.9(4)
C00A	19.3(5)	15.7(5)	18.4(5)	-2.8(4)	0.0(4)	-1.1(4)
C00B	16.7(5)	15.6(5)	19.0(5)	-1.3(4)	0.8(4)	-2.1(4)
C00C	18.5(5)	17.1(5)	16.6(5)	1.8(4)	-3.2(4)	0.4(4)
C00D	22.2(5)	19.1(6)	14.4(5)	2.4(4)	-0.4(4)	-0.9(5)
C00E	16.2(5)	16.6(6)	17.8(5)	-1.3(4)	-1.5(4)	-0.2(4)
C00F	20.0(5)	18.8(6)	19.7(5)	-0.8(4)	-3.8(4)	0.9(4)
C00G	21.4(6)	24.7(6)	22.2(6)	-1.0(5)	2.5(4)	-3.1(5)
C00H	17.9(6)	26.3(6)	26.3(6)	-3.1(5)	-5.0(4)	3.8(5)
C00I	16.2(5)	32.1(7)	28.0(6)	-4.3(5)	2.1(5)	-1.4(5)

Table S17 Bond Lengths for 10.

Atom	Atom	Length/Å	Atom	Atom	Length/Å
O001	N003	1.2218(13)	C007	C00E	1.5013(14)
O002	N003	1.2224(12)	C008	C00A	1.4252(16)
N003	C00E	1.5122(15)	C008	C00F	1.4243(15)
C004	C005	1.3903(15)	C009	C00A	1.4157(16)

Table S17 Bond Lengths for 10.

Atom	Atom	Length/Å	Atom	Atom	Length/Å
C004	C006	1.4224(15)	C00A	C00G	1.4218(15)
C004	C007	1.4825(15)	C00B	C00D	1.4959(15)
C005	C008	1.4290(15)	C00C	C00D	1.5281(15)
C005	C00C	1.5109(15)	C00F	C00H	1.3666(16)
C006	C009	1.3652(15)	C00G	C00I	1.3666(17)
C007	C00B	1.3371(16)	C00H	C00I	1.4127(18)

Table S18 Bond Angles for 10.

Atom	Atom	Atom	Angle/˚	Atom	Atom	Atom	Angle/˚
O001	N003	O002	123.74(10)	C00F	C008	C005	122.39(10)
O001	N003	C00E	119.61(9)	C00F	C008	C00A	118.17(10)
O002	N003	C00E	116.65(9)	C006	C009	C00A	120.65(10)
C005	C004	C006	119.72(10)	C009	C00A	C008	119.17(10)
C005	C004	C007	119.03(10)	C009	C00A	C00G	121.54(10)
C006	C004	C007	121.19(10)	C00G	C00A	C008	119.29(10)
C004	C005	C008	119.79(10)	C007	C00B	C00D	120.64(10)
C004	C005	C00C	119.43(10)	C005	C00C	C00D	112.18(9)
C008	C005	C00C	120.68(9)	C00B	C00D	C00C	111.29(9)
C009	C006	C004	121.08(10)	C007	C00E	N003	112.77(9)
C004	C007	C00E	120.55(9)	C00H	C00F	C008	120.89(11)
C00B	C007	C004	120.38(10)	C00I	C00G	C00A	120.87(11)
C00B	C007	C00E	119.04(10)	C00F	C00H	C00I	120.80(11)
C00A	C008	C005	119.40(10)	C00G	C00I	C00H	119.89(11)

Table S19 Hydrogen Atom Coordinates (Å×10^4) and Isotropic Displacement Parameters (Å^2×10^3) for 10.

Atom	*x*	*y*	*z*	U(eq)
H006	6849.21	6678.87	627.48	20
H009	7902.29	7024.42	-267.77	22
H00B	5854.85	4149.94	5510.02	21
H00A	7845.92	3690.87	6742.44	21
H00C	7416.83	2412.48	5470.31	21
H00D	6719.47	3363.45	7625.9	22
H00E	6938.13	5223.76	7339.3	22
H00F	5474.26	4407.21	2345.86	20
H00G	5966.67	4920.07	794.82	20
H00H	8727.17	3172.95	5462.45	24
H00I	9096.69	6393.54	171.81	27

Table S19 Hydrogen Atom Coordinates (Å×10^4) and Isotropic Displacement Parameters (Å^2×10^3) for 10.

Atom	*x*	*y*	*z*	U(eq)
H00J	9761.48	3351.27	4375.65	29
H00K	9957.92	5035.37	1780.71	30

VI. Supplementary References

1) Yang, X.-G., Zheng, K., Zhang, C. Electrophilic hypervalent trifluoromethylthio-iodine(III) reagent. *Org. Lett.* **22**, 2026-2031, (2020).

2) Sun, X. Q.; Lyu, Y. R.; Zhang-Negrerie, D.; Du, Y.; Zhao, K. Formation of functionalized 2*H*-azirines through PhIO-mediated trifluoroethoxylation and azirination of enamines. *Org. Lett.* **15**, 6222-6225 (2013).

3) Zhang, Y.; Zhao, X.; Zhuang, C.; Wang, S.; Zhang-Negrerie, D.; Du, Y. PhIO/$Et_3N\cdot 3HF$-mediated formation of fluorinated 2*H*-azirines via domino fluorination/azirination reaction of enamines. *Adv. Synth. Catal.* **360**, 2107-2112 (2018).

4) Yang, J.-H.; Ji, C.-B.; Zhao, Y.-M.; Li, Y.-F.; Jiang, S.-Z.; Zhang, Z.-W.; Ji, Y.-Q.; Liu, W.-Y. $BF_3\cdot OEt_2$: an efficient catalyst for transesterification of β-ketoesters. *Synth. Commun.* **40**, 957-963 (2010).

5) Ji, Y.; Trenkle, W. C.; Vowles, J. V. A high-yielding preparation of β-ketonitriles. *Org. Lett*. **8**, 1161-1163 (2006).

6) Seifert, T.; Malo, M.; Kokkola, T.; Stéen, E. J. L.; Meinander, K.; Wallén, E. A. A.; Jarho, E. M.; Luthman, K. A scaffold replacement approach towards new sirtuin 2 inhibitors. *Bioorg. Med. Chem.* **28**, 115231-115242 (2020).

7) Ye, J.; Wang, C.; Chen, L.; Wu, X.; Zhou, L.; Sun, J. Chiral lewis base-catalyzed, enantioselective reduction of unprotected β-Enamino esters with trichlorosilane. *Adv. Synth. Catal*. **358**, 1042-1047 (2016).

8) Tabrizi, M. A.; Baraldi, P. G.; Baraldi, S.; Ruggiero, E.; De Stefano, L.; Rizzolio, F.; Mannelli, L. Di C.; Ghelardini, C.; Chicca, A.; Lapillo, M.; Gertsch, J.; Manera, C.; Macchia, M.; Martinelli, A.; Granchi, C.; Minutolo, F.; Tuccinardi, T. Discovery of 1,5-diphenylpyrazole-3-carboxamide derivatives as potent, reversible, and selective monoacylglycerol lipase (MAGL) inhibitors. *J. Med. Chem*. **61**, 1340-1354 (2018).

9) a) Yang, Z.-F.; Xu, C.; Zheng, X.; Zhang, X. Nickel-catalyzed carbodifunctionalization of *N*-vinylamides enables access to γ-amino acids. *Chem. Commun.* **56**, 2642-2645 (2020); b) Zhou, M.; Ni, C.; He, Z.; Hu, J. *O*-Trifluoromethylation of phenols: access to aryl trifluoromethyl ethers by *O*-carboxydifluoromethylation and decarboxylative fluorination. *Org. Lett*. **18**, 3754-3757

(2016).

10) Yang, X.; Wang, Z.; Fang, X.; Yang, X.; Wu, F.; Shen, Y. Synthesis of difluoromethylene-containing 1,2,4-oxadiazole compounds via the reaction of 5-(difluoroiodomethyl)-3-phenyl-1,2, 4-oxadiazole with unsaturated compounds initiated by sodium dithionite. *Synthesis,* **12**, 1768-1778 (2007).

11) He, T.; Wang, G.; Bonetti, V.; Klare, H. F. T.; Oestreich, M. Silylium-ion-promoted (5+1) cycloaddition of aryl-substituted vinylcyclopropanes and hydrosilanes involving aryl migration. *Angew. Chem. Int. Ed.* **59**, 12186-12191 (2020).

12) a) Kirovskaya, I. A.; Mironova, E. V.; Bykova, E. I.; Timoshenko, O. T.; Filatova, T. N. Adsorption and electrophysical studies of the sensitivity and selectivity of the surface of the InSb-CdTe system with respect to toxic gases. *Russ. J. Phys. Chem*. **82**, 830-834 (2008); b) Rilyanti, M.; Hadi, S. Synthesis, characterization and thermal stability of complex *cis*-$[Co(bipy)_2(CN)_2]$ and its interaction with NO_2 gas. *Russ. J. Inorg. Chem*. **56**, 418-421 (2011).
