# Supplementary material for: Preparation of a benziodazole-type iodine(III) compound and its application as a nitrating reagent for synthesis of furazans via a copper-catalyzed cascade process
Source: Commun Chem. 2024 Jul 9;7:155. doi: 10.1038/s42004-024-01238-8 (PMC11233585; doi:10.1038/s42004-024-01238-8)
Supplement: Supplementary file 3 — Description of Additional Supplementary Files [file 42004_2024_1238_MOESM3_ESM.pdf]

Description of Additional Supplementary Files

File name: Supplementary Data 1

Description: ^{1}H and ^{13}C NMR Spectra of Substrates and Products

File name: Supplementary Data 2

Description: FTIR spectra of compound **1d**

File name: Supplementary Data 3

Description: TGA-DSC profile of compound **1d**

File name: Supplementary Data 4

X-ray data file of compound **1d** (CCDC 2253476)

File name: Supplementary Data 5

X-ray data file of compound **3n** (CCDC 2255860)

File name: Supplementary Data 6

X-ray data file of compound **10** (CCDC 2320692)
