# Supplementary material for: Preparation of a benziodazole-type iodine(III) compound and its application as a nitrating reagent for synthesis of furazans via a copper-catalyzed cascade process
Source: Commun Chem. 2024 Jul 9;7:155. doi: 10.1038/s42004-024-01238-8 (PMC11233585; doi:10.1038/s42004-024-01238-8)
Supplement: Supplementary file 4 — Supplementary Data 1 [file 42004_2024_1238_MOESM4_ESM.zip › Supplementary Data 1.doc]

**^1^H and ^13^C NMR Spectra of Starting Materials and Products.**

**1d**

^1^H NMR (400 M, CDCl_3_)

**1d**

^13^C{^1^H} NMR (100 MHz, CDCl_3_)

**2a**

^1^H NMR (400 M, CDCl_3_)

**2a**

^13^C{^1^H} NMR (100 MHz, CDCl_3_)

**2b**

^1^H NMR (400 M, CDCl_3_)

**2b**

^13^C{^1^H} NMR (100 MHz, CDCl_3_)

**2c**

^1^H NMR (400 M, CDCl_3_)

**2c**

^13^C{^1^H} NMR (100 MHz, CDCl_3_)

**2d**

^1^H NMR (400 M, CDCl_3_)

**2d**

^13^C{^1^H} NMR (100 MHz, CDCl_3_)

**2e**

^1^H NMR (400 M, CDCl_3_)

**2e**

^13^C{^1^H} NMR (100 MHz, CDCl_3_)

**2f**

^1^H NMR (400 M, CDCl_3_)

**2f**

^19^F NMR (376 MHz, CDCl_3_)

**2f**

^13^C{^1^H} NMR (100 MHz, CDCl_3_)

**2g**

^1^H NMR (400 M, CDCl_3_)

**2g**

^19^F NMR (376 MHz, CDCl_3_)

**2g**

^13^C{^1^H} NMR (100 MHz, CDCl_3_)

**2h**

^1^H NMR (400 M, CDCl_3_)

**2h**

^19^F NMR (376 MHz, CDCl_3_)

**2h**

^13^C{^1^H} NMR (100 MHz, CDCl_3_)

**2i**

^1^H NMR (400 M, CDCl_3_)

**2i**

^13^C{^1^H} NMR (100 MHz, CDCl_3_)

**2j**

^1^H NMR (400 M, CDCl_3_)

**2j**

^13^C{^1^H} NMR (100 MHz, CDCl_3_)

**2k**

^1^H NMR (400 M, CDCl_3_)

**2k**

^13^C{^1^H} NMR (100 MHz, CDCl_3_)

**2l**

^1^H NMR (400 M, CDCl_3_)

**2l**

^13^C{^1^H} NMR (100 MHz, CDCl_3_)

**2m**

^1^H NMR (400 M, CDCl_3_)

**2m**

^13^C{^1^H} NMR (100 MHz, CDCl_3_)

**2n**

^1^H NMR (400 M, CDCl_3_)

**2n**

^13^C{^1^H} NMR (100 MHz, CDCl_3_)

**2o**

^1^H NMR (400 M, CDCl_3_)

**2o**

^13^C{^1^H} NMR (100 MHz, CDCl_3_)

**2p**

^1^H NMR (400 M, CDCl_3_)

**2p**

^13^C{^1^H} NMR (100 MHz, CDCl_3_)

**2q**

^1^H NMR (400 M, CDCl_3_)

**2q**

^13^C{^1^H} NMR (100 MHz, CDCl_3_)

**2r**

^1^H NMR (400 M, CDCl_3_)

**2r**

^13^C{^1^H} NMR (100 MHz, CDCl_3_)

**2s**

^1^H NMR (400 M, CDCl_3_)

**2s**

^13^C{^1^H} NMR (100 MHz, CDCl_3_)

**2t**

^1^H NMR (400 M, CDCl_3_)

**2t**

^13^C{^1^H} NMR (100 MHz, CDCl_3_)

**2u**

^1^H NMR (400 M, CDCl_3_)

**2u**

^13^C{^1^H} NMR (100 MHz, CDCl_3_)

**2v**

^1^H NMR (400 M, CDCl_3_)

**2v**

^13^C{^1^H} NMR (100 MHz, CDCl_3_)

**2w**

^1^H NMR (400 M, CDCl_3_)

**2w**

^13^C{^1^H} NMR (100 MHz, CDCl_3_)

**4a**

^1^H NMR (400 M, CDCl_3_)

**4a**

^13^C{^1^H} NMR (100 MHz, CDCl_3_)

**4b**

^1^H NMR (400 M, CDCl_3_)

**4b**

^13^C{^1^H} NMR (100 MHz, CDCl_3_)

**4c**

^1^H NMR (400 M, CDCl_3_)

**4c**

^13^C{^1^H} NMR (100 MHz, CDCl_3_)

**4d**

^1^H NMR (400 M, CDCl_3_)

**4d**

^19^F NMR (376 MHz, CDCl_3_)

**4d**

^13^C{^1^H} NMR (100 MHz, CDCl_3_)

**4e**

^1^H NMR (400 M, CDCl_3_)

**4e**

^13^C{^1^H} NMR (100 MHz, CDCl_3_)

**11**

^1^H NMR (400 M, CDCl_3_)

**11**

^13^C{^1^H} NMR (100 MHz, CDCl_3_)

**12**

^1^H NMR (400 M, CDCl_3_)

**12**

^13^C{^1^H} NMR (100 MHz, CDCl_3_)

**3a**

^1^H NMR (400 M, CDCl_3_)

**3a**

^13^C{^1^H} NMR (100 MHz, CDCl_3_)

**3b**

^1^H NMR (400 M, CDCl_3_)

**3b**

^13^C{^1^H} NMR (100 MHz, CDCl_3_)

**3c**

^1^H NMR (400 M, CDCl_3_)

**3c**

^13^C{^1^H} NMR (100 MHz, CDCl_3_)

**3d**

^1^H NMR (400 M, CDCl_3_)

**3d**

^13^C{^1^H} NMR (100 MHz, CDCl_3_)

**3e**

^1^H NMR (400 M, CDCl_3_)

**3e**

^13^C{^1^H} NMR (100 MHz, CDCl_3_)

**3f**

^1^H NMR (400 M, CDCl_3_)

**3f**

^19^F NMR (376 MHz, CDCl_3_)

**3f**

^13^C{^1^H} NMR (100 MHz, CDCl_3_)

**3g**

^1^H NMR (400 M, CDCl_3_)

**3g**

^19^F NMR (376 MHz, CDCl_3_)

**3g**

^13^C{^1^H} NMR (100 MHz, CDCl_3_)

**3h**

^1^H NMR (400 M, CDCl_3_)

**3h**

^19^F NMR (376 MHz, CDCl_3_)

**4a**

400 M, CDCl_3_

**3h**

^13^C{^1^H} NMR (100 MHz, CDCl_3_)

**3i**

^1^H NMR (400 M, CDCl_3_)

**3i**

^13^C{^1^H} NMR (100 MHz, CDCl_3_)

**3j**

^1^H NMR (400 M, CDCl_3_)

**3j**

^13^C{^1^H} NMR (100 MHz, CDCl_3_)

**3k**

^1^H NMR (400 M, CDCl_3_)

**3k**

^13^C{^1^H} NMR (100 MHz, CDCl_3_)

**3l**

^1^H NMR (400 M, CDCl_3_)

**3l**

^13^C{^1^H} NMR (100 MHz, CDCl_3_)

**3m**

^1^H NMR (400 M, CDCl_3_)

**3m**

^13^C{^1^H} NMR (100 MHz, CDCl_3_)

**3n**

^1^H NMR (400 M, CDCl_3_)

**3n**

^13^C{^1^H} NMR (100 MHz, CDCl_3_)

**3o**

^1^H NMR (400 M, CDCl_3_)

**3o**

^13^C{^1^H} NMR (100 MHz, CDCl_3_)

**3p**

^1^H NMR (400 M, CDCl_3_)

**3c**

400 M, CDCl_3_

**3p**

^13^C{^1^H} NMR (100 MHz, CDCl_3_)

**3q**

^1^H NMR (400 M, CDCl_3_)

**3q**

^13^C{^1^H} NMR (100 MHz, CDCl_3_)

**3r**

^1^H NMR (400 M, CDCl_3_)

**3r**

^13^C{^1^H} NMR (100 MHz, CDCl_3_)

**3s**

^1^H NMR (400 M, CDCl_3_)

**3s**

^13^C{^1^H} NMR (100 MHz, CDCl_3_)

**3t**

^1^H NMR (400 M, CDCl_3_)

**3t**

^13^C{^1^H} NMR (100 MHz, CDCl_3_)

**3u**

^1^H NMR (400 M, CDCl_3_)

**3u**

^13^C{^1^H} NMR (100 MHz, CDCl_3_)

**3v**

^1^H NMR (400 M, CDCl_3_)

**3v**

^13^C{^1^H} NMR (100 MHz, CDCl_3_)

**3w**

^1^H NMR (400 M, CDCl_3_)

**3w**

^13^C{^1^H} NMR (100 MHz, CDCl_3_)

**5a**

^1^H NMR (400 M, CDCl_3_)

**5a**

^13^C{^1^H} NMR (100 MHz, CDCl_3_)

**5a’**

^1^H NMR (400 M, CDCl_3_)

**5a’**

^13^C{^1^H} NMR (100 MHz, CDCl_3_)

**5b**

^1^H NMR (400 M, CDCl_3_)

**5b**

^13^C{^1^H} NMR (100 MHz, CDCl_3_)

**5c**

^1^H NMR (400 M, CDCl_3_)

**5c**

^13^C{^1^H} NMR (100 MHz, CDCl_3_)

**5d**

^1^H NMR (400 M, CDCl_3_)

**5d**

^19^F NMR (376 MHz, CDCl_3_)

**5d**

^13^C{^1^H} NMR (100 MHz, CDCl_3_)

**5e**

^1^H NMR (400 M, CDCl_3_)

**5d**

400 M, CDCl_3_

**5d**

400 M, CDCl_3_

**5e**

^13^C{^1^H} NMR (100 MHz, CDCl_3_)

**6**

^1^H NMR (400 M, CDCl_3_)

**6**

^13^C{^1^H} NMR (100 MHz, CDCl_3_)

**7**

^1^H NMR (400 M, CDCl_3_)

**7**

^13^C{^1^H} NMR (100 MHz, CDCl_3_)

**8**

^1^H NMR (400 M, CDCl_3_)

**8**

^13^C{^1^H} NMR (100 MHz, CDCl_3_)

**9**

^1^H NMR (400 M, CD_3_CN)

**9**

^13^C{^1^H} NMR (100 MHz, CD_3_CN)

**10**

^1^H NMR (400 M, CDCl_3_)

**10**

^3^C{^1^H} NMR (100 MHz, CDCl_3_)
