# Supplementary material for: Preparation of a benziodazole-type iodine(III) compound and its application as a nitrating reagent for synthesis of furazans via a copper-catalyzed cascade process
Source: Commun Chem. 2024 Jul 9;7:155. doi: 10.1038/s42004-024-01238-8 (PMC11233585; doi:10.1038/s42004-024-01238-8)
Supplement: Supplementary file 5 — Supplementary Data 2 [file 42004_2024_1238_MOESM5_ESM.zip › Supplementary Data 2.doc]

**FTIR spectrum of I(III)-ONO_2_ 1d.**

**Figure S1.** FTIR spectrum of I(III)-ONO_2_ **1d**.
