# Supplementary material for: Preparation of a benziodazole-type iodine(III) compound and its application as a nitrating reagent for synthesis of furazans via a copper-catalyzed cascade process
Source: Commun Chem. 2024 Jul 9;7:155. doi: 10.1038/s42004-024-01238-8 (PMC11233585; doi:10.1038/s42004-024-01238-8)
Supplement: Supplementary file 6 — Supplementary Data 3 [file 42004_2024_1238_MOESM6_ESM.zip › Supplementary Data 3.doc]

**TGA-DSC Profile of Compound** **1d**

**Figure S2.** TGA-DSC profile of reagent **1d**. Thermogravimetric and differential scanning calorimetry (TGA-DSC) measurements were performed in order to determine the melting point and decomposition temperature of **1d**. Reagent **1d** does not have a melting point, and shows an exothermic decomposition at 180.7 °C, accompanied with a mass change of 95.6%.
